# A novel putative genus phage CW39: implications for CRISPR-Cas9-based phage resistance in *Streptomyces avermitilis*

**DOI:** 10.3389/fmicb.2026.1850649

**Published:** 2026-06-26

**Authors:** Chang Wen, Yingying Wang, Lianrong Wang, Shi Chen

**Affiliations:** 1 Department of Gastroenterology, Hubei Clinical Center and Key Laboratory of Intestinal and Colorectal Disease, Ministry of Education Key Laboratory of Combinatorial Biosynthesis and Drug Discovery, School of Pharmaceutical Sciences, Zhongnan Hospital of Wuhan University, Wuhan University, Wuhan, China; 2 Department of Respiratory Diseases, Institute of Pediatrics, Shenzhen University Medical School, Shenzhen Children's Hospital, Shenzhen, China; 3 Shenzhen Key Laboratory of Microbiology in Genomic Modification and Editing and Application, Intensive Care Unit, Department of Critical Care Medicine, Shenzhen Institute of Translational Medicine, Shenzhen University Medical School, Shenzhen Second People's Hospital, The First Affiliated Hospital of Shenzhen University, Shenzhen, China

**Keywords:** CRISPR, genome, phage, resistance, *Streptomyces avermitilis*

## Abstract

*Streptomyces avermitilis* NRRL 8165 is an industrial model strain for the production of abamectin. Phage contamination during the fermentation process can lead to significant economic losses. At present, the known phages of *S. avermitilis* NRRL 8165 are scarce, making it difficult to meet the demands of research on pollution control mechanisms. Here, we isolated a novel phage, termed CW39, from soil samples. This phage exhibits a unique biological characteristic of solid-dependent infection. Transmission electron microscopy (TEM) revealed that the head measures approximately 65 nm in diameter, while the tail has a length of roughly 266 nm. Whole-genome sequencing revealed phage CW39 was 122,122 bp in genome size, exhibiting a GC content of 49.34%. Average nucleotide identity (ANI) analysis showed that phage CW39 shares only 64.4% nucleotide identity with its closest relative in the genus *Samistivirus*, which is significantly below the 70% genus-level classification threshold set forth by the International Committee on Taxonomy of Viruses (ICTV). Phylogenetic tree analysis further revealed that phage CW39 forms an independent monophyletic branch at the root. These findings indicate that phage CW39 likely represents a novel putative genus within the class Caudoviricetes. CRISPR-Cas9 plasmids targeting three key proteins of CW39 (the major capsid protein, head maturation protease, and portal protein) were separately transformed into *S. avermitilis* NRRL 8165, and all conferred enhanced phage resistance. This study not only enriches the *Streptomyces* phage resource library, but also provides a feasible strategy for using the CRISPR-Cas9 system to prevent and control phage contamination in industrial fermentation.

## 1 Introduction

*Streptomyces* is a genus of Gram-positive bacteria commonly found in soil and marine environments (Rey and Dumas, 2017). *Streptomyces* are well known for producing valuable secondary metabolites, including antibiotics, immune modulators, antitumor agents, and insect repellents, and these substances play important roles in human health, veterinary medicine, and agriculture (Jahan et al., 2025). Among them, *Streptomyces avermitilis* NRRL 8165 (ATCC 31267) is of particular industrial importance due to its production of avermectins (Takahashi et al., 2002). Large-scale liquid submerged fermentation of *S. avermitilis* is the primary method for avermectin production, but high energy consumption, high pollution, and high costs currently restrict its application in sustainable agriculture (Cadirci et al., 2016). Thus, researchers have proposed an alternative strategy: solid-state fermentation using agricultural waste (such as corn husks), which offers a greener and more efficient route for avermectin production (El-Housseiny et al., 2021). Under optimized conditions, the production cost of avermectin via solid-state fermentation was reduced by 8.38% compared with liquid submerged fermentation (Du et al., 2025). Thus, solid-state fermentation is emerging as a mainstream green strategy for industrial production using *S. avermitilis*.

With the rapid expansion of industrial fermentation, phage contamination has become one of the major threats to fermentation production. Once phages contaminate the fermentation system, they can lyse the host rapidly, leading to fermentation failure, a significant decrease in yield, and serious economic losses (Zou et al., 2025). As early as 1950, *Streptomyces griseus* was lysed by phages during streptomycin production (Koerber et al., 1950). The threat persists to this day: in 2024, novel phages isolated from soil, including Abafar and Scarif, infect up to 15 *Streptomyces* species (Rackow et al., 2024), indicating that this threat is becoming more serious. Elucidating phage-host interaction patterns is thus vital for effective prevention and control of phage contamination. Notably, the physical state of fermentation (liquid vs. solid) shapes phage-host interactions: liquid-grown *Streptomyces* form compact mycelial pellets that limit phage diffusion, whereas solid-state conditions create spatially structured mycelial networks that facilitate phage spread and multi-cycle infection (Yin, 1993; Abedon, 2008). Consistent with this phenotype, some *Streptomyces* phages (such as SAt2) propagate exclusively on solid medium but fail in liquid culture (Ogata et al., 1981). Our isolated phage CW39 also exhibits this solid-dependent infection trait, and therefore all infection assays were performed on solid medium to closely mimic industrial solid-state fermentation conditions.

To address the challenge of phage contamination in the fermentation industry, traditional physical and chemical disinfection methods are time-consuming and costly (Basak and Adak, 2024). Therefore, increasing attention has turned to biological anti-phage strategies, including our previously reported defense systems PT and Ppl (Wang et al., 2024; Xu et al., 2026). Notably, as the fundamental mechanism underlying bacterial adaptive immunity, the CRISPR-Cas system also is a powerful tool for combating phage infections. For example, an endogenous type I-E CRISPR-Cas system in *S. avermitilis* ATCC 31267 effectively helps the host resist phage infection, including the phages phiSASD1 and phiSAJS1 isolated from fermentation wastewater (Qiu et al., 2016), demonstrating the potential of this strategy for engineering phage-resistant strains. Besides this endogenous system, the heterologous CRISPR-Cas9 system has been frequently employed to counteract phage contamination in other industrial hosts. For example, it has been used to protect *Bacillus subtilis* 168 from phage SPP1 in the enzyme production industry (Jakutyte-Giraitiene and Gasiunas, 2016). Similar CRISPR-Cas9 strategies have also been successfully developed in *Escherichia coli* to combat phage infections, including a general programmable CRISPR-Cas9 system targeting T7 phage (Liu et al., 2020), a mobile CRISPR-Cas9 approach against filamentous phages (Cao et al., 2022), and a tailor-designed strategy targeting the newly isolated phage TR1 (Dong et al., 2023). However, to our knowledge, no study has reported the use of CRISPR-Cas9 for phage control in *S. avermitilis*, especially against solid-dependent phages that specifically threaten solid-state fermentation systems.

To address this gap in the literature, a novel solid-dependent phage, CW39, was isolated from soil using *S. avermitilis* NRRL 8165 as the host. A comprehensive analysis was performed on its morphological structure, host range, physiological characteristics, and whole genome. Phylogenetic trees and nucleotide similarity were then used to determine its taxonomic status. Furthermore, the CRISPR-Cas9 system was employed to target key genes in order to assess the resistance of *S. avermitilis* to this phage. This study therefore represents the first application of CRISPR-Cas9 to enhance resistance against a solid-dependent *Streptomyces* phage. This research not only expands the phage resource library of *S. avermitilis* but also provides a theoretical basis and practical strategies for controlling phage contamination in industrial solid fermentation systems.

## 2 Materials and methods

### 2.1 Strain, plasmid, and culture conditions

*S. avermitilis* NRRL 8165 was used as the host for phage isolation. *Escherichia coli* JM109 was used for transformation with recombinant plasmids. *E. coli* ET12567 served as the donor strain for intergeneric conjugation into *S. avermitilis*. The strains used for host range identification were as follows: *S. lividans* TK24 and its derivative strain *S. lividans* HXY6 (Liang et al., 2007); *Bacillus subtilis* 168; *Bacillus thuringiensis* CTC; *Pseudomonas aeruginosa* PAO1; *Pseudomonas fluorescens* Pf0-1; *E. coli* DH5α, BL21, DH10B, and MG1655. CRISPR-based anti-phage recombinant plasmids were constructed using the pCRISPomyces-2 backbone. MS solid medium (20 g/L each of mannitol, soybean powder, and agar) was used for *Streptomyces* strains, while LB liquid medium or LA solid medium (LB with 1.5% agar) was used for all other strains. Four antibiotics were used in this study. Apramycin was used for screening recombinant plasmid transformants, while kanamycin and chloramphenicol were employed to maintain the resident plasmids in *E. coli* ET12567. Nalidixic acid was used to inhibit *E. coli* ET12567 during conjugation and transfer. All strains were cultured at 37 °C, except for *Streptomyces* strains, which were incubated at 28 °C.

### 2.2 Phage isolation and purification

*S. avermitilis* NRRL 8165 phage CW39 was isolated from soil samples collected from agricultural soil in Chongqing, China (29°56′N, 106°40′E). Initially, 10 g of fresh soil was added to 90 mL sterile water, and the mixture was shaken at 220 rpm for 4 h. Subsequently, 1 mL of the mixture was centrifuged (12,000 rpm, 3 min), and the supernatant was filtered with 0.22 μm to obtain phage suspension. Briefly, 100 μL of phage suspension and 100 μL of *S. avermitilis* NRRL 8,165 spore suspension were mixed, followed by immediate addition of 10 mL of molten MS solid medium (approximately 55 °C). The combined mixture was then poured onto sterile petri dishes and incubated at 28 °C for 3 to 5 days to allow plaque formation. A sterile toothpick was used to pick a single plaque from the host lawn. The picked plaque was then streaked onto a fresh MS plate containing the host for further isolation, purification, and culture. This process was repeated 3 to 5 times to obtain a purified phage. Finally, the phage plaques on the host lawn were harvested with SM buffer, and the suspension was filtered through a 0.22-μm filter to obtain purified phage stocks. Phage titer was quantified via the standard single-layer spot assay (Paranos et al., 2024).

### 2.3 Transmission electron microscopy

The morphological characteristics of phage CW39 were analyzed via transmission electron microscopy (TEM). Firstly, 20 μL of phage suspension at a titer of 1 × 10^9^ PFU/mL was spotted onto a 200-mesh copper grid. The excess liquid was removed with filter paper after 5 min. Next, a single drop of 1% phosphotungstic acid was placed onto the grid for 10 min at room temperature. A Hitachi transmission electron microscope was used to observe the morphology of phage CW39. The acceleration voltage was set to 80 kV, and images were captured at a magnification of 30,000 × .

### 2.4 Optimal multiplicity of infection and multi-cycle phage accumulation on solid medium

Phage CW39 could not infect or lyse the host in liquid medium (TSB). However, it efficiently proliferated and formed clear plaques on solid medium. Therefore, we modified the traditional methods for determining the optimal multiplicity of infection (OMOI) to be performed on solid medium. First, the spore concentration of *S. avermitilis* NRRL 8165 was adjusted to 10^8^ CFU/mL. Then, 100 μL of the spore suspension and 10 mL of MS medium (approximately 55 °C) were mixed and poured onto a plate. The spores reached the optimal stage for phage infection after incubation at 28 °C for 4 h (Rosner and Gutstein, 1981). Meanwhile, phage CW39 10^9^ PFU/mL was serially diluted. For each MOI (10, 1, 0.1, 0.01, and 0.001), 100 μL of the phage dilution was quickly inoculated onto the germinated host lawn and evenly spread. The plates were air-dried and then incubated for 72 h at 28 °C. Next, 2 mL of SM buffer was added to each plate, and the surface was gently scraped with a spreader to elute the phages. The eluate was filtered through a 0.22 μm filter, serially diluted and subjected to plaque assays to calculate the phage titers. The MOI with the highest titer was defined as the OMOI.

To measure multi-cycle phage accumulation under the OMOI condition, multiple host plates were prepared and pre-germinated for 4 h as described above. The phage was quickly inoculated onto the host plate, evenly spread, and air-dried. Immediately after inoculation, the first set of plates was processed as the 0-h sample using the elution and filtration method described above and stored at 4 °C. The remaining plates were placed in an incubator at 28 °C. Due to the slow growth of *S. avermitilis* NRRL 8165, one set of plates was collected every 6 h over a 72 h incubation period. Phages from each time point were eluted and collected following the same procedure, and all samples were temporarily stored at 4 °C. Ultimately, the phages from all time points were serially diluted and subjected to spot assays to determine their titers, and the phage accumulation curve was plotted. Each experiment was repeated three times.

### 2.5 Stability analysis

For the thermal stability test, 1 mL of phage suspension was dispensed into sterile 2 mL EP tubes and incubated at 30, 40, 50, 60, and 70 °C for 1 h, respectively. For the ultraviolet sensitivity test, 1 mL of phage suspension was evenly spread in a sterile culture dish. The dish was then placed 30 cm away from an ultraviolet lamp and irradiated for 0, 3, 5, 15, 30, 45, and 60 min. To determine the tolerance of phage CW39 to different pH conditions, sterile SM buffers were prepared at a series of pH values (3, 4, 5, 6, 7, 8, 9, 10, 11, 12). Subsequently, 100 μL of phage suspension was mixed with 900 μL of the respective SM buffer and incubated at room temperature for 1 h (Ruizhe et al., 2025). Phage tolerance to organic solvents was determined by exposing the phage to methanol, ethanol, acetone, and chloroform (final volume fractions: 5%, 10%, 15%, 20%, and 30%) for 1 h (Cao et al., 2023). Finally, the phage titers were determined using the dilution spot method. Each experiment was repeated three times.

### 2.6 Host range assay

The high-titer phage CW39 (10^8^ PFU/mL) was serially diluted. Next, 2.5 μL of the phage dilution was spotted onto the host plates. Following incubation, plaque formation was observed, and the phage titer was calculated to assess the host range and relative infectivity of phage CW39. Each experiment was repeated three times.

### 2.7 Genome analysis

Genomic DNA of phage CW39 was extracted using the PEG precipitation method and sequenced on the Illumina platform. Raw reads were quality-filtered using SOAPnuke (Chen et al., 2017), and host reads were subsequently removed by aligning to the host genome using BWA v0.7.17 (Li and Durbin, 2009). Clean reads were assembled with MEGAHIT v1.2.9 (Li et al., 2015), and viral contigs were identified using CheckV v1.0.3 (Nayfach et al., 2021). The contig was considered circular if the overlap at both ends exceeded 50 bp with more than 94% identity. Gene prediction was performed with Prokka v1.14.6 (Seemann, 2014). Predicted proteins were annotated against the UniProtKB (Hulo et al., 2011), KEGG (Kanehisa and Goto, 2000), and COG (Tatusov et al., 2001) databases using BLAST+ v2.16.0 (Camacho et al., 2009) with an E-value cutoff of ≤ 1 × 10^−5^. Functional annotations from Prokka v1.14.6 were supplemented and cross-validated using the RAST server (Aziz et al., 2008) and Bakta v1.9.4 (Schwengers et al., 2021); conflicting annotations were manually checked and corrected. The phage genome map was plotted using Proksee (Grant et al., 2023). For comparative genomics, the most similar phage genome sequences were downloaded from the NCBI database, and collinearity analysis was calculated by Easyfig v2.2.5 (Sullivan et al., 2011). Phylogenetic trees for the major capsid protein and terminase of phage CW39 were built using MEGA v12 (neighbor-joining, 1,000 bootstrap replicates) (Grant et al., 2023). Average nucleotide identity (ANI) was calculated using taxMyPhage (Millard et al., 2025). The genome sequence was submitted to NCBI, which issued an accession number.

### 2.8 CRISPR-Cas9-mediated inhibition assay of phage CW39

The following genes were selected for CRISPR targeting: the major capsid protein and head maturation protease, which are essential for phage structural assembly, as well as the portal protein, which is involved in phage DNA packaging and genome delivery. The appropriate sgRNA was designed with Benchling. A pair of reverse complementary single-stranded oligonucleotide primers was synthesized, each containing a 20 bp sequence homologous to the vector at their 5′ and 3′ ends, and the two primers were annealed to form double-stranded DNA fragments (Vazquez et al., 2018). The pCRISPomyces-2 plasmid was digested with BbsI, and the digestion products were verified by electrophoresis and purified by gel extraction to obtain the linearized vector. The double-stranded spacer fragment was ligated with the purified linearized vector using the Gibson Assembly Master Mix (Cobb et al., 2015). The recombinant product was transformed into *E. coli* JM109, and positive clones were then screened on plates containing apramycin (25 μg/mL). Colonies were picked and verified by PCR using the specific primers pCP2-YZ-F/R (forward: 5′-TTCTGTGAATGGCGCTGTTCG-3′; reverse: 5′-TATAGTCCTGTCGGGTTCG-3′), which amplify a 404 bp fragment. Positive clones were further confirmed by sequencing to ensure correct spacer insertion.

The recombinant plasmid was transformed into *E. coli* ET12567, and transformants were selected on plates containing chloramphenicol, kanamycin, and apramycin (each at 25 μg/mL). Colonies were picked and verified by PCR and sequencing. The plasmid was transferred to *S. avermitilis* NRRL 8165 via conjugation. Positive strains were selected on MS plates containing apramycin (25 μg/mL) and nalidixic acid (50 μg/mL). Finally, the strain carrying the pCRISPomyces-2 empty plasmid was used as the negative control, and phage resistance mediated by CRISPR-Cas9 was preliminary evaluated by determining the phage titer using a spot assay. To quantify resistance, efficiency-of-plating (EOP) assays were performed. Phage CW39 (103 PFU/mL, 100 μL) was evenly spread onto MS plates pre-inoculated with either the recombinant strains or the empty-vector control. Plaques were counted after incubation at 28 °C for 5 days. Each strain was tested in triplicate. EOP was calculated as the PFU ratio (recombinant / empty-vector), and statistical significance was determined.

## 3 Results

### 3.1 Phage morphology

**Figure 1:**
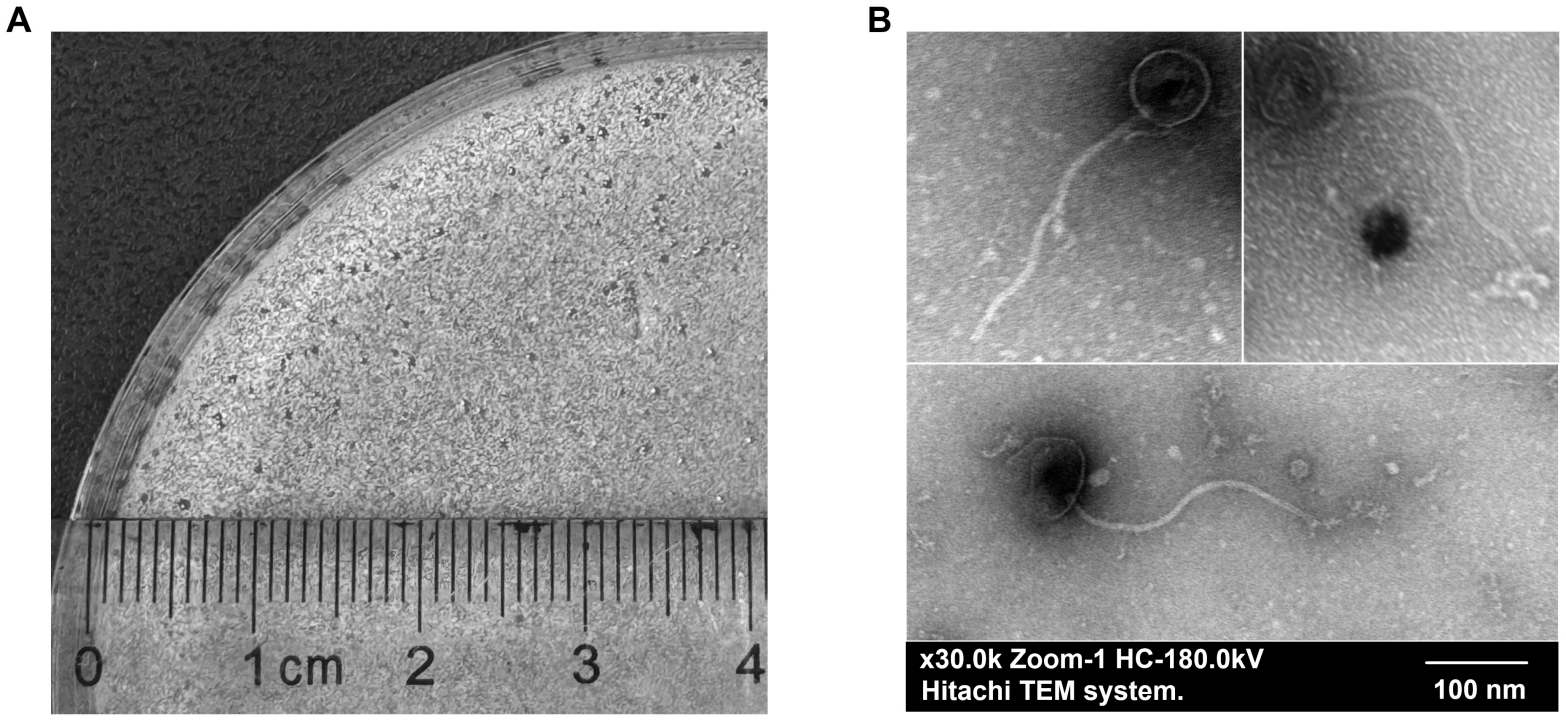
Morphology of phage CW39. **(A)** Single-plaque morphology of phage CW39 formed on MS medium after 72 h of incubation. **(B)** Morphology of phage CW39 observed through transmission electron microscopy (TEM).

After mixing spores with phage CW39 and incubating for 72 h at 28 °C, uniform, clear plaques of about 0.8 mm appeared (Figure 1A). TEM observation revealed that phage CW39 had a typical head-to-tail structure. Its head appeared approximately circular under two-dimensional projection, a typical imaging feature of icosahedral symmetry. Measurements indicated that the head diameter was approximately 65 nm, the tail length was about 266 nm, and the tail tube diameter was approximately 8 nm. The tail exhibited a long, curved, flexible filamentous tubular structure without a distinct contractile sheath (Figure 1B). The International Committee on Taxonomy of Viruses (ICTV) classification places phage CW39 in the class Caudoviricetes (Turner et al., 2023).

### 3.2 Optimal MOI and multi-cycle phage accumulation on solid medium

**Figure 2:**
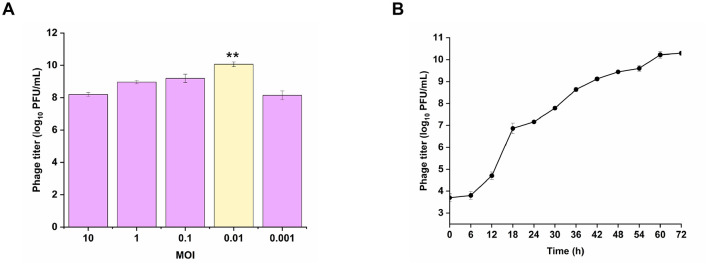
Optimal multiplicity of infection (OMOI) and multi-cycle accumulation of phage CW39 on solid MS medium. **(A)** Phage CW39 titers under different MOIs after 72 h of culture. **(B)** Time-dependent multi-cycle accumulation profile of phage CW39 at the OMOI of 0.01. Phage titers were detected at 6 h intervals and expressed as log10 PFU/mL. Data are presented as means ± standard deviations from three independent experiments. Statistical analysis was performed using one-way ANOVA followed by Tukey's multiple-comparison test. **p < 0.01.

To determine the optimal infection conditions of phage CW39 in *S. avermitilis* NRRL 8165, we first assessed phage titers across different MOIs, then monitored phage accumulation over time based on the optimal MOI (OMOI). At an MOI of 0.01, the phage titer peaked at approximately 10^10^ PFU/mL; MOIs lower or higher than 0.01 resulted in graded reductions in final titers (Figure 2A). This result confirms that the OMOI of CW39 is 0.01, which maximizes the synthesis and release of progeny phages. Using the OMOI of 0.01, we monitored phage CW39 propagation on solid MS medium over 72 h. The phage titer remained low (approximately 10^3^ PFU/mL) during the first 6 h post-inoculation. Between 6 and 60 h, the titer increased rapidly, reaching approximately 10^10^ PFU/mL. From 60 to 72 h, the titer plateaued and remained stable (Figure 2B). These measurements reflect multi-cycle phage accumulation rather than a classical one-step growth curve, because the assay was conducted on solid medium without removal of unadsorbed phages, allowing multiple rounds of infection and progeny release over time (Yin, 1993; Hyman and Abedon, 2009). Additionally, the relatively long proliferation cycle of CW39 on solid medium may be associated with both the slow growth rate and the mycelial morphological differentiation of *Streptomyces* (Rosner and Gutstein, 1981; Luthe et al., 2023).

### 3.3 Stability of phage CW39

**Figure 3:**
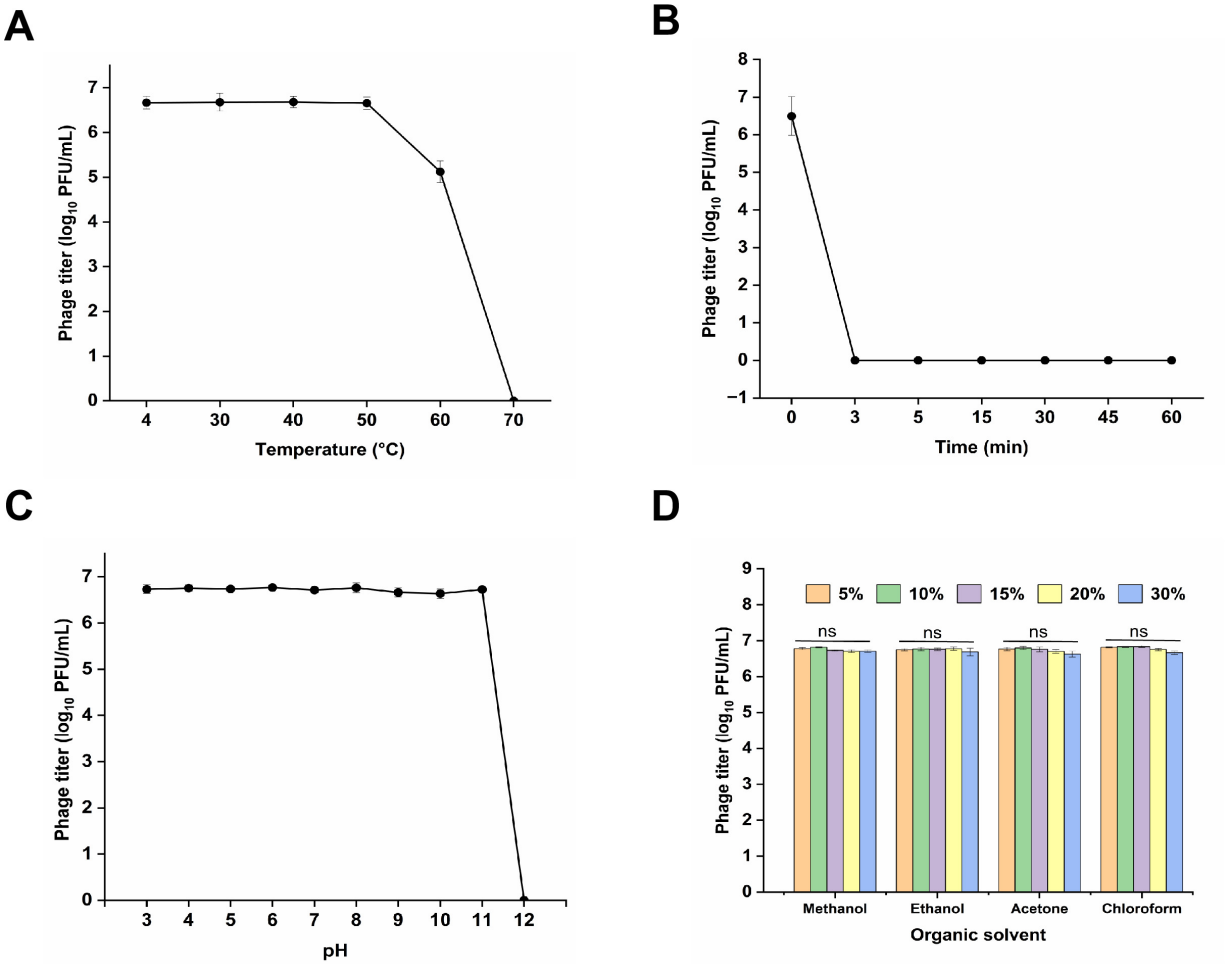
Physicochemical stability of phage CW39. **(A)** Thermal stability assessed after incubation at 4–70 °C for 1 h. **(B)** UV sensitivity determined under continuous irradiation over 0–60 min. **(C)** pH stability evaluated following incubation at pH values ranging from 3 to 12. **(D)** Stability against organic solvents at final volume fractions of 5%−30% (v/v). Phage titers were determined and expressed as log10 PFU/mL. Data are presented as means ± standard deviations from three independent experiments. Statistical analysis was performed using one-way ANOVA followed by Tukey's multiple-comparison test. ns, not significant (*p* > 0.05).

To evaluate the stability and application potential of phage CW39 under various environmental conditions, we characterized its key physiological characteristics, including temperature tolerance, UV sensitivity, pH stability, and organic solvent adaptability. The phage titer remained stable at approximately 10^6^ PFU/mL within the range of 4–50 °C. The titer fell sharply at 60 °C, and it was completely lost at 70 °C (Figure 3A). UV irradiation experiments revealed that CW39 was highly sensitive to ultraviolet light. After 3 min of exposure, its titer fell below the detection limit of the spot test, and no subsequent recovery was observed (Figure 3B). Regarding pH tolerance, CW39 maintained a stable titer of around 10^6^ PFU/mL across a broad range of pH 3–11, but was completely inactivated at pH 12 (Figure 3C). In organic solvent tolerance tests, CW39 exhibited high resistance to 5–30% volume fractions of methanol, ethanol, acetone, and chloroform, maintaining stable titers around 10^6^ PFU/mL (Figure 3D). This characteristic may confer an advantage in phage purification and genome extraction.

### 3.4 Host range of phage CW39

The host range of CW39 was preliminarily assessed by the spot test using available strains in our laboratory. *B. subtilis, Pseudomonas* spp., and *E. coli*, which are not *Streptomyces* species, were insensitive to CW39. In contrast, CW39 effectively infected its primary host, *S. avermitilis* NRRL 8165, with high titer (10^8^ PFU/mL), and also infected *S. lividans* HXY6, albeit at a lower titer of 10^4^ PFU/mL (Supplementary Table 1). Notably, CW39 did not infect *S. lividans* TK24 (Supplementary Figure 1). Based on the limited strains tested, CW39 appears to have a relatively narrow host range, infecting only certain *S. avermitilis* and *S. lividans strains*.

### 3.5 Genome features of CW39

Whole-genome sequencingrevealed that the CW39 genome was double-stranded and circular with a total length of 122,122 bp. The GC content (49.34%), represented by the black ring, was relatively uniform, suggesting stable base composition. The inner circle showed the GC skew distribution (green, positive; purple, negative), with clear transitions between positive and negative values around 30 kb and 90 kb, despite minor local variations. These two global inversion sites were consistent with the unidirectional θ-type replication of double-stranded DNA phages (Arakawa and Tomita, 2012), indicating that the replication initiation site was likely around 30 kb and the termination site around 90 kb. The genome was predicted to contain 208 open reading frames (ORFs). Based on gene function annotation, all ORFs were classified by function and marked with different colors on the outermost circle. The genome sequence and annotations have been deposited in NCBI under accession number PV558333.

**Figure 4:**
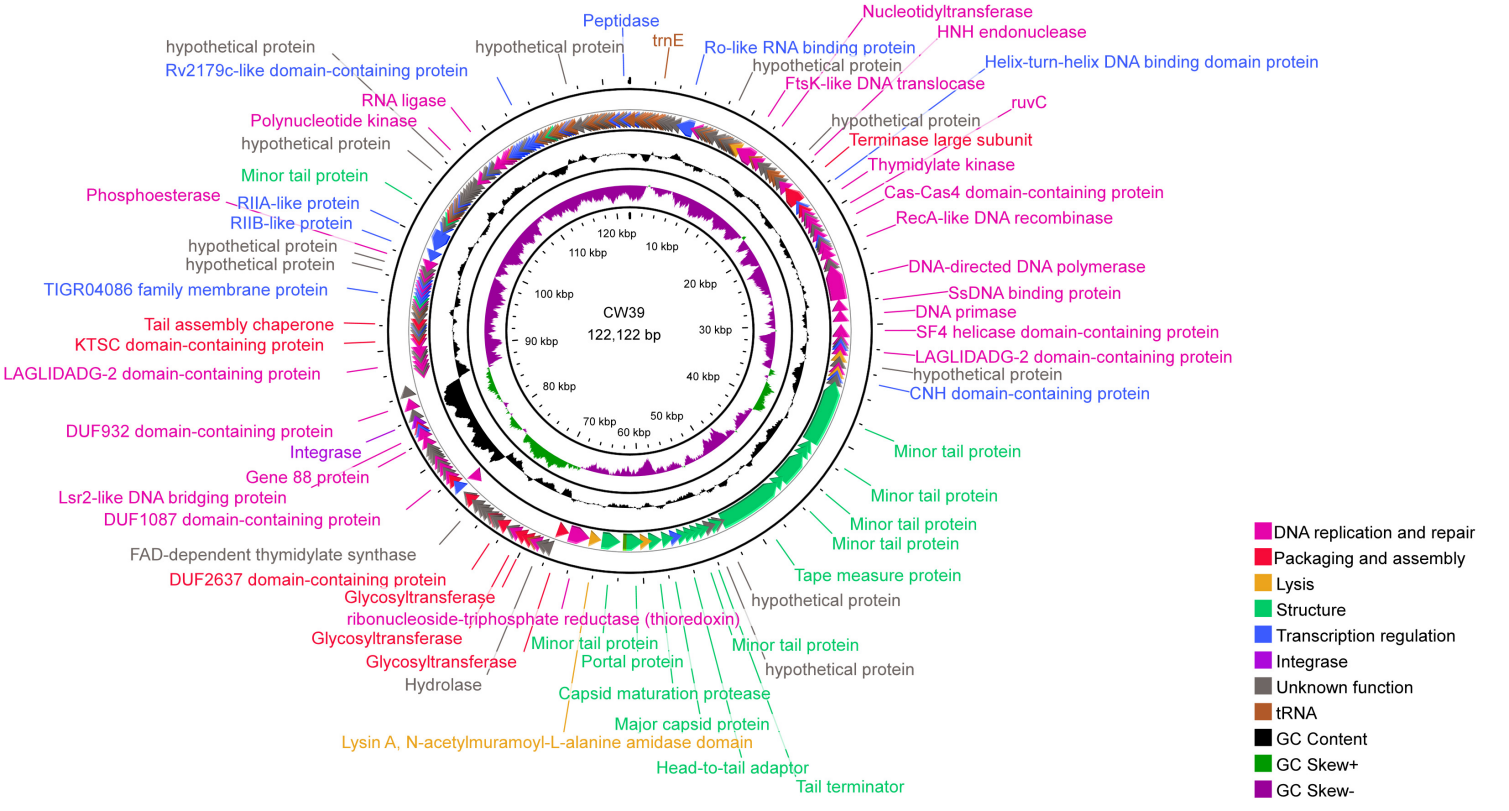
Genome map of phage CW39. The circular genome (122,122 bp) with predicted open reading frames (ORFs) was visualized using Proksee. The GC content and GC skew are shown as concentric rings. Annotated genes are color-coded according to their predicted functions.

The genome contained several functional modules. The DNA replication and repair module included key enzymes such as DNA-directed DNA polymerase, DNA primase, RecA-like DNA recombinase, and SF4 helicase. This compact gene arrangement enabled the phage to initiate genome replication and repair immediately after host infection (Nawel et al., 2022). The structural and packaging genes mainly included major capsid protein, minor tail protein, tail assembly chaperone, head-to-tail adaptor and tape measure protein. This clustering of structural genes facilitated efficient phage assembly and represented the core morphogenetic region (Lhuillier et al., 2009). HNH endonuclease was a key component of the phage DNA packaging machine (Kala et al., 2014). The lytic-related gene lysin A mediates host cell lysis and phage release. An integrase gene enables integration into the host genome via site-specific recombination (Nawel et al., 2022). Transcriptional regulation and hypothetical proteins were distributed across various functional regions. Nucleotidyltransferase, Cas-Cas4 domain-containing protein, and RuvC were typically involved in DNA repair or RNA metabolism. Multiple glycosyltransferases and FAD-dependent thymidylate synthase, along with other metabolism-related genes, participated in host nucleotide metabolism and cell wall synthesis (Thompson et al., 2011), creating favorable conditions for phage replication (Figure 4).

### 3.6 Phylogenetic analysis, genome collinearity and ANI comparison

**Figure 5:**
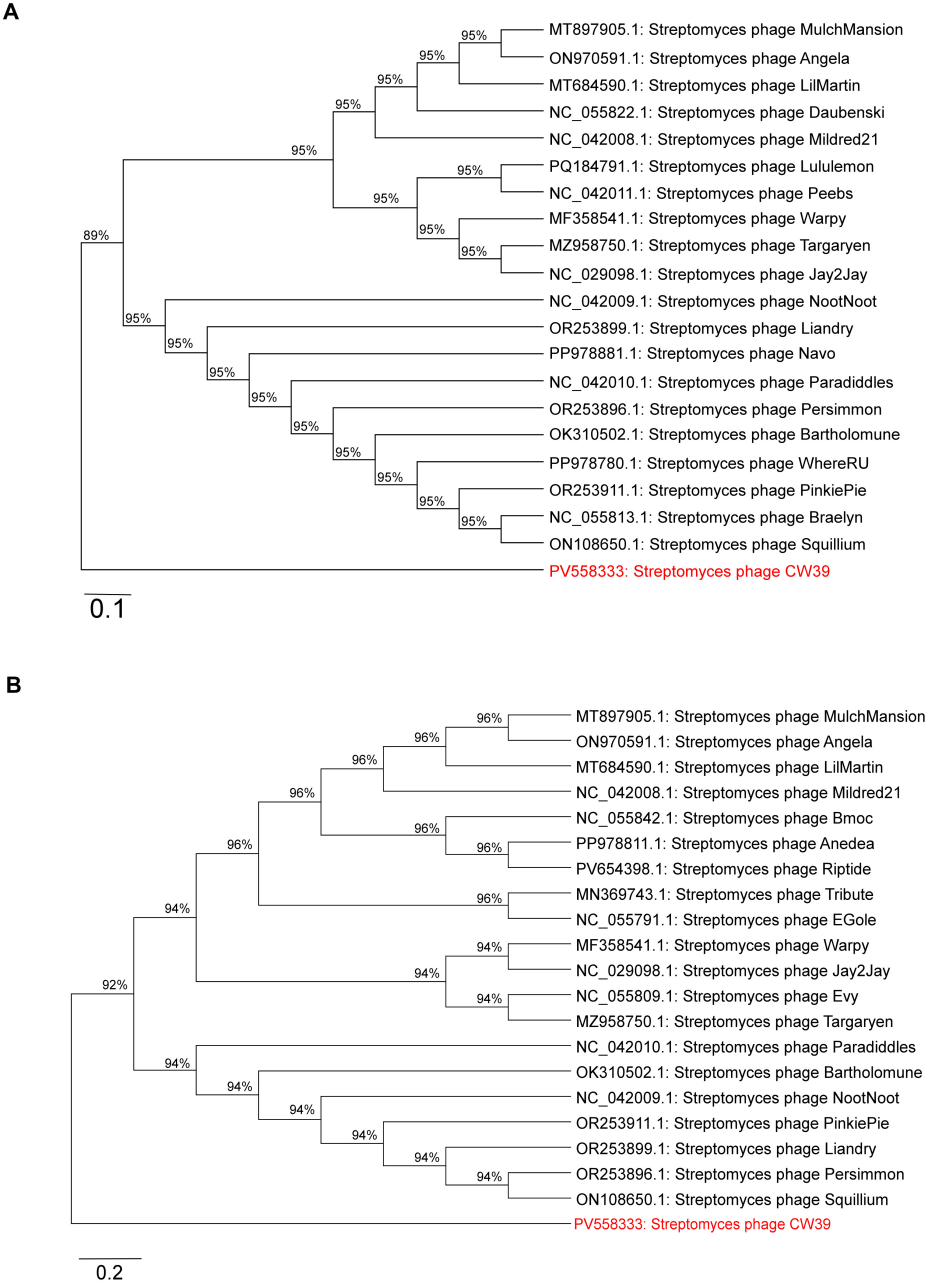
Phylogenetic analysis of phage CW39. **(A)** Phylogenetic tree based on the major capsid protein. **(B)** Phylogenetic tree based on the terminase. Related sequences were obtained from the NCBI database. Phage CW39 is highlighted in red. The scale bars indicate 0.1 **(A)** and 0.2 **(B)** respectively.

To determine the taxonomic position of phage CW39, two key proteins (the major capsid protein and terminase) were selected for the construction of phylogenetic trees. Evolutionary analysis of these two core proteins revealed that phage CW39 occupied a consistent phylogenetic position, forming independent monophyletic branches at the base of the tree for both proteins. The clustering patterns of known *Streptomyces* phages in both trees were supported by high bootstrap values (≥89% for major nodes), confirming the reliability of the analysis (Figure 5). These results indicated that CW39 has undergone substantial divergence in the evolution of its major capsid protein and terminase. This indicates that it represents a novel, independently evolving lineage among *Streptomyces* phages.

**Figure 6:**
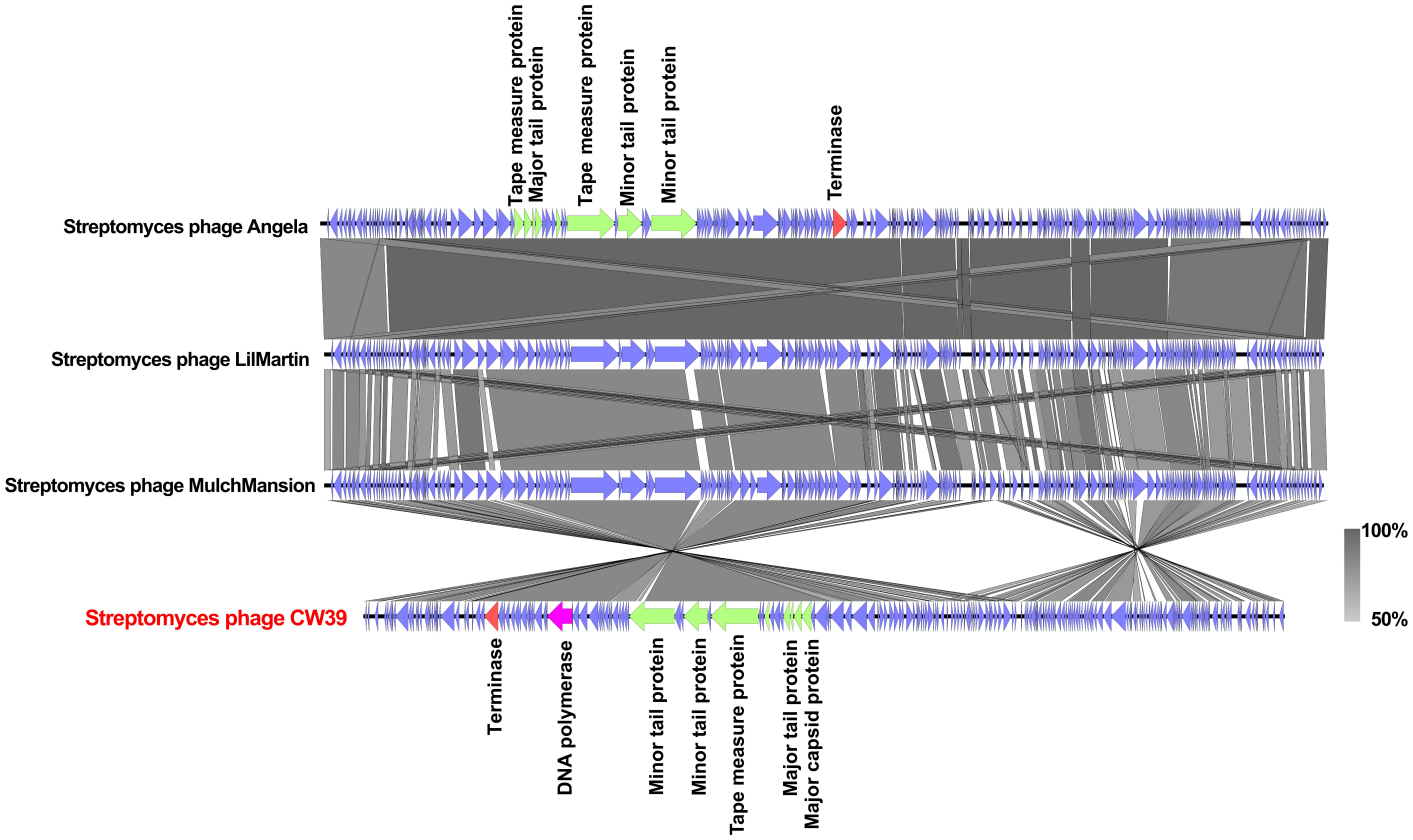
Genomic collinearity of phage CW39. Linear alignment of the CW39 genome with *Streptomyces* phages Angela, LilMartin, and MulchMansion was performed using Easyfig. Gray ribbons indicate regions of nucleotide similarity, with intensity corresponding to sequence identity.

Comparative genomic analysis against the NCBI database revealed that *Streptomyces* phage Angela (ON970591.1) was the closest relative to phage CW39, with 83.36% sequence identity, and that *Streptomyces* phage LilMartin (MT684590.1) showed the highest genome coverage (72%) (Supplementary Table 2). To further explore genomic structure evolution, the three most similar phages—Angela, LilMartin, and MulchMansion—were selected for collinearity analysis with CW39. Overall, CW39 shared extensive genome collinearity with these three phages. The arrangement of core functional modules, including DNA replication and structural protein assembly, was largely consistent, resulting in continuous homologous regions marked by dense gray lines. This high level of structural conservation indicates that genetic elements essential for core phage functions remain stable during long-term evolution. Notably, we also observed distinct local variations. In some genomic regions, homologous connections were disrupted, implying local gene rearrangements, inversions, insertions, or deletions over the course of evolution (Figure 6).

**Figure 7:**
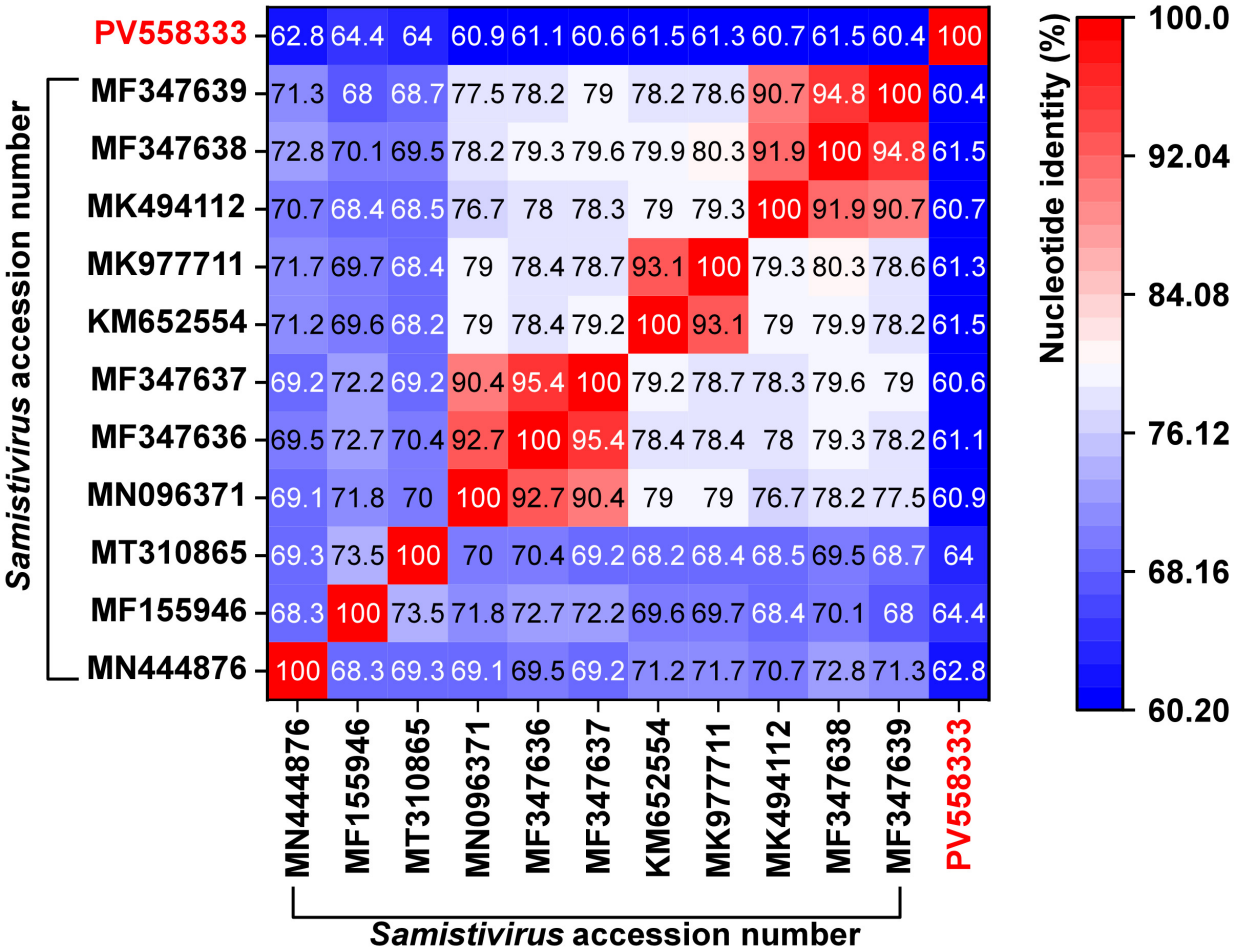
ANI analysis of phage CW39 and related phages. Heatmap showing ANI values between CW39 and related *Samistivirus* phages. ANI values were calculated by taxMyPhage. Red: higher identity. Blue: lower identity. Diagonal values represent 100% self-identity. Phage CW39 (PV558333) is highlighted in red to emphasize its distinct genomic position.

Average nucleotide identity (ANI) analysis showed that CW39 was most similar to phages within the genus *Samistivirus*, with ANI values ranging from 60.4% to 64.4%. The highest ANI value (64.4%) was observed between CW39 and *Samistivirus* MF155946, indicating a relatively close evolutionary relationship (Figure 7). However, this value is well below the 70% threshold for genus demarcation established by ICTV (Turner et al., 2023). These results suggest that CW39 is distinct from all known *Samistivirus* phages in terms of genome sequence similarity and likely represents a novel genus within the family.

### 3.7 Inhibition of phage CW39 by CRISPR-Cas9

**Figure 8:**
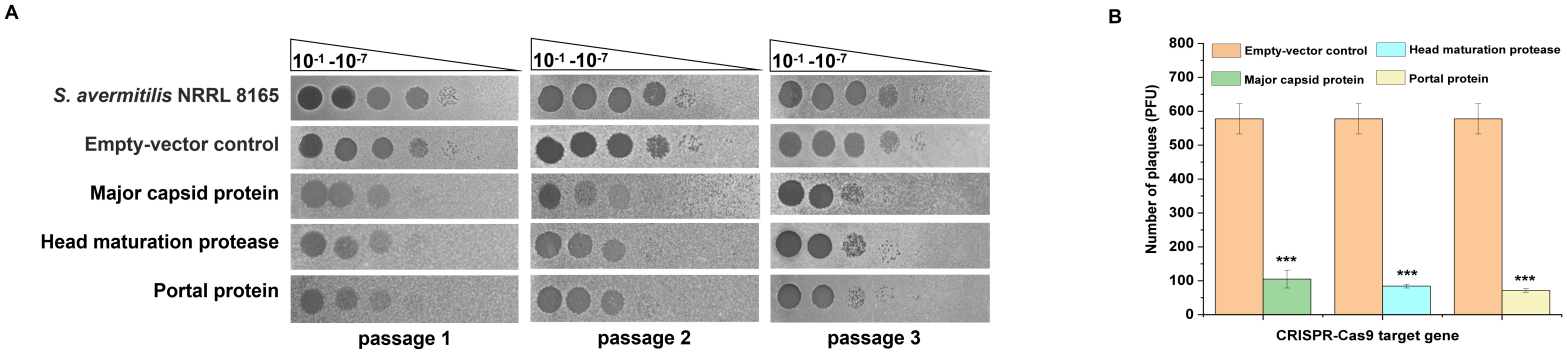
Inhibitory effect of CRISPR-Cas9 on phage CW39 infection. **(A)** Serial spot-dilution assays showing stable phage resistance of three CRISPR-engineered strains over three passages. Wild-type and empty-vector strains were negative controls; phage was 10-fold serially diluted (10^−1^−10^−7^). **(B)** Efficiency-of-plating (EOP) quantification of phage CW39 infectivity. Phage plaque counts of the empty-vector control and three strains with CRISPR-Cas9 targeting distinct phage-related genes. Data are presented as means ± standard deviations from three independent experiments. Statistical analysis was performed using one-way ANOVA followed by Tukey's multiple-comparison test. ***p < 0.001.

Based on the full-genome annotation of phage CW39, three genes were selected as CRISPR-Cas9 targeting candidates: major capsid protein, head maturation protease, and portal protein. Specific primers were designed for these genes (Supplementary Table 3), and PCR amplification produced bands of the expected sizes (996 bp, 1,269 bp, and 1,581 bp, respectively), confirming their presence in the CW39 genome (Supplementary Figure 2). Specific spacers targeting these genes were designed using the Benchling platform, with their corresponding PAM sequences identified (Table 1). After cloning each spacer into the CRISPR-Cas9 vector, the recombinant plasmids were introduced into *S. avermitilis* NRRL 8165 via conjugation. Following three rounds of serial passage, serial spot-dilution assays preliminarily verified stable phage resistance of the three CRISPR-engineered strains (Figure 8A). To precisely quantify resistance levels, efficiency-of-plating (EOP) assays were performed with direct PFU counting (Figure 8B, Supplementary Figure 3). Compared with the empty-vector control, the three recombinant strains exhibited significantly reduced phage plaque numbers (*p* < 0.001). The corresponding EOP values were calculated as 0.151 (major capsid protein), 0.141 (head maturation protease), and 0.114 (portal protein). These results suggest that targeting these core structural and packaging genes can effectively reduce phage CW39 infectivity in *S. avermitilis*.

**Table 1:** Core gene-targeting spacers and PAM sequences for phage CW39.

Gene	Spacer	PAM
Major capsid protein	ggccacgtaatcgaccacgg	AGG
Head maturation protease	aagtccgaggacaccgaagg	AGG
Portal protein	gcgctgtcggatacgcatgg	TGG

## 4 Discussion

As the model strain for industrial production of abamectin, *S. avermitilis* NRRL 8165 is highly susceptible to phage contamination (Lu et al., 2019), which severely compromises fermentation stability. Contamination often leads to complete fermentation failure, causing significant economic losses for manufacturers. Characterizing novel phages is crucial for unraveling host-phage interactions, which is pivotal for devising practical phage control strategies in industrial fermentation. However, specific phages infecting *S. avermitilis* NRRL 8165 remain scarce, limiting research on contamination mechanisms and the development of practical control measures (Górski et al., 2020).

In this work, we isolated a solid-dependent phage CW39 from soil, which infects *S. avermitilis* NRRL 8165 and *S. lividans* HXY6 at different efficiencies. Previous studies isolated phiSASD1 (Wang et al., 2010) and phiSAJS1 (Lu et al., 2019) from *S. avermitilis* ATCC31267 and performed one-step growth curves in liquid TSB to determine their latent periods and burst sizes. In contrast, CW39 infects the same *S. avermitilis* host but appears to rely predominantly on solid culture conditions for efficient infection. In liquid culture, *Streptomyces* forms compact mycelial pellets that may physically hinder phage access, whereas on solid medium, dense aerial hyphae potentially facilitate direct contact and infection (Flärdh and Buttner, 2009). Notably, CW39 has a long tail (~266 nm) and encodes seven putative minor tail proteins. In *Streptomyces* phages, minor tail proteins localize in the tail tip region, and their sequence variation likely contributes to host range diversity (Kronheim et al., 2023); receptor-binding domains are common in such tail tip proteins (Nobrega et al., 2018). We therefore hypothesize that these seven minor tail proteins may constitute the putative host-recognition apparatus of CW39, with receptor-binding motifs that may become functionally accessible only under solid-state conditions. These results expand our understanding of *Streptomyces* phage infection diversity and how the environment shapes phage-host interplay. Future work would be required to validate minor tail protein function and to expand strain testing in order to clarify CW39's host specificity and solid-dependent infection mechanism. Moreover, quantitative adsorption assays using isolated phages and *Streptomyces* mycelia at different developmental stages would be required to directly test how multicellular structures influence infection efficiency and host specificity.

Based on sequence prediction and annotation, CW39 putatively contains an integrase, suggesting that it may switch between lysogenic and lytic cycles (Groth and Calos, 2004). Given that phage integrases such as phiC31 integrase and its derivative vector pSET152 have been widely used for site-specific genome engineering in *Streptomyces* (Paranthaman and Dharmalingam, 2003), the integrase of CW39 could potentially serve as a tool for developing a novel integration system. Notably, CW39 also encodes a RecA-like recombinase and RuvC. RecA-like proteins may mediate DNA recombination and maintain genome integrity during phage replication, and could substitute for host RecA to regulate the lysis–lysogeny switch (Oppenheim et al., 2005). Phage-derived RuvC (such as bIL67 RuvC) can cleave Holliday junctions (Curtis et al., 2005), implying that CW39 RuvC might participate in processing replication intermediates and recombination repair. In contrast to temperate phage λ, which lacks RuvC and relies on the Red recombination system (Poteete et al., 2002), CW39 carries an integrase-RecA-like-RuvC module that may represent a distinct recombination and repair strategy. Future work should focus on how these genes regulate lysogenic induction and DNA recombination, and how these processes shape host-phage coevolution. All in all, the functional assignments here are based on sequence predictions and require experimental validation.

Genome and phylogenetic analysis indicated that CW39 shares some genomic collinearity with *Streptomyces* phages Angela, LilMartin, and MulchMansion, but CW39 exhibits low genome coverage, accompanied by substantial genomic rearrangements, inversions, and deletions. Phylogenetic trees resolved CW39 as a distinct basal monophyletic branch separate from all known *Streptomyces* phage lineages. Moreover, ANI values of 60.4–64.4% between CW39 and the closest Samistivirus relatives are far below the 70% ICTV genus demarcation threshold (Orellana, 2026), supporting CW39 as a novel genus within the class Caudoviricetes. This marked evolutionary uniqueness might reflect putative specialized host-adaptive mechanisms. We therefore manually screened all 208 predicted open reading frames (ORFs) and tentatively identified nine genes harboring canonical in-frame TTA leucine codons (Supplementary Table 4): two encode the HNH endonuclease, and the remaining seven encode hypothetical proteins. Because translation of TTA codons in *Streptomyces* strictly depends on the developmentally regulated *bldA* tRNA (Leskiw et al., 1993; Chater and Chandra, 2008), which is highly induced during aerial mycelium formation on solid medium, it is plausible that these *bldA*-associated genes contribute to the solid-dependent infection phenotype of CW39. However, further functional validation of these candidate genes is required to elucidate their potential roles in host adaptation and the evolutionary uniqueness of CW39.

Phage contamination threatens *Streptomyces* fermentation, and CRISPR has been used to engineer resistance. Qiu et al. (2016) employed the endogenous type I-E system of *S. avermitilis* to target phage phiSASD1. Compared with type I-E, heterologous type II CRISPR-Cas9 offers advantages for phage defense: single-subunit Cas9 is easier to express heterologously (Xu et al., 2021) and can be rapidly reprogrammed via sgRNA (Grigonyte et al., 2020). In this study, we employed an exogenous type II CRISPR-Cas9 system targeting the major capsid protein, head maturation protease, and portal protein of the newly isolated phage CW39. EOP quantification revealed significantly reduced infectivity for all three targets: 0.151 (major capsid protein), 0.141 (head maturation protease), and 0.114 (portal protein) (*p* < 0.001), demonstrating the feasibility of this strategy. However, complete immunity was not achieved with any single target. It is possible that mutations may arise in the target sites during phage replication, potentially reducing targeting efficiency (Sun et al., 2024). Notably, our design targeted only structural genes, which are more prone to mutation than early replication genes (Kupczok and Bollback, 2014). Targeting essential replication genes (e.g., DNA polymerase, helicase) may confer more robust CRISPR resistance (Garneau et al., 2010; Nasko et al., 2019). In conclusion, this study expands the range of effective CRISPR targets for controlling *S. avermitilis* phage infections. Future efforts should adopt multi-target combination strategies targeting diverse functionally important genes (e.g., structural and early replication genes) to prevent phage escape. Moreover, integrating the heterologous CRISPR-Cas9 system into the host genome, possibly in combination with the endogenous CRISPR machinery, may further enhance the genetic stability of resistant strains.

## 5 Conclusion

In this study, a novel solid-dependent phage, CW39, was isolated from soil using *S. avermitilis* NRRL 8165. Phenotypic characterization showed that CW39 tolerates a wide range of temperatures, pH levels, and organic solvents. Genomic analyses indicated that CW39 represents a putative new genus within the class Caudoviricetes. CRISPR-Cas9-mediated targeting of three structural assembly genes of phage CW39 effectively reduced phage infection in the host strain. Therefore, this study not only enriches the phage resource library of *S. avermitilis* but also provides a feasible strategy for phage control in industrial fermentation.

## Data Availability

The genome sequence and annotations for phage CW39 have been deposited in GenBank under accession number PV558333.
