# Supplementary material for: A novel putative genus phage CW39: implications for CRISPR-Cas9-based phage resistance in Streptomyces avermitilis
Source: Front Microbiol. 2026 Jun 26;17:1850649. doi: 10.3389/fmicb.2026.1850649 (PMC13350457; doi:10.3389/fmicb.2026.1850649)
Supplement: Supplementary file 1 [file Supplementary_file_1.docx]

**Supplementary Material**

**Supplementary Figures:**

**
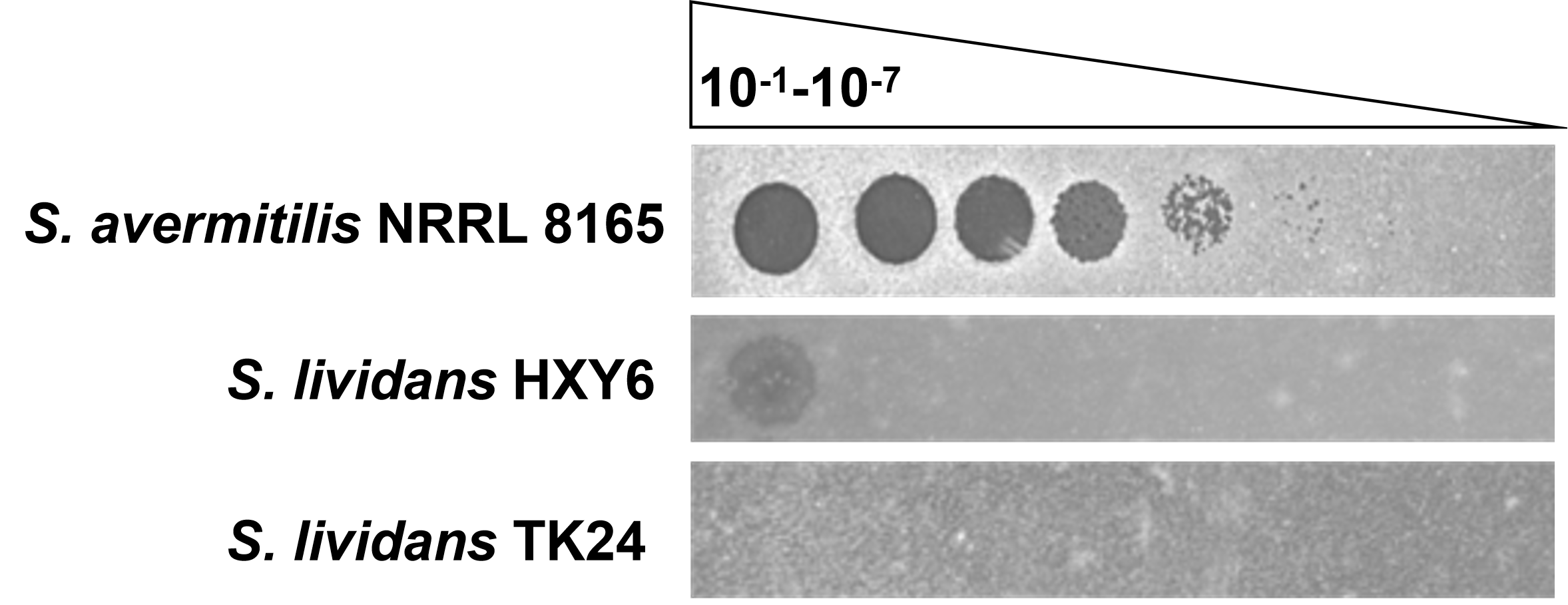
**

**Supplementary Figure 1.** Infection of *Streptomyces avermitilis* and *Streptomyces lividans* by phage CW39.

**
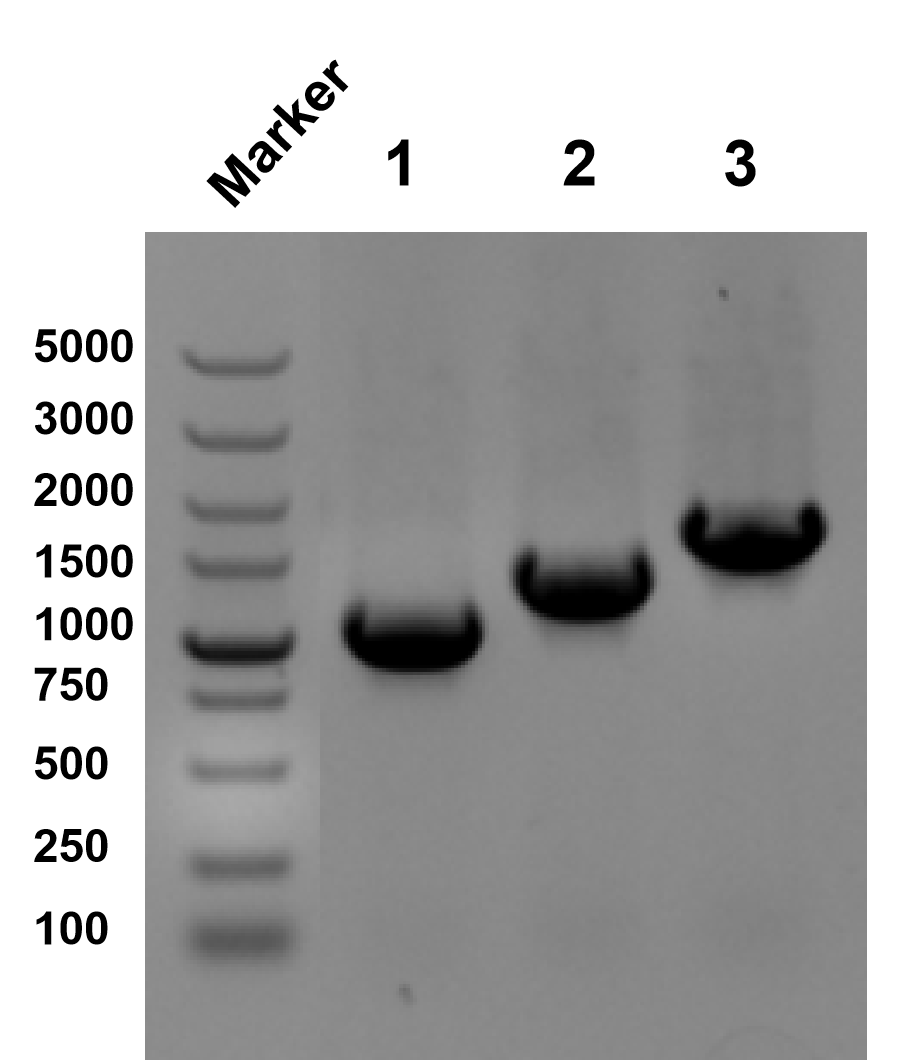
**

**Supplementary Figure 2.** PCR validation of core structural genes in phage CW39. Agarose gel electrophoresis analysis of PCR products amplified from phage CW39 genomic DNA. Specific primers targeting the major capsid protein (lane 1), head maturation protease (lane 2), and portal protein (lane 3) genes were used. A DNA molecular weight marker was included to estimate fragment sizes.


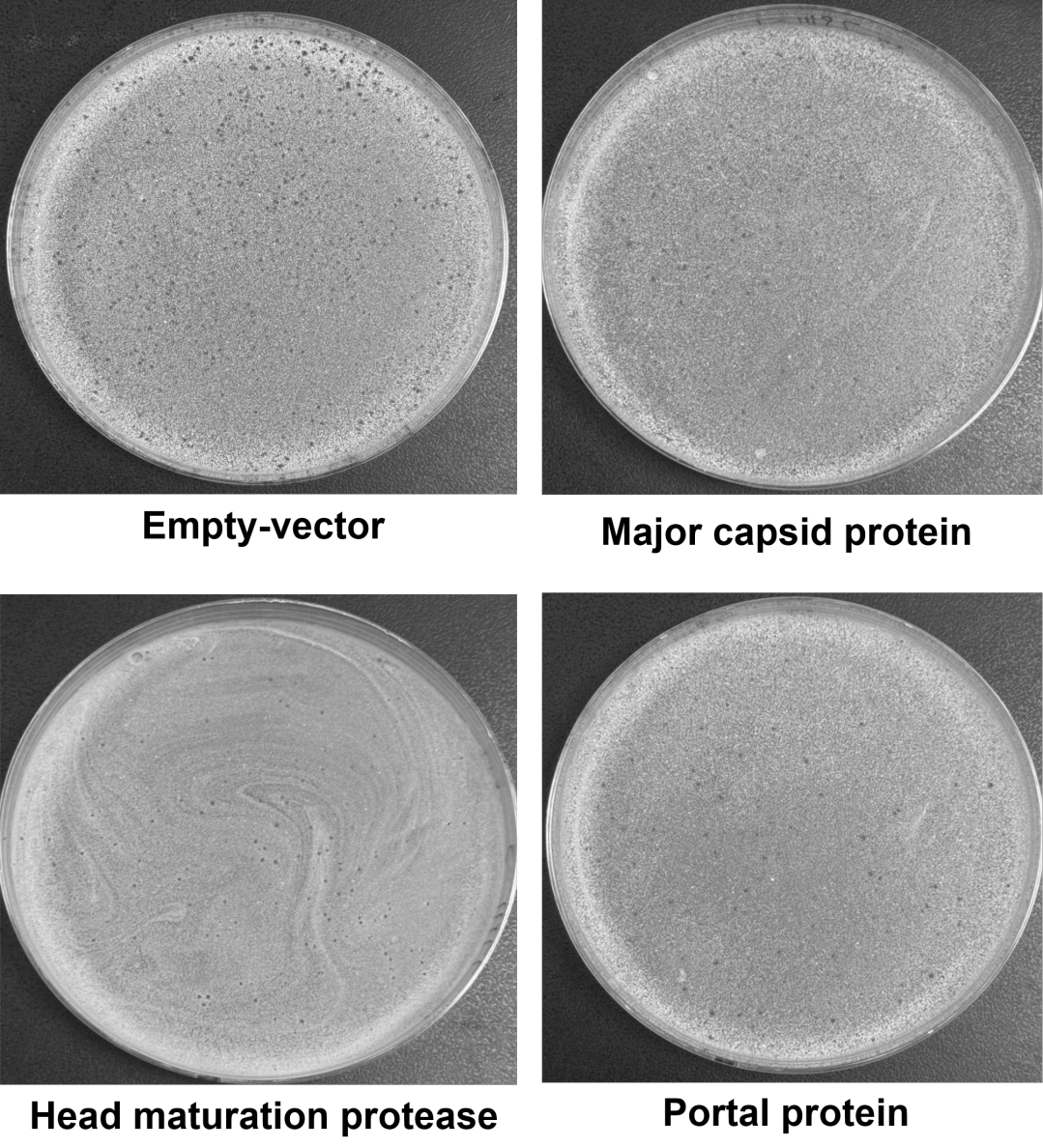


**Supplementary Figure 3**. Representative efficiency-of-plating (EOP) plaque-counting plates for phage CW39 infection assays. Plaque formation in the empty-vector control strain and three CRISPR-Cas9-engineered strains targeting major capsid protein, head maturation protease, and portal protein. Markedly reduced plaque numbers were observed in all CRISPR-targeted strains compared with the control.

**Supplementary Tables**

**Supplementary Table 1: Host range of phage CW39**

| **Bacteria** | **Plaque Formation** |
| --- | --- |
| *Streptomyces avermitilis* NRRL 8165 | + (10^8^ PFU/mL) |
| *Streptomyces lividans* TK24 | - |
| *Streptomyces lividans* HXY6 | + (10^4^ PFU/mL) |
| *Bacillus subtilis* 168 | - |
| *Bacillus thuringiensis* CTC | - |
| *Pseudomonas aeruginosa* PAO1 | - |
| *Pseudomonas fluorescens* Pf0-1 | - |
| *Escherichia coli* DH5a | - |
| *Escherichia coli* BL21 | - |
| *Escherichia coli* MG1655 | - |
| *Escherichia coli* DH10B | - |

**Note: "+" indicates plaque formation, "-" indicates no plaque formation**

**Supplementary Table 2: Phages with the highest genome similarity to phage CW39**

| **Phage** | **Max Score** | **Coverage**  **(%)** | **Identity**  **(%)** | **Length**  **(bp)** | **Accession** |
| --- | --- | --- | --- | --- | --- |
| *Streptomyces* phage Angela | 17073 | 70 | 83.36 | 133582 | ON970591.1 |
| *Streptomyces* phage LilMartin | 17062 | 72 | 83.35 | 134105 | MT684590.1 |
| *Streptomyces* phage MulchMansion | 17062 | 71 | 83.35 | 132470 | MT897905.1 |
| *Streptomyces* phage Teutsch | 16286 | 66 | 79.58 | 132885 | MK460248.1 |
| *Streptomyces* phage Mildred21 | 15817 | 71 | 82.23 | 131976 | NC 042008.1 |

**Supplementary Table 3: Primers for phage CW39 core gene PCR amplification**

| **Gene** | **Primer** | **Sequence** | **Length** |
| --- | --- | --- | --- |
| major capsid protein | CW39-76-F1 | 5′-ttaggctgcaatcttgacgtt-3′ | 996 bp |
|  | CW39-76-R1 | 5′-atgagcgatgagctaattgaaaagg-3′ |  |
| head maturation protease | CW39-77-F1 | 5′-ttacttgatgtcggaaacactgag-3′ | 1269 bp |
|  | CW39-77-R1 | 5′-atgaagattgagaaggcatcctg-3′ |  |
| portal protein | CW39-79-F1 | 5′-tcacggtgtagctctacctt-3′ | 1581 bp |
|  | CW39-79-R1 | 5′-atgacaagtaatcgagttgtagcg-3′ |  |

**Supplementary Table 4: ORFs containing TTA codons in the genome of phage CW39.**

| **NO.** | **ORF** | **Length (bp)** | **Start** | **End** | **Strand** | **Function** |
| --- | --- | --- | --- | --- | --- | --- |
| 1 | 7 | 390 | 3729 | 3340 | - | hypothetical protein |
| 2 | 28 | 135 | 14,442 | 14,576 | + | hypothetical protein |
| 3 | 30 | 537 | 16,026 | 15,490 | - | HNH endonuclease |
| 4 | 45 | 174 | 24,336 | 24,193 | - | hypothetical protein |
| 5 | 111 | 405 | 80,242 | 79,838 | - | hypothetical protein |
| 6 | 124 | 243 | 80,350 | 86,592 | + | hypothetical protein |
| 7 | 125 | 138 | 87,178 | 87,315 | + | hypothetical protein |
| 8 | 192 | 132 | 113,726 | 113,595 | - | hypothetical protein |
| 9 | 201 | 366 | 118,057 | 117,692 | - | HNH endonuclease |

**Note: Complete genome sequence of phage CW39 is presented below.**

gatgaacattaccagagccacaatcgctagaataccgacaatggtccagattacgtttccctctccaaacattcttgcacctccctgcgcaggaagtatgagaccgagtgaattccctaaacgcatatagcgtctttgaccaatggtacccagtctcatggtggacagtaggggactcgaacccctcctatttgcgtgcaaagcaaacgtgcttccactaacactaaccgcccaaagtccttattcaggactgtcaatcatattgaagtttttagcctctcgttccaagcaaccttcgcagattggcattggtacaaacatatcaggctcaacctctactgtcaaatcaccgtctgcttgttccgggcaaaatgtacacttcagaaaaaattcaactcccggcatggctgacagcatatcaaagttcatcgacaacttcaacctcgatgtcctttatgtctattccgcagatttcgccttcgccaaacgtatctctcactgcttcgagcgcatcagattggctcggggcgtcaacggatagcagaagacttactgcaatttcatatgtcctcatggacctattgtatcatctcgccgtttagttcggcttctctaagaaggtagtagacatcttggttgtactcgtacagagcgtcccagtctattgaaagaagtatgtcgccattctcttccatctcttcaatctgaagaatgcctgcgagaatcagctctgtcattactccattctctgagccagtttcgtcgttcacgtgccctccaaattttatttttaagcggagagtcctcgaacgtctggcctctccagacttttctaccgagcattttcataacgccctggacaggaatcgaacctgcctgtaactgtttagaagacagccgcgttatccactacgctaccagggcatgaaggtgaatatcaaggaatcgaaccttgcggcgggagctaccctatttgacccaatattttattacgcggactcgaaccgcacgtgaccatcacctaggctggagtcgaaccagcataccacccagtttattcaccagtcccgtagacgagaatcgaactcgcaacgcctccttggcaaggaggtatgttaccactacaccactacggattgtggaggttcctgttatgagccggaacaaccaagacgctctcaccttgtagctagatgccccttgcaaggccgttgagacaatgcggtttagagctgcgcatccccttgccgacttacattcccgcatctaccgcaggctagcggaacagccggattcgaaccgacaactgctactctgttatctactaccaggaacccctaggttcgacattgcatttcgctgttttgcccattaaactatgttcctcacactgggagcgacccagtgtgcatttgattaactccggatgttaatcaaagtaggcccggtgagactcgaactcacaatcctttcggcaccagttcctaagactggcgtgtataccattccaccacgagcccattacttacttctagaccatcctagcatacgtctggtccttctcgtatgacagttcgcacaacgaacttcacacttttttatttcgttttgcagagacttcattgagcacttcataaaatatgaaatccttggagccttacttccaaccatttctatgtggtcaaactcaagaacttcgatgtcagactctccgcaatctacgcatggattagactttaggtagtctatcacgtaagatttagtttcgtccagcaatttcttattggacttgcgagtcttgttagcgtagtattccttattggaagagtagtggttctttgcgtaatccctcatgcaagacttacaaataccatttcttccatcctttgctgtcttcttgagggcaaaatcctccaaagatttttcagttttacatgttgaacatctcttcataagattagaatatcacatgtaaattctaagtgcaagtacgccgtgggagagtcgaactcccaagcagttgattctaagtcaactaggtatgccagttcccgtcaacggcgcaagttaacgacgagtccggatttgaacccggcatggaggctggttagacctgcatgaaaaacaatttctactgagtgaccaacttttcgaaactctttactcgccagcgtggatttggagggattcgaaccctctgcggtcagcggtccattgtcggctttcactgacgtcataccaatcaaacccattaaactttgtacggccagtccggattcgcgcccggtacggagggtggttaagcctgcgtatgaaacaatctctactgagtgaccaacttttcgagactttcaactgacctgcgtacggacggtaggactcgaacctacgattggcacggtgtaaacgtgctgccttagccgctagacgacgcccgcttgtttgacatcacgcatgtggataaagtaggtatgctatgtcatctgaaagggttccttagtcccttgcagcaagtaccccgtgtcggattcgaaccgacgttcacacggattgaaaatccggtatcctagaccgctagaccaacggggcttgtagttactactctatgcggtgagacactgtgtgtcaagcaccccgaacgggatttgaacccgtgctttccagattgagagtctggcggattggaccggactatcctaccggggcatgtgacggacttaacgctccgcctgcgttgtgttactagtctagctgatgtatcgcagcctgtcaactcaaggcagcgggacgtctggaatctcttccggagactgaccaatgtcacgtccgagagtctgtggaggtatcgtaccatccgcagaacctgggatgtactgtcctaccttgttctgtccagcatcgaggaagatttctgcttctagtacatcataaggaaggtcaccaggaagagtttcatactctggtggctcaccgactagagaaaactgtacaacgatgttgtttgtctcagaattgtagtagaactgagaaattgtgtaataggccatgattcacactcctttcataatgattatatatgactaaccattatgaaaagctttggccttagttctgttctatggtgagagtgaagttcttctgattgccatcaccgtcatcgacgttgatatgcaggaactcaccatcacttgatgcaccgatgaacgcctcaataccgtcattccagtcaccatcaacatgaccagcaagtaggtctagtatacgttctgcatctacatgtcctagagacatcttgctccttacttgagaaacttatcgtaccaccggccgtctttcaggtcaagaggagacgccgtcaggaatcaggctctcgaacctggcaaggtcgtccttgtgaccatagaccgtaatgctgatatgcttgagcacccagccgcttgcactaaaactgtttactgtcaggtggcagctattggccaggtcacggatgtgagctgccatcttgccattgccagtacgagtgtaggacttggccttgtagtcgttcaccgttaatccaatcctgtcgaataggtctttgatgtatgggcacatatgccctttgttgacatcgtacttggtcttacaatggccacaggtagacattaccacatccaccggccgaattttatgacgtttgaacatgcgcagcagactgccgactgttacgccaggttcctcgctgttcatgagatgactctacaccttcgtgccgggcctgtcaacggatgaagttcacgtaaaccaggatgcatagggtggcggttgccatgaccatctggaacagggcagcgacggttcttaccagttctgcctttgaccttgctagcttgaggtcttgcctctcttctttaaatatcattactctatggtagcatacaggaaaacccctccgaagaggggccttctcagattgacttctggtaagctgacaaaatctctcgaatcttatcagctctacgctttcccatgtaaggaagcaattgcttcatcagctcagcagccttgacaccagagacctgggctctgtattggttcagcttgcttacgccattcttctgtagaggaacattgtaaacagcattccctatcaatgtggcggcacgctggattacatcgaggtccgtcattgtcacaacaatcttgggatataggtaaaccttacccctgacgtgtgacttgatggtcatgaagcttccttcaccctcaagcaatccagcaagccaggccaaatcgtttgacttcatcaaatccttccggcagagaagtctgctaggactcttggagcatttgagtcgaagcctacaaaatctagcatgaaatcgcaatccttgtcagcgatggtgaattcagttgcctgaactcccagtacagccatcctagcatcgattccagtagcctttcggtactgcttcaatgcctggaaaggatgtacatttcctgcccacgtatcattgtccgtgataacgacaaaggtgtcaacctctaccttctgttccatagcccaggtgattggcagggaacaatctgttccgccccagttgcttcgctgtacattacgcattgccgtagcaaggtctgtacgagcggagataccaaggtctgtcagaccgctgctcctgtatccccatccaccacttccggacgtgaatcctcgaatgatgtgggctggctcagtacgagcgatggtcattgccatggccgcagagacctgagcacagctaaggtcgagaccgttagcctgtgcactcatgctaccagagacgtcgatggctagcatcgtacgcttgttagctgggacaactgacttgaatgccttgtggaatccctcattgagggcgtccagaataacactctcagtcgtccagttcttcttacgaccggaccatgggtccttacggtcaatctgaccctctgcatagacaacagcagcattgaggaagttgattgggtgcagacgagtctgctcaatcagtgccccattggtgagctgtccagcataggacgcagcaaactgcatgtcctgaaaagcaccgatgcgtgcaagacgcgtgatgcttcggataagagcctgaccacgaagctgaccattggcaaagatgcgcttccagacactcaagtccttgtggaactgagttggaatcatctcccatgaaatcatctcgtacttgctcaggacgtcgtgaacatccttgacagtcttggcctgctgcacgagcttgaatccctcgatagcccgaaggtcatcgatagaagcgtgctccttgcccaggacaaaatcagcaacagatgtgttgacatcctttgggtgggaaagacgcattacgtctcggagcgtccagacctgctcaccgaacttacgggaacggtacttgaccgcctgataagccagcttgtcagcgtctcggtcaaaccaagaagcaataatctctcgcttggcgcgaccccatccggttccggaaagagtcatgtagttcagaagctcatacaggtgagtaccagtacgaacaaccttgttgaactccgccttgattgcctgcttgttcttgccttccatgatgagaatagcaagagcgtacagagctggagactgacggtatgcacgaccattgacagacacgtccacaatggtacgaagcacattcgcctcatcacgctcaatcatctcgcggataaagacgctgttacgtgaagcgtggtctccctcgtttacgtaataggttcctccatcgactccgagaatcaggaatcgctccagtcgagactggtcggaaacgctgaagacaaagcctccagcatcgttcttgacctggtcagctcgtgcagacttggtctgagaagtctttgcgttgctcttagcaacgttgttaagtgcgtttgacatttttgttaccggccctttctaaggcatattatggtacgtctggcacgttccctgacaggaagtccagctcgaattgttcgaagtcgtccctacgaatgttgaaatagattgcagcttcgatgagcttgtgtcgcttgttcagatattctagcgcactatcttcgattcgtacaaggtcgttgatgggatagcttgtcttctgagtgtgggcatttgtaggaccgggaacagtgtatcgctcagggtcttcactgggatactggaaaaggttcttattccagatgtacctgccgctaggaatccagtgccattgcacctttgccttgccggtactttcattgattgtatctacttggcctacacggaaagaagaactatctccctgccgcccgcctctgaagacaatggctcctggaacaatatcatgtccaagccagttcttcacagcttttctcctgacttccagtcgattgtagttacttctacaggttcaacggcatagacattatcatcgttgaactcgtcttcttgattctctgtcttgcctcgtttgtagcgtacaccaacgaagacgccttcgtcaatctcagcaacctcaagagtgcgaatcgcccatcgtgactcgccttcttcaacagtggctacctccttgggcttgaactcgtcttcagaaagattatcctcaatctctccaagagcgtcttctagacgccagccatcagaccagagctccttcagcttttcaattctttcatctgtattcataatggatgtggataaggtatgagagtcggagttgcccgaaccttgggcaatgtaggattcgaacctacctgtttctcatttcaaagggaagataaccgacttactcttcgacccacaaagtcccgacgaaaggaatcgaaccctcgacattgcgctgacaacgcagtatgttaccattacaccacgccgggatagccccgaagggcaattgataaaggtcagcgaatcctctttctgacctcttcccaggtagtttccactaccttgatgacagttagcttagggctgaacattcctttttcagcgcctgacttcacatactcctgataatcactttcagcaagtgccctcgccttggatggattgggagtggtgtaaaggtttgcgaagtcctctctctttccgtccttgagatactttccaaggacttcatacagtctatcggccacgagttccgtgccttcctgctgaacggaatatctctccttcatctggcaaagggaggttgtccatgatgggcttggttactgtgttgcaatgcttgcagttagaccgcaagggaagtgtcacaacgttctgaaaacatttcggacagtatctaattgccatgcgctccagacgggatttgaacccgcgatttcctgattgacaatcaggtgcattagaccagactatactactgaagcaaggatggcaagggaaggaactcacgtcgattccttgccatctacgttctaccttagcgtacctgctcgaccttgtcaactgactggactggaaccttcttaccagctactttgacatacttctctgctgccgggccttctacgaagactacacgagtgttaccgttgtgtccatggatggcttcaagagtggcaaggtcaggatacacagcaactgagttccactcagtcaaccatgtcatagcacactgaccgctgtcaaactgtacaccttgagcaactacgcctgttcctgagacaccagatgagtcgatgtctctatgaagttcaaagagtctcatatcctttaatcctttatatattctaatatattagaaccgattctaaatcgtattctatcttcttttcaatctttttctcttcttttttcaagtgtaactataccttagcagatgctcgaagactctgtcaactcctcccttgaacctcatatatctccttcttagtcatccctccttgcatcctgtctgtaagacattcttccgccttcacggccgccaggccgtcttctcgcaaaagaacaaacgagtcaggtggacagagccgtccgcctttttcctaattgctcaggacaccgctgggacatgtcactcggatgtcactgggacaaagtgacatgtcctctgaatgtcacgtgacagatgacagagtgtcctgggaatgacaaaagccatcctcagagaaagaggatggccagagtgttgatggtcacatgcatggtgttgtctgcaataatcatcaaccaggttgtcaaccatggtggagcgtcattgcgatatccagtgaggtttccctctgcccatgaataccgccattctttcggtgcgagctggttctttgcccagacaacatgtctcgccagtctgtaatggtcgatgacaatgtgcgtgacaatgatgacaaatagagctggtagtgacaaagtagcgaagacaaacgggacaccataggtgacaccgtggacaacggccggtagccattgctttgtcttgcactgagccatccagtctgtctggacaagataatcaccaatgaagtggagcagaactcctagaagaactgcactcacttcaattccttcttgagcctgctgatggtgtccttcattccattgacctcgaactgaaccttcagagcctcacggcccttcttgtccttatcgccacccttgagtccacgcatttcagccttcaactgagaaagctttgcttgcatggcaggcaggtctcgttcaagacctgcaaggaacctcttcttctcttccctgtccatttactttttcctctctggaggtagagttggtaggtatctttcgcaatcatagcagtaggctctcttccacccagaggctatgatttcgtcaccatgaatcgttcttacgttgacatggtaacagtccttctgcttcttgcctttgcctttcatcattctcctacaaagaagcccggcactatttgtgccgggcgaatgcggaaacggagggagtcgaacccccacgacatatcactacgtcatcacggttttcaagaccgataagcactccaatgcgcgcttccatggggaagtcagtctactacttccaggtcttcttcgttgaagccacccataacacccatgattggatgattgagttctctatcgaagaacataatctcgtacagtccagccccaaccttattgaggaatcgagtacgcttatgaatcctaccacgctttccgtagtattctggactcacgttgggacggtcgtgcttgacttgcactgtgtctccacagataatcatatcacacctcctgcggaaagcctgggagtcgaacccagccgagctttaacactcttagcacctttccaagatgcccgctagccaatcgcttactttccaaatgcctgacaagtcaggcgcggtacagttgaatctcatattccttgaacgttgccgtaccaaggccaccatgcatcactacaaggtagtcagtacggaaaatcttacgcacaagaaacggctcgattccagcaatgacgccagtaagtccagtatagcccttagcagactttatctgaatgacgtcgccttctttgtatttcaatctcgttccacctgcttcagtacgacgggaaagatttgaccttcacaaggacccatcttcatgagaaccttgacgagagcggaatttgattcaaccaaatcccacacttcaccttcacagccaaggacgtaaaccgtgtccccaactgccaggtctgccttactatccattacagcctccgagtattgtcgtcactaacacccataagaacagtagcgctgtcgagaacgacaaccttgttcttaccctgcatttgaacagtacccttcttgcccttgtggtcaccacgggtaatttctacccgagtaccggccggaacagaaagagccatagtttccccctatgggatttgtagttatagagtacgcctggatggaattgaaccatctatcgtccgcttataagacggatgcatcgaccgttatgctacaggcgctcggtccgaagacctttacgtacatcttactgcgaggcagagcacctgtcaaccctgtgagccggtgatgctctgcgctacgcttacgattccagtccaagactggtctgccacattgtatatcgtgtcgggcaaacccatgaatatcttggcaagaatcagtccacccacaataccccaaatgaggtcaggccactgacgacccttcttctttacaagatagagtgagactagtgcccagagtattactggaccgactagcaatgtctccatcacttctccaattgcttctctatgttcttcctgaagtaaccagccaccttgtacttggacccttcccagatacggctttctcgaccggtatcccccagaatctcatgcaactgacgagctgtcaagctgtggccgtaacccttgagttcagtgatgataatcttggccggtagccagtcatgttcatcaaagttctcaaagatgtccagcacatcttcgaacagtcttctgtccgtcgtgtctgtcactgtaacatctaggtaggtgtccttgtcaagctcgggcatgccctctgcttcacgttcatcacccatctctatagcctcatctggttcgatggggaagaccttgtacaagtagggttcccttgagccaccgcccaaaatgtagcacttaccagcgtcttccacgtcctcacctgatgctggatgaagcttatcaggtctccatccctgtgacagcatgccctctccgaacacgagaggtacgtcagcatgacgagaagcaaacataatcttgatagacactgcgtctgcgatagcagcgcccagagtatccttcgttgcctgctgagcagccaatacaacggtcacagcagccttacggccattgcggataatctggatggccaaatccttacacttcttattcaattgaatgaactcatcgataaagatgataagcgcaggatggtccttcgttggctgccaattgtcacccatgcccagcttcgtcagcttctttgcacgaatcttcgtgtactccagagcctcggtgagcatttcctcaatttcctctggggtcctgccacgtcgtgcaatggcactaccgaagacttcaaggccgttaccaccagggtcaatgtcccaggttacacagtctctacagctagtggtgatgtctgccagcttacgcatgaacattgacttaccaccaccaggagcagcaatgactactgcgtggttacgaagcaagctgagttcaagaggctgaccatccattcgctgagctaccttgtacttgttcttgattgaacgagacaaaggctctgctctgcgctctctttccatattggagaatgggtcagtctgcataagtctgatgacaacttcggcccggaatcgagttgcagcaaccaacataccgtcggcaggcagcctcaatagcgtttccatcttctttgccttagccattacatcatcaggagtaccctttctcaaaaccaccgggatttcccatccccaattgtatcgagtaggttccaagattactctaggagtgatgccctctgcaagcaatgccctgcgtagagcttcctgagcctcgtctctcgttcgagcgtctgcaataggaaaaggttccatggggtcatcggacccgtcatcatttcctgtaatgccagcgacgatgtcagcaggcttgatgccacttcctatgaaccacatcactgtaaagatggcacccagggctccagccataggcaagattccccaagtagcgtaactccatcctgctacagctatcaagactgcaaggactgagtagaacctcttacgtctaggctctgtacgcttgtcctgttctatcttcagcttccgggccttttcgagattaccgtccagacgtgccttggatatctcttccttatagtcattcagatagacccaatccaaaatggctttactcgttattctaatacccttccggacatgcaaaaatacaccctgtccggaagaccgaaaagacctggtgtcaaccaggtctgcgtcagggcgtttaacaagttccgtggagtgcttcttatcgaaatctaccatagtatttgttttctaggatgtctcaccattgagaccaagttggggaggtgggatttgaacccacgaccgttccgttatcagcggaatgctctaaccagaccgagctacaccccaatggagcgggcctaaacccacttttgaacttcatcgaggacacctaggaagacatcctcatatgctccagtctctcccatggatgagcttataatgaacttatccatgctggaaagcctgggattgaacttaccgtattccaaagctgctcccagattgagaagcaagcggatagcatgacgcctaggctttacttcatccttctgagcgaagttcagagcggttcgaatatatacttccttcatcttggcgcgattcactcgatattgtgtccggaaggcgttgatttcatcaacgtccgcaaccggcgtccatagcgcttcaagtgcttgaggtacacattcgtcaaccatgtgcatgaatgttgacaggtctacgaccgttttgtcaacgccattcacgatggtttgcttgatgtttcgcttacgcgcagtccgcttagtctggacaacttcgtagatgtccttgtcagaccctgcatgagcgaggccatatagatgagagccgtggactgtcctaagaagtaccgtcatatgtaaatcctttaggtatgtggtgagaacggagagtgtattgttccaatgttaccttaccatacacatctcaggtaactgctctagcgagtttttacgtcgcatttccattgaatgataaccgttaactctatcgacccaaattgtcgggtaccggagactcgaactccgcctaaaacatcccaaatgtctcgtgcttccccatacactagtacccgaaattgcatctccatgttccaaactgttcctgagttctgattctgtgacagttagcgcatctcacgacacacttttcaatctccgtctgaatcttagcccatcccagcatgaacatttctgatacattgtaagactttgttgcttcatctttgtggtcaaattctagaaccctagggtcagaattaccacagtcaacgcatgggttcgttgacagataagcccaaagcttttctttattggcttggattgactttgctcggtttctctctttgcgctccctgtcaccattccgatatcgctctcgctcatattcagcgaagcatggcctgcaccatactgcgagaccatctggtcttttggagttctttcgaaactctgtaagttctttatccttaccacatctactacatgtcttcatagacttattgtagatggtttggggttatgttgcaactcccaaactaagtcaggcccctcggaatcgaaccgagtttcttaggcttccaaagccaacggattgccatcttccccgagcctgatagcagcatctctgctgcgttgtacttaatcctagcagacgcttcggagtgtgtcaactctgtgtcacgttccttttggtacgtccttgtaatactttacatcactgctttcgtagctgtcaagctctgcttgtagagcatccagcttctccctaagctctgccttccttgagtctacgataccacgctccaaactcttgtcaagagggagggcagttacgaagtcaggctcaggataagtaacgatagcatctgcctctacgtgccgcccgtttctgaaccagtgcctgcccatcttaccattgtctgtgcggtatgtcacgatgaagagtacgttgggccactctcgtgaaagaatcttcatgtcatctttcatgtcgtaccacttcatgtttcgagtgtagttcatgatttcgccgactgtcaaggcatacgctccaggcatcttaaccgtatccttggtctccatcagcttattgtagattggaccgctgccttcaacgtccatttcccagtctgaataagtccccacgtgacctccaatagctatgcccctaccgaagtaggggcttttgttaaatggttttgacgctgtgcgggaccttaacccctactacctaaaacggatagtctcagcatgaccgtactccataggactatctctcggacgttataagcagtttatgctgaccactgccgttgccgcctgctaaggtgagattcgtcaaagcacccccaccaggagtcgaacctgggcttttggttttggagaccaacgtgctaccgtaacacttcggggataaacagttgcttgggcgtgaccccttttacttttcagaatgtgtctcgtaggcgagtctccagggtatcacacattcatcaagtacagcatgcaacttatgctaagttgtcggtccgaggaggttttacccatcgcttcccttcggaatcctatcatctttccacgcctttaccatggttattatgcgtgttccgaatcaaatcctcttccagatatcacgaagtcggaccagcttccccggcaggagtcgaacctacgttactgaagttcagagcctcagtggccaaccacacagaccacggggaaataagtgggagataatcattgccctctccctgttaggtactacctaacggacgccattcgtgcgctgccagacgccaacggccttccctctgctaatgctggcacttcaagggactagtaagagcaccgagcgggaatcgaacccgcaattctgacttggaaggacagcgtgttaccactacaccaccgatgcaatagtgtggataaggtgtgagaatcgggtgcccatttaagagcttctatttgagtggttagataaccgatttactcttcgacccacatttaatactttatcagctcactcgctgtctgtcaagcctgtagcgttctgacggtcttcgtcagtgaactcgtaggcaagacctgagcgactggcctctacatagcttgtcacgctcctggacatgttgtcaagtccgaccttgtcagcagcaaaggtaagactggaagccttggggattcccatcttaccacctactgagatggcgtcttgattcgcgcccagatagacaaactcgtcaccgtgctgtcgtgactcttcgattgctgccttcactgtagcattggtccactcagttgaacagttctccaggccatcagtcatgatgacgtagattgtcttcttcgtctgtggaacatagtttactacagtgcgaccgatggcgtcaagcagggcagttgagccacgaggacttaccttgacctgcttgacatccttcagagcaaccttttcgaatacaagctcgtattcgtggtcaaaccgcacgagagtgacgaacgcctctccctctaccttcttctggtcatcgaggaagctgttgatagcaccttcggcatctgacttgataccccacatgcttccggaattatcaacaatcaatacaatgtctacgtcagtcattactctcctttttaggagtgccgaggactagtctatccccggctctcctccatcaaacactatcctactggcaggcaccctgtcaaccacaccgctcatacagcaccagcaccaccagatgccttcgtgtgtacagtcgtttggcttcatcttagcccactgagggtaacgaggtggcatcggaccgctaccacattggtcgcactcctcatcaggtccgagcatcctaccagactgacagcgttcacaaacttctggacgctcactccgcttgatagctcggctgattcgttccttctttggcatcacaggaagtgttccatcagcaaaaggcattctgtcgcccttggctgaattacacttcttgtgcattagcttcaggttatcaaggtcccatgtacctccaccgctcaaaggaatccagtggtcgatggtaggtccgttatccttcacgaacggaaggtcacagcctgggaactgacacttgtagccatcacgctctgcaagcagttgagaaatctcagctcgtgagattccctttggcttacgttgtaaattaggcatggcatacctccggtcatagaatttctagcctagtcaggtagtcagcgatgtcatctggcatcactgcaccctctctaggtgcttgaatcacattatcacgttctcgcatgctgtcaagtgcctcttgtcgagccactgcttcagcacgtctgatagaatcgtaagtctgtacctcaatggtaccaaagtcatctcgtggcgtgtgagtgattgcgttgtgaactgcaccacacgttgcgtctgccaagtcctttgaccccgttctagggtggtcaattttgtcgtttggcatgatacgcaactgcaacagttctttcctgagcaattggatgtcaggacccatgattctctggtcatagattacccagctcaagtcttcgtaatgcttctttgctactgacaatacctctgaattgattccgacaccacgaaggtacttcatctggtcattggactgccagcggtcaaaggttactagcttgaggtcgaaacctcttcgcttcaatcctgtaatatactctctcacatcggcaaagtcaacatccttgccgggctttggtgtccaccaacggacagcatccacgattacataaggaagcacctcattaagctgaccaccaatctttttctgtacaaacttttcaacatgcgcaagtgctacagcacagtggtcgtgcttctgcgcaaggtctacgtgtacatagtagcgcacgccttccttcggtgtaaaatctgaccgatatgcaccctcgtcgtcaacaccgttaggtctgacaaagacagcttctaccttctctttgtccttgaagaaagcgtcaatggcgtcaggtggcatacaggcaaatcgagaaagtgcatcaatagggtcagtaaagaatgcagtggtaaagtcctgaattgttctggttgggttgacttcccaggtaggacgcttcaaggcaaagacacgaggcacattgtaggaaagaatgtggtcttcttcccaccggattgaaaacttgttggattcaacatcgtcaggaaggtcagggtcaagcttgaaagtatgagtacgctctacgacttccttctctgcaacaacggcctcatatctctgagagataaagtcgttcttgaagcgagggaatgacagaaggactagcttgccttctgtagggaatcgagagtcaacagatgctctatacatcttatagactgcgtcagccgtcttggcctgctcgttacctgatgtagagtctagtgcgaaaccagaaatctcgtcaagcacacagtagataaggttgtagccctcccaagcctctcgctctgagtggcctgagtagacgttgatgtttttgtcgaaagcaatgtgaccagccttcgtggtgaactttcctgcaaaccatggtgactcttcgatgcggctcttgaagcccttgaagaagacattgttggcctgtgctgcgttaacagcaatgttcaggatgtcgatggagtcgcctgatggctttccgaaatacttcgcagggttacgtagacacaggagcaaataaacaatatagctacaggcgatggtggacgtgaagtccttaccgctacccttgccaagacatgcaatgacttcgttgcatgtctctttccatcttcgattcgccttctctgcgtcgtacagattatgcaatgtgctctgcttataaatttggctgcttgctcgaataagctggtactggtactcagacaaatgcggcatgccaagatagtcttcgctctgtacgaagacctcaatatccaccgggatttcttcaaaatcatcatcagacaatgcattgaagaactcagcgaaatcactagacaaaactaaatcccttcattgatatttagtatatcatgaagggatttaatcacttgacgacaatcgtctcagttttgtctgtcactctactaagtctccgtgctacttcaaccttacagtgaggacactctgaggtcacctcgcgcagaatggtaataagaatctcatgcttgcgttccatctcaatgacttcatcaccgatttgctggttgtccagcagaccagccttctgcaaaaggtcaacacgcttaccttctacatcagcaagactcttcaagactgctgtcttggtcttgaggtcattgttcatatcagcttcctctaggactccccagagttctctgataatcatgctgtaatgctcatccatgcctgtcaaggcttcctgagcacgcgcctgaattgtcttattgttctgcgccagcgccttccactcgtctatatattcgagtacctgagcacgcttcagccctgtattccgggcaatggcggttgcattgtcgccctttagatgtgccgctacgactgaattgagttgttcaatatggtctagcgtatcaagctcgctgggcaactgctctccttctacgtggacgccttggctttaccagtccctttagcttctccacatagaatgacttccactcacctgtgtctgcattgatgcagtcaatccaggtcttgccagtcttcgtattggttgccacacatcggaacttgaatgagcctcgcgtattcttgaacttgatgagtgacttggggagaactgaatctccgtcaatctcaaattcgtatgaagcgatgatgtgcgggattccggcatatgctccttgccaccaggagtctggattcttttctcctcgtcggcgtctagtcggtgttcccattgtatctcctcactttcaagataattatagcatgccctggaatacttccatccagcgctgggcattttctctgtctgctgtgtacagcttctgtaccagccattcagccctttgctcgggagggacattgggaaccctgtaatagtttatatctagaagccccaggattgtctgaatgttgttgtcgatttctcgacggtactgctcgctcgggtctcgtactccgtcatcaacattgtcccagtagattgggaagtacagcagaatgttgtacgtgttgatatgcatcttggcaagacggaatgagatgttctctaccaggtctccgctttcccacacatgcttattctggtacatggtgtatgcaagggagtcgaccagtgtacggtctgaaatgattccttgcttgtacgccctgattgtgtggtcaacgaatgcctctgcctcatccaccatgcgtagaagaggtacgagaatctgacttagttcggtggcttctctattgataggatagccatatgcttttatctgccgggccgttgatggaaccagggaaaacgtaccgaatggaccattgaccattgcctcggccattgaggtcttccctgttccatgtgcacccaccatgccaattttcatcatagacgacagcatagcacacgtcaacggctacgtctacgcctgatgccgaacttctcaagataacgagtgattgtcatctctgtcacgttgcaaagagctgcaatctgtgctgcgttcattgtcctgtacttggactgtagccaatccttactctgatacagcttctggccagacatcccacacctccctcatcaaagggtcgatgatggtcataggcttcttgacgcgctcggtgtacttcaggcaattgtaagttccacctgttctaccggcctccaatactcccagaacacaatcagccttgtcaaccatgtagcgattgcgctcctcgtaaatccatgcaccaggatagttatcgtaatcagttacgtcaaccacctcatgcgcgtactggatagctcgcgtgtaatcgtagacgtccgcgttacgtggcctgtggcccttccatggctttgtagcaataaatgggataccaagaccccatgctgttttagccaatagaaggtcgaagcctgacgccatgcctacataaaccaaagaggcccccaaatcaatgagggccttctctgtctgcgcagcaatccagtcacggtccctcaaatctctatgacctgttcccgcaacaatcattgcttaactccttcgtgttttattctgcactgcccaccaggcgatgcccagtgaatcagctacgttgtcactttctagcttaacgccaaactctcgctcagcaaagtctatggttcgctgcttccttatcagcctgcccttattctgataccagcttgcagactttccaggaaattccttcttgatagcatttttctcgatgagtgtgagattcttgttgccaatgtatgactgccatgaaataggcggtacttcaattactatcgctccctttttcaagagagcagcaatcacggcaccaaacacataggccatcttaattgcgactgatgtgcttcgaaccataatggctgcctcaattgcaacaaaatcaggagagaaactttcgaacagtgccaaagtctttacgttcgcatcatgcactctttcgaaaactgtcgctccatgaaagaaaacctctccacacttgaggaacttttcgtcctcaaagagtgtaaatgctatggacttggttgagcagtcgattcccatgactctctgagcctttgtcttatgtatatcagacagagacactgtcaatcaactccaatagctgtacacgttccttccggcgctgctccgctttacagccctcacatctgtctgagctgttgtatctggagagcaccgttgggcaccctgagcactttctgacaattcccttttgtcgtgctcgacgggcgtaatacttctccatgattcttgcgttggtcgccttacggcaacatgctgagctgcaaaacttctgattatgagtcttgggctcaaacatgttaccgcattctccgtaggcgcaaatcaattacttcttcacctccaatggtgctttagtaatgtcgccatcaggagattcgttccaacacacattgaatagcgcgcatgacttgcacgccttgttcttctgagtaaatggtcgggtcggcagggtcttgttttcccaaagctctctggtctcacgcatccactggaaaagatagtcagtgtattctgaattgcgctcgtccatcgtcagtgggacaaggaatagttcctgagtgttcttgttctcgtagagcacgaatccgtcgtcaatgccgaggactcgcatgtagataagcacctgcaccttgtgataatcaatgggcgtaccatttgcctcacgatggatataggattcctgtcgggttgtcttgaactcaccgacaatcttcttaccttcaaaatctacgataacgtcaacgaatcccctgattgggggattctggtaggtgatttccttttctgccacgtcaagcatacctgcatcgccaaatagcttttcaatacgtgtatgcgctgctgttccgtttgccatattggcgacactctgtccgtcaaaggtgtctacaaactttccgccctcaaaagccagataccagtacctaggacaggtaccggcaccatagccaagagaggacggactgaatgaaaccttcttggaatatctctcagtctgacgagagccgacatatgcagcttcgagtgtagctagaagcttttttgtgtcgaagtttcctcgcttccccagactggtaattcgctttagtccatcagacatgttttgtggtttccgtttctcttagtgattatcattgtatcacatcagtaaggcagtttgaaagtgtacttcaatgcctgaaccaacttgtcgatagactcagccgtcgtgtaatagatattctttttgtagttgttcggactgcccgcaggagccttcttgattgtgctgtagattgacgcaagcatagcgaactttgcagcatatgcctgtagctgaacaatcagtcgttggtttgtttctggcttcagatttggattgaccatgagctggaccataatggcaagggcctcatccagttccgggtctgccatgaaatcatgaaggtcttgaaactcaccaatcttggaaatatgttccaatggggtgagttcactcatcgtacatctctgcaatcattgcttcgacttcctcacgggtgacaggaccagcaatctcgcttccgtcatcgtacttcttgtaagcaagccatccaccagttccccgacgaatgtattcagtcctgactagctgagccatacttttcctcccatgcttccagcatttccttgaacatgtcgtctcctattacccagagacgtacctttcgattaccggcccccaagactagcttgaaggctggaatcattctaccagaccgaaacgcgtctgtacaaacctttccccagttcgtgatactcacgctgaaggaatttgcgtattccttgatgtcgtagcagaagggtcctagtgtagcatcacccttctgaatctgtccacgacctgagttcttctgaggcttgccaccatctctcttgatttcttctagctcactcatcagactcctaggttgaagttctctattgcagacttatgccctgtgatgcatatccatacaagcatcttatccacagggaagaattctgcttcgtcaacgatgtcctcacacgtcatgcatgcgaaggctccatcgattgggacgcctctagggacgtcctcaaccggcttctttggctcctccaggaatggatttttcatcggctctctacttccttcaccagcttttctgccaattctggacgctgtctgagctgaatggcgaaaccatccttaccgttgaccttgaggtcttcgtatgtcagccagtttccagacttgttgacaatgccaagagcaacgccaaggtctacagcctcaccgacattatcgattccgatgaagtcaccatcgtagaacagattgtaatcaccgtatcggttgggtggacctagcttgttcttgtccacactccagatgaccttacgaccgattgactgcttgagtacaatgtcaccattgaaaacttcacccttcacctggtcatcatccttggcagaactccatagctttactacggtgctactgaagaagtctaccgcatgaccaccggtcgcctgatgcatggctccataggtcgtaatcttgttacgtacctgagagatgagaatcagagcagtattcttgttggcgtagttcaacatctttacgccattagccagctcacgtgcttcggaaccaatctgcttagtgccagagaggtcttttagttcttcttccttgttcttattcttgtccttctcgaagtaagctgaagaaagaagcgcagaaattgaatctgctgtaatgatgtcgacaccggccctcataaactcaacgcctgcgccaaccatgtcttcgattgtctttacgtctgtgtagataagcttctttgtatctactcctagacgttctgcccaagcagggtcaaaggactgttctgcgtcaatgaatgccgctctcttaccggccctttgtgccaacgccaatgttcctaggcagaatgttgactttcctgctgacttgcttccccaaatcaatgtctgtcgtccatagccgattcctccacgcagtgcagcattaaggccagtgctaggcgttggctgcttcatcaattgcacttcggatgccatcttggctcttgctaggactttggggcttagcttagctagaatctcctcttctatttccatattctctctggttccattcctcttcaagttttttcgccattgcctttatttttttatcgcggcaggcggccaaccgcgcaatgataaacagaatttcctccgcgtcatcgcctcggaataccaataggtgttcgtcttcgattccacggaggaaatatgcttctgtgctcatgctctattctaccacttagcgggtgtcgccgctaccaccgattacgcctcgggcagcacgtgacttgagcttctgtagattctgctttgcaacaagctcaagaggatatccaagctcacttgcaaggttggcaacgtaccaaagaacgtctccaagttctgctgccagcgcctgcgccttttcagcagttacaactccgccatcgtcacggattgacttcttgaatccgttggcaatctctccagcctcaccaacaaggccgaggatggtgtatccgatggcagccgtggtggcttcaccgttctgaggatagattgcagtttcagccgcagtcatctggtagtcggccattgaaaggtcgttcacactgtctccagttcaattatgtattcgtctaatgcatcgtcgtactttacagcgatgttatattctgtctcatcaccaagagccctgtccatattctccgcagagacgtggacagggccgttagcaatgagtaccaacatcagaatttctgatgctgttactgtacccatttctctccttacaggatgttgtcgtaaaaccagctaccctcttgcgtctgtttcaggatagcatcaatgactgcaccgtccttgcacttaccatatgctttgtggaacatggtagggaaggccatgacggcatacatgttcttttcagaatcacatactgtcgtgtaagccattgtcttctttgccttggtgatgtacctctggaaagagagcaccttgtacattccttccggaatgtcaccgtactcactcgcataaaggtaatcaacgaagccgtttggaatcctgttgattagttcatccatagttgcaaagcgtgcgaccctgttgtcagacaccaggatagcatacatctgaccagactcgataggagtctcaggattggtaaagacaccagcagtgccagtctcatctaccaggtccaaacgcgcccatccctcaccgcgcttgattcccctgaccatggcaagcacacagaaagcacccttgtccttgtagtcttcaaggtcagtaaactgagccctgacctttgggggaatgaacttcagctcgaaggcagggatacgaaggtactcgtagaagttctctcgttcctctcctgtacgaggcatgtcctcgaatgctgctgcacctacaagatacagagccttcaaagcacgtacgttcaggccgttaccagactcctgaatcttttcctttaggtcagcataagaggtgaatggacgatactcaatcagcctcattgcaccaggctcacgaatgaacttgatgttcgtcagaccgaaacgaatggcatcaccctcaatttcgaagttgactccagagacattgacgtgagggagaagcatactgattcccatacgcttagcctcaatcaggtactcggtaagagcgtccttgtctccttcgttgcgaagcgtagcacacatgaactcaagagggtagtgacgcttcagccacgcagtgtagtaactcagacgagaatacgcaattgcgtgagacttgttgaaggagtagccagcgtgagcctcgaagtcgtgccacagcttttcagctacagcaggatgtaccttccttgaagccccttccatgaattcggccttgtactggtcgaactcacttgcatccttcttcttaccgataatcttacgtaccttgtcagcagtggccatcttcattccggccaattctgtcatggtaagcatgacctgctcctgatacagaatctcaccataagtctctgaggtgaagttcttcatcccaaagtggtggtacttgacaggagacttaccgttctttcgagcgatatactccttaccaatggtgttcattgcaccaggacgaacaagagcgttagaagctgcaagctctgcgaaattcttcacgccacccatattgataatgagctttgtgtaagcctgcgcttcacactggaagacacccttcgtgtatccatcagaaatcatggagtacacacggctgtcagtcagaggaatcttgttcaggtcaatgtcagtaccatggcggtccttaatcatggcgagtgcatccctgataatgctcagagtcttgaggccaagggcgtccagcttcaccagaccgaggtctgctgcctggtccatgtccatggctacaagagggatgcgagggccgtcagggtcgttggggtcctttgcggtttcaatgggtgcgaagttggccagaggctgattcgcaattaccacaccggccgcgtgcatgcctgtctgacggatacgaccacgaagacgcttaccaagcttgatgacctctggatacttctttcgaaagtcctcagtcgtaggtgactcaaggaattgctcccaggtgtcgacatacttcagggctcggttcacttctccaacaggaatgcagaacacacgagatgcgtccttgatgactaccttgtccttgaagtaaccgaatgttgcaatggatgcaacatagccatacttctttcgaaggtattccttgacctctccacgacgaacatcttcgaagtcggtatcgatgtcagggaagtcattacgctcagggttgatgaatcggaagaacagaaggttatccacaatagggtcaacctcagtgattccaagagcatagttgacaagggagccagcaccagaaccacggccaggaccgaccatgattccttcagcctttgcccagctaatcatgtcttcgacaatgaggaagtaagtagagaagtcctttgccttgatgattccaagttcttcttcaaggcggtctacgtacgtcttgtcattggcaaagccgcgactctccaacccgcgccgggccttaatctccagaaggtcatcaggattaccttctttcggacgaggcagcaggtcaagtccacgatggaaaggatagtcaccaatcatgtcagcaacaacatgcgtattggtgacaatgtcgtcacggtcaaatccctgagccttgaacgcgttaaggtgctcctcggcagagtgaagataaatctcaatcttctcgaaggtcatcttacggtcagggtacaggtaattgaatcgcgcaagcatctccatcttctgtgacttggagaattcggcgtccttgttcttcttaggactcgtagacaggatgagcattgcttcctgaatccacaggtcttccttacgagcatagtggcagtcagacgtgacgacaggaaggattcccttttcgtcagcaatcttgaacagcgcctcgttcatgctcttagggttgtgaccctgaacctcgatgaagaatctgtcgccaaggatacccttgaatcggtcggcaattgccatagcatcatcaagctcgcctcgctcgattgccttacagataagtccgttcaggcatccagaaagaacaatcagaccctcattgtcatcctcaagaacctccatgtcgatacgaggctttgagtagaatccttcggtccaaccaatacggttgattcggttgagagtctgtagaccagtttcgttctgagccagaacgattaggtggttatatgcatttgtaccatcggaacgtgacgcctttgagcgccggtcgaatcggtcggtaggagagatgtaagcctctacaccgagaattggaacaatacctgcgtccattgcagcttgctgaaactcgcggtgaccgccaagggtaccgtgattcgtctgggcaagatgcgtcattcccagttccttggcgcgaaccatatactcagcagcagtattggttccgtccatggaggagtaatggtcgtgaaggtggagttcaacattctgagtcattgaaaccttccttgttgttgtaggtccactctagcagaccagcctctgacatgcaagaagccccgccgaagcggggccatcttgtcagtcttattaccacttggcattcaggtcgtaaccagcacctgaactctgtgatgccgcagcaggagctgcatttgacggagcgcttgagccttcgctcttggtgtcgttgtacgtctcaccattgaaatactgctcctgagaagccttctcattggtagcctcgtaagggaagcttctgatgattccgttcttctcattgtcaatgtcgtgaacaatggcttctgaaacatccagaggctcatccttcagaaccttgagcagccacttcgtgtccgtaccagaacccttacgggtaatcttgtagttggactcggtgatgcttccctcttctgcttcctcgaatagctgagcgaagaaggttgaacccatacctcgtgaaacaatcatggtgcgaggttcttcgccatccatgaaatcaaccagagcgttgacgtagtagtttcgcttctgaccgtattccttgtcacctgcacgagcacgttcgcagccgtagcagcgaccctcgtcctcaatggtgcaagtagcacgatacatacgcttcagagacttgacataaacaacgtgctcgacggcaagagtgccggttccacgcgtcttgtcatagttctctgattcagggtccatctcctgtaggaaacggacacgaacggactgaccatcattcaggttcaggtactcaaccttcggtgcgtttagacggtcttcacgttcctttgcagcctgcgcctgcttttcgccaaaagcacggatagctgcgagaccctttagagttgacatttagtttataatctcctgtgttatttgaggctaccaagaacctactgatgttaagtatagcagaatggggctttcagtacaagccccacctgcgatactcgaagtttgatactgagttctggacacaggttttaatctccttgtcatccagagcgccagcatcctttgatgcatttggataaacctctttgtagccatatgaagcccacttaatcctctttcgaggaagggcctgcacaatggcgtttccgaggtctcgtcctggattgtgaccggtacacttccaacggcacttcttgcataccggtgaaatgtggtcttccttgttgtcgaagtctgtcatgatgataatggtattgaagtatctgttgaaatactcaatgtgaatcgtactgaggtgacctccaagcaatgctaccgtacaagggtaaccagcttgatgtactctcattgagtcaaatgatgcttcagtaactaccactgtatcaccggccgctcttgccctgttcaggttccacatagttcgagagactggcagacctggtgaattcttgaattgcttaccctgaatagaacgaccgatgaggcccacatgctttccgtcaggagagtgcatgggaacaatcaccatatcattcctggaagaataaccaattctgaagtgctctagagtctcctcttcgaaccctctgtcttccaccatgtatcttacagcttctggataattccagaaatcatcatacattctgttgaatggctcgtctgggaagatgttgaagtctcctgaacttgccatacgcttttccctgcgttctgccagggttacacgcttcgtgtccttgtgcttcagggtgaaccgaagggcctccatgagattgcatttacgcatacgcatgacgaggcccgttaggtttcctgattcggcacaagcggcattgaagcagatgtaggtgccatttcgcttggagacggagaagcttggggtatccgtgtttccatgaaatgggcagtagcacaaaaagtcgtttcccgtctcgccttctacctctacgccaagcccttgaatgactactgcgatttgttcttctgtgtagtcattgacactggccgcccattcactatcccatgaatttcccaagattttctcctcccaacgtatattccataggctgttactacaaaatcatatttaccatcttgcctgtattcagttgaaaagtatggtcccaagtctagcacagggacatatccattcgtgcgcatttcttttaccagcaagtcttcatagtgtgctctcagccgtagaaaatcagcatcatcattgataatgccgtccacctgaaatcttttgacgttgctgtgcatcagaaaccgccgtaggattccctccagatacccttatccaggtctgcatcaatctcgaattcgaaatcagaaccgtgtcggttcttcctgcaaataatctgcataaggccactgtcagggtcacggtggactgcaaaggccatgtcagcatcatactcgatggctcgtgaccaggcaacctggttcagcatcggagccgtcttggagctgttcgtgtcgtcagcagttgcagcagtgatgtcaatcagaggaatattattcgtgattgcaagctgcttgaactctcgtgaaacgcccattgcacgctctacaggagagtttgaacggttgttatttgagaatagctggtgatagtcacagattaccaggtctggacggtgctgttcaatcttagcctgaacagtattaggagtaacgtcaccaacgccctcgtttgagacgatgatgaattctggcttgttggtcatcttcttctgaccccaagcacggaaattgtcgatgtcaatctgaccacgagcaaagtctgttgctgagaacagaccagaacccataagagtatagattcggtcacgcatgttctcaggagacatttcaagagagacaatcatcggcttgaacccacgttcccaagccttgcaggcaaggtaggaagtgaaccaagtcttacccttaccaggccagccaatagccacgataaggtgaccaggagccattccagtaggatatgcagcgtcaatggccttgatacctgtaggaataccaggtgaaccatttgctgccgcccgttcgcggacaacctcgaagtgtcgctcagcagattcgtagtctgtgaggtccaggtctcgtacattgttggtgaacttattcagtttaccaagctctgctgagagcttagccaggacacgtgctggtgcttctgtttccagaaagtggtcagccttgcccatgatttctgtcattcgagcaccaaggtactcattcttcaattggtcaaggtaatactcaggttcacccttgacgtcgacagcttcgagaagaccaaatcgttcctggagcacagagacgtctggaatagccttgaactgcatgtaatacttacgcaatccaagccagacgtccttgtgcgccacgaaaaggtcatcgacgttgtcagcgagtagggatgccacctgctttgaactacaaatggcattgatgagctttgcctcagttgtcgccacttattgcctcctcatttttcttttccagagcagctacgagagctgccgttctgcgcagacgttccctactcttccgggcgtcctcggcaaccttcaaccgattctcgtgaagcttgtcgtacttgttcaagaagacttgcacgtcatgaccaggagagtcacaagtgaagaagtattcgagagccttcctcgcgttaccgtactccaggtcatcaatagcagccttgaagccccactgtagaacattgctgttaagcatagggacacggccgtacttctcacgaaagagctttttgtatacgccaatcagctcgaatgccttagcccttggactcttcttcgccatggttatccttcaagctcctctacagccttggccagacgctcaatgagctttgcctctaccttagcaaagacacgttcgtacgcatcatcgaaagtctcaccgtttctaacataatcttctaggccaacactgacctttacgctctggaagttaccgagattctttgtatatccaatctcagcactcactgactgactcatcgttttcctcctcttctccgatatcactcactactgagaaaccaaacggacgaccgtcctcccgttcttcgggggcgtcgtcctcctcttcgtcatcgtaccccagacgaccgctgatatcaacccatgctcgggcaacatcaatcagggctcgtgagtccttgcaatctacagcgaacctagccgcaacgtcaagtgcggttgctgcctgaatgattgcaattcctggattgagcctgtctccaatgggagtgtacttagctgtagccatctgacctaccaatcgttttcttgccaaaccggcgtaaagttgccgtccttggtcttcgtgtatagaaccacatcgttgcgcatcatggcaagtagctctgttctggaaggaagattgtaagtagcagttggcaaaccgtccttacgcttcgcaccataccccctttgtaccagaagttcatgcaggtccagaatatccttctcagaccacagatagactcctggcctctcctctccatcgagagaataggttcggaacggtgttctgatttgctgttcctttatgtagaggaatagactgtttctgtgcctaccgagcattttctgtacttcagggccatagaaggctctcttcatattgcgctttgcgacagagaggacatacaacactctcttcttttcaatgtagttccacgccagtagcgtatcagctccacgatttatctgaagcttcttatgagggataccgtctaggaagaaatatttatctgccggtcgctttttagctgctggccttttctcttttcccattaggtctccaaatgataaatcccctgcgcacttgcgtacgcaggggtgtgctaaattgagtatagcacctggtctatcacctagcaaacgttgctgaatgactcttctctctgttcatcagccactgtgctagcttgttcttggttttatcaagcatccatctggctccacattcaatacatgcaagctctagatgagtcttatctgaaaagactctatcgatgaatatcctgccaccacacttcttatggttcaattgttggcaccttgtcctttgtatcaacgacaagggatgcactttcggagctgccaacaccagcagaacctacagaagtgagcagggatacgagcgcagcaagacctgcgacagatgcagcgtgtacccaacttacgtcaattagacctgtggatagcgctagaattgccactgctgactgagcgaatgtctttagtgctctttcagccgcgtccttccaaaacttacttgttctcaagactaaacctcccttgcctttttattcattatagcatgggaggttttatctttatacgctgaatacctttgtaccgatgttacagcgataattgatgaccggcacaaggtcaatgaatacttcgttgccggaaattacgccataggcgaatccagcctgccagtcaggtgacaggtcataatcctgtccagttggtacgcacatgtggccgatttcccagcctcgtaccgtccggtcttccagtggagccgtgtgagcgtaatagccctgtcgatgtgaatgcccacgaatcagagagacttcgaacttcttgacatcgttcctgacagattcagcagaatactgagaaatgcttacgccatgatgagcgtaaatatctccataccgccttgtcggccgctccagataattgtgccacacgaagccgttcgctgcaacaccatagatgctttctggagtatacactccatcaatcaaagactggggattgttcttgtccagccacttcacatgtcgatgccaaccgtggttaccatcgaagaagtgcttgtcagcatctggtgcctttgcatcaatctcttctagaaaggctcgtgtgcctgttactccagggtcattctgggagaagccttctcgtgaggttccttctgcccaccggcccgttccgtctgcatcgtcaatgtctccaagcaggtcaatagcacctggcttcagatactccatcaccttgaaccataggtcaagcattcgcctgtcatggttcgggaaatggatgtcagacgcaaacagccatgtcatatcggacatgcttgttcctctcgttgtttgaaatagcctaacacaacgtctatcgtcgtgtcaaggtcatgtgctcctgtcgattcagtcgctcatgctccatccaactacacaggaacaagttcttccgagtattgtcagtcttcactccgatgtggtgaacagtttcatcgctgttgagcaatctgcctagtcgggcttcgaagaccatccgatgttcatagtaccaacctccattgaagctctttgggtgttctggtgcgtagaccagcacatatccttcggatgagagcattcgctttcggttatgccagtttcggataggtctgtacattagtacccaacggcgatgaactgaacgttcacaccatagtgaaagtagtttgactttgcactaagctcgtctgagtctaccacagcagtgaaccctctgtggtccggccagtattctcctgtgagtcctctgaggatacagtgaatgcgtgactgcttctggatgacctgtccagttacgacgataggcttgcatcctactgagaagaagttaccaaagtaaacagccttctcgtagtgctttcccttgccgcccttgatatagacatagccagccataatcttcagaccagtagtcttcttgattccatgagcattgtagaatgcgtttggctgattctcaaagatatactggtcattagctgtcatttgattcagcttctcacgagtaatctgttctcctactgaccagctcgtaggcttgtagggggtagttgccaagttatgttctctccttccttgtgtagggcaatctcctctggagaagcttcaatcattgcttcacgaggaaatagcatgacatcaaagaagtctggggagactatctgtcgccgaaggttctctgatatcagatacattttaccattggatatatccttaaccaaagttccttgacggaaacctagcttaccacctatggagtaccgggcgcaagctgcctcagatgacttgagaacaaaggggtagcgccagcttgacagaatgcgttctgatgtgaccctgaagcggaacttgcccttgatgaagaatacacccttctcagtatgcacacaagtccctgtaggatattctacagggactgtgggaattgctggaaggggaggcttacttctcctccaccataccatcaacggcctccaacttctcaagatagccagcattgacatccttgagggtcttgttctcgtcttcaagcttcttcaccttggcttcatagtcttctacagcaagggtgaggtcagcacgaatgtcggcaatcttgtcttcatacccagcagttagctctgcaattctttgagtaagagccataactttaagagtatctttatgattcattttaatatatttctctttcttttctctagtgtaactacaccttagctcacccaaggcggtcctgtcaaatccaggtcatcccttgtggtagagagtaatctttccataagaagaacctgaactgactccatacattactgcatagtcacttgtagaacttccataaacaccgaatccttccattcgatttgcagcagttgaatcaacgatattgttataccagcttgatggaattgtaactgttgctgcttctcctcttgaaaggtaaacaatgttgtcaccgtctgagccttcatttgacatgttacctacagggtcaccagatggcttagtctgatagatatgtcctctcagattgattcctacgccagtgttgttaccatgtgctgtagacattctcttgagatagatagttgcctttgtaggagttcttacaacgccgcccgcgttcagagcatcgtaaatctttgttccatagaaatacaatcctctgtggttgtcgtttccggtccagtcaccctgatagacatcgtcaccgtcgtttctccacataccaccatatccaagacggtaagagtctgaagagtttgcagtgactgccacaggtgaggccaatagatatctgctcagagaaacatatgttgaatggttaccacttgtgtcatatgaaacaactctgtagtagatggtcttgttgactggtagacctgagtggtcgtactgctttgcagcgcttgcaccaccggttacagtaatgatttttccgtctgtggtaagagtcttcacaccagaagatgcaatcgttacagtaggatatctgtcgcttctccagataaccttgacaccggccgtatctgaagctgaagggttggtccagttcagtcggtaagtaccggttgaggtacttgatgacaatggtgtaatcttgaaaccagaaggagttccaggagcagtcgtgtcaccagatggaggagttacatacgtgtcaggagaagttgagaaggaaagaccgtcaagcattacattggtcttagtcgcagccgctgagtatacgtaaacagcaccattggtacctacaacaacaatagttgactgagcgtcggatgtcgttgagtatgacgtactggatagataaaccgtctttggtggacggaatccagatggcaatgtaaacatcggagtacctgaaccctttgtccatcctgataccgctccacggaggtatacatttccgttgctagagaagtaagaaggagtatacgcaccagaaacgacagcaccagtctgcaatgttgcagcttcccaaggagggtcgccatctacgtcaagatgtccgtaaactcgcaagtttctaccagtcgtcatatcttcgccatcatttgcatatgatgatgcgacgaactgaccagatgcatttgtcagagcaaatcgtccagggaatgaagcgtcgttcataccatagaatcgaattgctgactcttctgtacctgatggtgagaagtttgttacctgagtgcttgctcggttaccattagagtctgtgtagtttacataacgcagaccacccgttccaccttcagtctgaaggattacaccaggatacaaaggtgaatcctgtcgtccaccgacatttcctccccatacgtttggaggaaggtcaagctcagaccttgaaattacaatggcaccactgctctttacgtaatcagactgaagagtaatcttgcttgctgatgctgtacggaactgagactttacacccatttcaagctggacgtcatcgacataaacaatgtcaccagttgcaccattgaaccatgagaatgagaattcaaccgtgtagcaagtgtcaggaatgacgattggagagatgaatgaatatgaagtccatgccgcattggtaatattcttctctacgaagtaaccattaagcattgagccagcttcatcaaggaactttacaacgagtcttactgctctaccggtcgccgttcccatggttgcataaccagatacagttacaaactgacctacgagttcctgattcgtcaaggttgatacctggtacttaccaagaagctcggtggttgatgggcttgagattgttccagttgccagaatcttcagagatgaaaggtcttctcgtcctaccgtcgtttcctgagagagcgtggtatttgccattccgacccatccagaaataccgtcctcgaatgaggcgtcatagatgtagttagggttacggtcgtaaataccgattgcaacgctaccgtagtcggagttcttgttgatgagtggtggggagatacgcaggtgtgcagcatcgttccagtcaccgtccatcgtatgaccaatcatacctggggactgttcatcgaggtctccagaatagaacctaatggcgtggtcgaagatggagccctgaatattctcaattgttacagccttttcaccagacacgtccttggtagcaaccttggtaggagcaccgttctcgtatgcttctacagaaaactggtcgagctgtgcaattgagccagtcgcgatatcccattcgacataaggaacgaggtatcgaacagactgattcagagaagctgggtcataagggtcctgacgctctccctgaacaccgagcaaaccagttgtaccagaaccaccacgaccacggacataaccagttacggtaatccagtcagcagtcgtcgttgagctaccggtcgcaatctgtacttctgaattgttcataaccatcatgtactgattggctgaatacccagaaagcaagctgtaatcggttggcattgcatgcttcttggtaggcgttgttgcatcgtcaacgtagtcgaagtcgatgatgttgttggagttgtcgaatccgaacaggccgaccttgatacgaggaggcgtagtaattgttacgtcatcgtaccaagcaattgttccagcggttgccgttccagtgatacctgaattgacttcgaatgaacagttggttgcagtcagaggacaagttccctgtacattgacgtctacccaagttgtaccatcaataggaattggagttcctgttccgtcgactggaggggcaattggatttgtgtctgtagaaactgttgttccaccggcagtcttccagatgatgtttactgccaggttgtctcgtgcaactgttgagttaggcaggtacttcattctgaaggtgtaggtgtatccaggcttggctgtggccaatgcgtaacggaatccgtaggttcccactgagctaccattctgagcaatcttcattgacgctacgccagtgaagtgcttagtagtgtctcttgtcatcaaggatgagctgtaggccgtgtaaccgctgacgtcagtctcgaatccagtgttggagttcaaggtagccacagagtatgcacggatacgagcagagatacgatatagaatagtagggtcataggcgacctttgtgacaccggtcgcatttcgcagaacgagaccagggccagtgaattcgaatagagattcagcagagtaagcaccagggtcagtcttctgcaagacagagccactgatgagccacaggttggagtcctgcatgtagtcgaacagctttgcataggtgttgttctgcataggagcgtccagtgcagttaccttgattgagttgactgcaaggttacggaattcggcataaccatcattacggatagtccatccagtcgtaccggccacataattggatgagtggatacgactgtttacaccatctgctgatggattgcttgggtcaccaacgacaatgcgtcctcgaacagtggcgtcaccgaattgcgctccaccagttacgttgattgaccatgctggctgaccgcccaggccattggctgaagcggtagaacggatttcaccagttcggataattgcaccatcaattgaagtagagccaggtggcttccactgtgatggagtcgttgctccggtgagctgacgctcaatctgtagaccatcgacataaacgtctcctgccgtgtacagagatacgaatgacaggaagctggtgtttgcaccagtattgaatgttcctgagattctggtccatgtagccgttgcaggaattacgtttgaagttccaccaggctgctggaatgtaccgtctgcaagcttgatagcaagaccaacagtcttcgcaccggctcctgacttcacatagaagtaaccagagaagatgtaattggtgtttgcttcacaagggacgttgtaaacggatgagcttgaagtctggtagacacgtgagaacgttccaccaccggtccagttgtgcttgagacactgagtggtatttctaccagcaacgtctgtcgcatctgagattgagcctgtggtagttccaccgtcattgaatgtaagcatcttgcctgtgtaccaggctggagcgaattcaaagtctgcataagctggatgcatgatgttctggctgtcctgaagctgcaatgctgctgctctaatagtaccgccattgatttccaggctgttattggttagctgatacccgcttgttcctgctacatagttggtgctctggattacaccaccagtatctacagtcagagatgacttgataagcagtgcattgataattccagtaccggccgtaatcttgtttgcatccaggttgttaatcttggcagacgtgatggtggcgctagcgatgttggtatcagagattagaccaacagtcacagcagctacagcagaggccgcagacttgagtcccagacggtcaactgcaatgactctgacccaacgggcctgagtcgtgtctgtgacgacgaatgagaaaatctcagaaacgaacgtgctaccagcctcgacctgcaattgtccaatcattgtagcgtcgcttgcggtgaacgtagaggtggttcctacgtgtacttcaaggtagctgacgtcagcttcaagacgtccggacgtggccttctgcaaagagtggtttacctgaatctgcaaagtattgaccgcagccgttggggcagcaggagtagacggagccgtattggctgccgtcgcagttaccgcagttgagtaggcacttctgttgacgcttctgtcgtatgactgaattgagaattcgtactggacacctacgacaaggccgccgattgttgcatttgtggcagcagcaggcatgtctacgtagttgtagtttgtggctccagtctgcctgtatcgtagattatatccagcaaggtcagcagcagatacggcagtccatgacacagaagccacagcaaatgctgagtcattcgtgtctgtcgccattgtcgctgccaatccagtaggaacacctggtggagtagtgtcgacatcagttggtgaaataggcgtggctgataccgtgacgtacgcagaaatgctgtcgaataggtcacgtgaacgaatcttgaagtaatggactacaccgagagagcttgagtcatacatcagtgtattaccagttccgctgtacaccctgtttgcattgcttggggtaaagccggaacctgatgtgctcatatatacgtcataggctgcgaggtcatcaattacctgtacatcccacttcatgcttacacccccgacaatgccggtcgcaattacaccggttgggtcaggtggtggtgggtttgtagcagaaacagttgatgaaggcagagatacattaccagtgaggtccactgcgtaaatggtgcattccagagtagcctgtggcgtcgtgaagaatgctttgttagtctcgaatgccaggtcatagaaagtgtttgtggtctttacgttgacataagagccacctggaataccaatctttacaatgtaatgtgagaagtcttctagaggagatgcgtcggcattctgagtaaccgcattccatttagcggaaaatgcggttcgattgactacccaagtcaatccagttggagcggctggtggaagactgtcctcaattgtggtgagcggaaagattctgctccaatcggaaacatttgttccgtcgtttgctctgagctggatgttgtagtctgttcctggcgtaaggtctcttagaagaattctcatatagtgatttccaatgcgtactcaatatccatctcggtgtctgaagtctttggcttcggagtcccgagaactgacctggctacaagggcgtagtctgtactgatggtgtctgcgtcttctgccctgataccatcgaatactgcatttcctgtgccggtcgctgaaacagtgattgaagtgatattgctccagtcaggtgctccagttgctgtgaatgcagtcttgcccattgacttgacttcgtagccagtagctgcgacgaaactgtattcgtaatagtttgtaaggtctgtgctgaatctaattgctacattgttgattgcagcatctgcattgtaggccaatgacatgaagtctgcatctgaatacacagacatgtcaatagcaatatcagacagagtagagctgcttcctccagctacctgcaatgcgtctgttccaattcttgccaaggccgtattccacgtggctggtgtccaggcttcggtatcagagtcaaagctgacaatgacagtgcttccgcctgaagtctgtggtggagttccgaaccaaagaccaacctcataaatctgaccaacgtattcctgtggaatagtccccttgaaaacaatcttgttgttcagaatatctgcggacaccaaagagacaggaaccctgtcaacctcgaatttcagtgccgtatcgtttacgttttcagccgttgagccgattccgagcgcgatagattctgcaatgcgagggagatttcctgccaagtatctgaagattactgactttccctgagtagttatcatgtctttgtcatcctaacgtcaacctgaattgaatcatcaacatctggtacttccaaaacaacgtccacgacctgcttgccggaaccatcaaatcgaatggtctgacttacaacaatggctgtatccggtggatagagaacgattgggtcgttatcctcaccggtttcctctggctcgtccagagttacgtcactgctgtcgtccacatcgattacatcgtcagtgtcaatgacttctgagatttcatctgttgtgacctcaatctcgtttgggtaacgagcgtcaacgaccccaggtggtaggaagaaattggggtcgatgacgacatcaggcttcttgatgatttgattgctgtttgtgctcatgaggtaattatatctgagctattactcaaagcaaaatcaaatcttgactcttcgtagagtcagcttcgttgtctcatatccttggtcgtaacctctggtcatagcaacaatgaagtatctatgtgttaccgggtcaaagtccttttgtggataattgacggacacgatgtctccgagctgaagcaatggattaccaaagatttcagcttccacctcatcgcaaccatctgcccagtgatgagtaatccagtcagcgagagccttggcaccgctttctgtctgaatccaatcgggctgaatttcagtgtctacttcaccacgacgacggattgcatcatcattctgagagacaatagtcttctcgtcttccttcgtgacagtacgaccataaatcatcagcttctgctcgacaggattgtcaggcccgaatgtcagagtatcctcaccattgacaattgcattgtttcgataagcattcatcagaatgaattttgcaccaaacgggtctgctgaatattcgggacagacaatctgtgtatcatttgagaagtaaaggtttgagtgaaccgcagggaacttctcaaactttacatcgaattctctcatctcatgacagattgcaccgaagtcatcaaagaatctttgagcatatctggctctgaggaatagcttcctcttctttacaatgtgacttgtgaagtgccacttgaatacccattcagagtcggcctgagttgatacaactccgcccctgattctgtcgaacatatcagtcttgtcgatatgcaggtcttcagtcgtgccatttccgtaaaggtattcaaagtcagcatgcgtacttccacgagtgaatacaccaaagcgtccagtcagaggctccttcttatccaccggaatggtgaaggtcatcttatgaataccgttgattgccacttcaattacgtggcctacaaaggctcctgggtctccataaacaccgttctccattcggatggcaatgtcgatgtcgtaccaggtgttcttggaaatggccatagccactcccttacccttgtcaggaccgaatcgttccagctttccattgcttctacggacatagaagttgatttcattctggtactttctgccaccgggcaatctgtcagttcgacagatttccatgtagtaacccttgtccttcgtgcccagattgaaggcgataccagccataccatggttatatccagtgtccctgaaacgtagcctggtgcccacgtaccaaatgcttttatccagttcgctaccacgtgaggcaacataacatgtatttacgttggtcttcgttgtggccttcaggctgatggttgactggtccttgttgtggacaaatccaccggtccaggtcttgtaatcaccctgatagttagctaccttgactccatatcctgaagcatcgtccttgtgaacggcaggatatgtattccagagacctcgctcggtaatgcggaaccacccagagaagtagttcttgaatctcagctctgggtcggacaattcttcgtcaatgttcttcttctcatctgaagaagtgattgccttgaatgtcagaacctttgacttgttgtagtaccagtatccctttgcgtcgtaacgcatcaactcaccttcacactcgatgattcctgaataaggccagactgccgcttcgctgccggtcattctgataaacatctgtgaatcagtcattgtttctctgagctggcttgaacgcaatacaacatcgccttctggctgccatacgatatccatgactggagtacggccctgctgagtctcagacatagttgttttggcatatcgaactgtgaccttatttgcctcgaagtcatacttctgagaaaggtcgacaatgtcaggctgcttcgttccattcagaacaccatcgagctgccaggcaattggattgccaaggttgtatgccttgtctctggtgagaatctgcaagattccgaattcatcaaagtaaactgcactctgagtcgtaatggcaaggctactgaagatttcccatacggtcttctctccatcagtccagaagaatggaacaatggtcgccttgtcgtcgtcaatcttgtcatacagataatcagtgaatccgataatgtcacagagctgccatacaattcttccaagcgtcatgttctcgaagaacatctttggaggagtaatctcctgcaagaacttggaagcatccttcaagctgactgttgcaatctcttctccctgtccggaccatgaatcggcaaacatcacatattcatttacataaaccatgccacttccgcctaccggcgtcgtgtcataaccaattttgtaagtgaactttacgttcttgtcgatgagtccatagtaaagagacgtgtcatttgtattattgtaccggccatcaatatttgatagctgaatgctggcggtatttgatgatgctgagccaagaggagtgataaagcttggcgttcccatgtcgaattcagagctgctggtcatcagagtatcggtcaaatcctgctcaagacgtgcagacatctcgataagctccaggtactttcctagagctgagattgttcttacctttagtctgactcccctgataagcattggattctctcggtaaacagtcgttccccaagttccatttgcctgacgatatacagtgactcgaccactggagttaggagtgatgtctccagagactgtcgtccaagtagttccattggtcgtgatgtcaatgtcgtaatcaactggtgatgctgctgatgtttcgaacaggaaatacagcttattcgtccaggcatttaccttgtacacaatgtaaggctgaacagtctttgagaatccgttctgaccactaataagggtacttgctactggagaggtccaatacttatatgggtcatccaaacctgcaaggtagtaccttatgccaccgggcctgtctgtgtattcggaaacgattccttcgacagaggctctggcctttacaatgcccttctttgaagggcgtagcgggtcggcaatagattcaattgggtagtattctgggtcgccctcatcgtattccgttggggtgctgtccaccgtctgaatgccagagtaacgattatgattccactcagcaataagctggggtatggcctttacggcatacccctctttcaatagcttgtttacttcaaatgtcgttgtctgcattatacctgttccaatgatactgtgacttcgtggaagtcgaaatgacctcgctttgacaggttcatgttgaagtcagaaaacatgacctggaatgtttccactgttccatctccatacgtcaattccaatgtgaaagctcctggagtggcgtcaaagaatgttttcatttgacgcgcaccccagaaaccatctaccgtataggaggaggaatgcggtaggtctgtccattgcacaccgaaagtacgcttgtctgcgacaacatactttctcattgtgccgtccaccatacgctgcttcttttcgattctttcgacatcgatagaaagctcgccacgattgtggtctgtaatggcattgccattccagcgcatcaggcggggctttgcaaataccattgccattacttaaccaccctgctccttcccttgttgccctcaatcttgttgagagtcctggtaattaccttttcaaaatcaatctcgttgttgattgcatctgcattgatagtgaaattgtacgtgttaccgcttcctgagtcaatcttgtcaattccgctctcaagctttgctgtgagtggagctgtaagaactgcctcattcctgtgaaggttagcaatcgtgttgtcgtatctgacctttccaccagttgcaagaccagggattccgaaggtgcctggaggaatgtcctttccacctctccatgtctcgaagtgcatgtgaggaccagttgaattacctgtgttaccagagtaaccaattagctgtcccggacgaacactctgtccagcacttacacttctctttgagaggtgagcataaagggtgctgtccatgccgtttccaatggtgatgtatcgtccgtatgaacggtatccaccatttccatttccacggaggtcagtagacgttgtaacgttacctgccattgctgccatgactggagttccgacccctacaccgaagtcagtacctcttggcaggttggagtggtgagcccagtctctggagaccggaccatttacaggtcgtctgaagccagcgttgcccatcagagcattgaaagtagtagcaccgacaactgccttagccatcgcttcccactttgcgtacgcgcctgggtatccagaacgctgaactgcctgagcctgctcggtcaagctgagcttgttacggcccttcattgcaagcaagtgcttgaagaactgagtagacgcataggcaggattcagaatctgctctgccgtaccccagccctgagaaggacgctgctggaatagaccaagagagtcacggtcaccataacgaaggttacggatgttggattcctgcatggcagtcatgatggctacgataaggtcattggttgttgctcccattcccttaccgacaccgataatggttgctgcattgcccatctgctcagcagagaggttgatgcctccatacataccggcctgtccaggaatagcagttccgaacattccgattgacattgcctgctgtgctccgagctggatacccttctgaatcattgcctgcatcataccggccattcctgcgcccataagtccagcaagacccataccgtcatcggctccaccaattccgaactttccagagttgacgttttccatgaagtcagtaccgtacttctgtacagccttgttcttcatcatgaattcgcccttcttggcacgaatatcaacctctgaatgggcctttccaccagagtaaccagtacgtcctgaaccaccatatccaatgagtccaccctcgtggtgagcatcaagagacttattggtgccggacttttcgttcagaccagactttggaagttctccagaagaaacccactttgagaactgaccaattgtaagaccgaatgcaccctgagaaatctcattggcaacgtccttacctagcttgtcccatgcaatcttgctcttgagctgttctgctgcaaccttgacgttacggttcagactgtccttgatgtacttggaccagtcgtctcccttgtccttcagattgacaccatacttcttgtatgccgcctcaatcttcttgatttggtcatccaattccttcttgtttctaggaacgaatgccttcagagttgcaagctcattctcaattgcagtctttgcagcgtcgtacttcttctgtacagccttctgcttgtcagaggtttcctttgccagagcttcacgctgagcctggacagccttgttataagcttctctctcaatctgaagcttctttgcagcggtttctctttcaatctgcaatgacttattagcagcgtcacgctgagcgttaagagcttcctgctcacgctccttacgagcctcaagctgcttcttctcgacctcttccatctgctgaatgaccttcagtcgacggtctctctcagcttcaattgtgtccttctgagcttcaagaccagcaaccttcttttcagaagttgtctgacttgctgccgcagcatcctcagtctgccaggaattgatgtcggcctgcatgttgttaccaatcttggcagcctcatcgaggttacctgaattgatagccatgttgaagtcgatgttctggttggcaagagaggcagctcgctgaatacgagtcttttccttctcgaagatttcctgacgctttgcctcggccgcttcctcagccttgattgcgtcgtcaatcttcttgaccttggcgtcgtacatcttgttaatctggtcggtacgcttctcccacttcttgtcgtggttctccatgagcgtatcccacttcttgtcaaacgccttctgcttcttttcgaacttcttgtccagagcttcctgctttgcctcaaagcgcttttcagttcgctcctgacgtgcgtcaaatctgtcgtcagccttctcctgagccgcgtccaattcctttgaacgtgcttcggaccatgcgctgattccatccaattcattctgcatctgctcatcgagaagctttccagcctgcgccatgtcatcgtcacgggcaccctggagtgcagacttataggtatctacaaagtcaccgatattgtctgagttagctgaccatgcatccatgttctcaaggagtgagtcacttgaattgtcgatggccttctggaatccctgttctgctgaggtggcatcttcgagaccggccattcttctgtaaatgttcagcgttgctagctgctcatcggcagagattgcctttcctgtcttcatgcgctcgcgcagggctgagtggtacatgtcctcggctgccttcacaccaagaatctctgtcttggtcttctgcaattcaggcatgtacttagccaggtcgtcaaagttgtaagttgccttgccctttggaataccctggtagtctgcgaattccttattgaagtccttgatggcgtcagcggaacgcaatacctgattacgctcagactcattcatgccaagcttaacaccaagggtagtaccttcggcaccgtttccgatgttggacttgctcaagtagtcagtatagtccttgaaggtctcaatacccatcttcttgaattcgtccttgtacttttccttgtacttcttgaatacggcaacctgctcagcatttgctgactcggaaatcttttcgaataccttcttcttctcggcatcctgagtgttgtcatagatgtcccaaaggtccttagcattctgccggacggcctctccagccttctgctgaattgttccctcgtttgcaaagaatcttgcaaagctctctgacttgtcctggtcgaacttcaggttggttgcgtctctcatatcctgagcggcgttcttcagtcgagtctcgacaaccttgctcacatcatcgaagtcaatctttgccttcagcttataggtgaatacagccttgtcatacttctgacccatgattgcaagggcagttctggtcgcttcttctgccgcgtcctttgtaccaccgtgaagacgaaccttgacaccttcttcaatagcacgtccccacttctcggcctcgtcagcatcgtagaacttctgcatgtcataataggcatccttgttctccttcttgaacctgttcatcttgtcattgagggagtcaatgttctgtccgccctgagcgacaatcttctgctgttctgtgtaggcgaatccaagagtctttgcccatgactcagatgacttctcgatgttttcctgttcctttcgagaagccgcaatgttcttattgatgatgtaccaggctgcaccaattgcaagggcgaccgccagcattgttcccatgactgccgctgctccaccgagctttgcaaccagactaccgacagatgaacttactgcacccatagctgtacgtcctacaactgcaattcttccgaataccgctgtggcagttcttcccagagcaacgaacgctggggctaccattgtcttgattccagaagctaccaggccaagaggcttgataagcattggaccaattagtgccgcgccaattgcaatctgagaaacggtgtacatcatgctgtcagcgtcggaagccattactcccatgaatcctgctgcgatagctacattctggaaactgcttcccatctgctgccatgacctacgagttgcagcagcattgttgttaatgcttgtagatgcattctgagctgcctgccaatttcttacttcggcctgagtcagggtgcgtccagaggcgtcacgatatccacctgctggatgaggtgtaattggaccaactacaggagtggtgattgtcgctggtctacctgctgctgcaccgcctgcggctgccgttgcggctgctccaccagttgcagcaccaactcttgatgcctgagtcaatccttgctgagcaactgtggctctctgcaatgcagcagtaaggtcatttactactgcgataagggcagcggtctgaccagcctgagtctgcattgctgcacctgcctgcattgtggccagacgtgctgcgacttgttcagggagcaacgctcggaacctgaatacaagacctagaaggcttgcgcccatctgcaatgcctgtccagccaagttagcaaacagacctacgagcatgacaattggaccggcaatagcaccggcaataattccaatggtggcgaatgtctttactggaccaggaagactgttgaatgctgagacaatcttggtgatacccatgacaaggaatccagcaatttcaaggaatggcttaccggcttctgccagctctaccttcagagtttcaattgcaatcttcagacgtcctgaagcagacttctgaagctgctccatctctcggctggcactgtctgcccatgttcctgattcctgctgggcaatcttgtaagccttaccgacctgagttgtttcgtcgttaaggttagccatttcttctacgatggcctggagtcgtgtagtctgctgagtaccgaatactgatgcgaagagagcctgacgcttctttccataaagaccggctgttgccttctgcaatgcctggaatgtaggaatgacttcaccattggtcttgtctgtaagctctgtcagtgactgcttcgtcagaacctcgaacatatcctttgcctgctttgtaggcttgaggactctggtgaatgatgccttgattgcgttagcaccttcgacagcattgattccacgagccttcattgcgacaagcaatgtaccgatgtcctgcaaagaaccaccaagggacttcataggcccggctacctttggaatagccttggagaagtcttcgattgacagtgaggttgcattttctactgcgttcatgtagttgaagtcttcagccagctttgaagtgctgtctccataaacagactgcaatgtgatggtggtctcaagagccttctggtaatccatttcaccaagagttgcgagacgcataacctcagtggtcttaccgataagctcgttacccttttcaccggtcgctgccaggtctgcttctaccttcaaggtatccttcatggaggcaccgaactgctgggcagcattctttgccgctgacagagaattgactcgaagacttgatgtctcctgagcattctttgcagcgttctggtctctggttacagagaagtcgtataccttattgatacgagtcatctgggattctacgtcatatgcaagctttcccattgcggcaccggccgcaataacaggaacggtcagaccgaccattagctgacgtccagcccactgagtgttcttaccccacttgaccatgttggctgctgcggagctggtgacctggctgacgagacccatcttgagggcagtctcgctcagtgcagtattgaaagaaatggttcctgcacggactgccgccatcgtttgacggaatttacctaggcgcgctggggcatctctaggcacgatgaggtccatagtagagccaccacgtacattggtaccccactgcattgcagcggctctactcaactggtactgctcacgaagaacctgattgaatgtacttctgttcttcagagcctgacgaagagtaatatcctgcttcagcagagactttgtataggtctctgatgcggaggcaacacgcattgtctcggcagttacattaccgagattgcgaatgtcttgcctgaatgccgcctgatgagtagccagagcagcaggagtcataccgcgcgccaaattagcctgcatggctgcaagctgtgtgttgatggcattgatttcagccgctgccgcacggaagtccgctgtagctgtaaagcggatattaatattctctattgtcagtcatctccttccagaccaattccaatttcagcgaattcaagactttcggttgaaacgcccttggctttggcttctgctctgcgcttgacgtcttcgaaggtaggagtctcattgtcagcattatcatccaggcttacgcccttcaatgcagccgcgaacttcttgtcggagtaatcctgttcacgctttacagcgagaagtttttctagttcggcgagtgtgaggttttcctcaatttcttcaaagttcttccaaatgcctagcataaatagttctgcttctagttgagctagattcagctcgtcccagcttgcttctccagtgccgccgtcgctgcggccagcagttccgggtcattcaacttaacccctccacagatttcgataatcttgtggactgtcggcatatcaacagcatcttcgtacttgtcagtgtcagcatactcaggataaagagatgacagacatagctgaccaacttcgattaggaagtcaactacctggtcatcttccttgagaccatccagttccttgaacttcttcatgaactttcgtagtcccttaatgttcaacggcttgagtgtcacctcaacaccgtcctgtagcacaatctcctcgactgtgtatacgcttgttgccacatttcctcctatgtgtttgatgtaattatatcaacgctttgtcacaatccaaaatgcgaaagcccctccgtagaggggcctcgcgtaaaagttatcggattaggcgatgttcctgtccttaaccacaccgtattcctgacctgagaagcttgggtctggaagcagtcggaatgagactgggaataccgttgcctcgttcctcttcagtgagtgagaagaagactcgattgatagagcacgtcttacgtgatatacacgctctcgcttggtgccagcgtctgcacgtggtgcaggaccgacaaaggctacggaacgctccgttggctcgtcaccgagggaaccagcggaaatcgctagagtctcagatgttgcgtcgcttgagaagctgctgttctgctgaccccaaacaatgagaaggttctccagagtagcctctgagaaagtggtgttaaccataaccttcatagactgcttaaagagcttcgcagagtcaagaagctggtcgacttctacctctccgtaatctggctcgtaagaaacctcgacaccttcactggtaaatccggagtgcttccagtctgcacttgcgtctagcgcaggtgtcatgctgtcggtaccggatactgctggaagggctggtgcgctggtccactcagtagagtcttctgctgataggtagacggctgctgcaccgatgataatgttcttaacctgatatgccatgtttttctttcacctcttttcttgaaaaagtcgtctgtgttggctagacacgcttcctcaataaggccatgttatcttgataagaattatcaagcaaactaggcgcgcataccgtcttcggtatcaaggtctgaggtaaagcagaggcgcagtacaaccattgcagaatggcgtccaccctcttctgttgctggctctattgaagtcatattgagtacacgaatgtacttgaagttgaatgctttctgtgccgggcttccgtttgttgtcagccagtcattcacctcatctgctgtcaagtcgtatcgcttgaacaactgattcagatagtgaattgtctttctgatgagttcttcgtcgtcacaatacagaacataagctagctcttcatgctccagccaccattcctcgtaaccacctgtctgagcatagttgtaaacgaagaatggaatgtgtgctggctcatttgtgaattcaggctgctgctgtgctggaatgattggagaaagcccatcataattggcaaggttaagaagaccctgctccacgagcttggcctgagtaaacttgttcaatgcgtgagtgccggtgatgttatacgtcatttgtcttcctccttgctaggttctttctgtttctttcctgtaggaattcgttggccagcatcattcctctttcgaatgcttccttgttgcctgccacacttatctttgcagtcttcctagtgccgaacttcaatttcttcaggaacctactcatactttttgctgacaggtcgctctgaagattcttcttgatttcagaagtgaatacctggttggctccagctcctgtccaccagcttgtccaagctgcggtgaaggctccagtggtacttggacctccaggactcttcacgaacacaggtccctttgtgaaaatgattctgtcgtcaactgggaatgccagcatctttgcacgcttaggcttgatggtgactcccatattgtattccataatcattgccttgtagatgaatctgtgcttacgcttgaatggcttcttgtcgccttcgggaaagggaactggcaatactgacgccctgaattcaaaggaagcatagcgctctgctccacgacctctcaatacgtttctccatagctgagcctgtggcacaccaattcttccccagtcatatacgtgatggaattgatttggtgctgatggagcgatgacagacatataagcgtcaaatctttctgccattgcgctatgagcatatgtcagtacaggtccgacatttacgtctgacccgatttttgctgatagcccatcgataaagcctccaagaccggagacttccatcgtgtcagcgttgattgccagaaaggctttacccttggccactttgtacctgcgctctctgaatcagagctgtattttcaatgtgatttccgaatgggtctacaactggagtaacacccatgacaataaagactgtcgctggtgctccttcaatttcttcttccttccagatgatgtctccgtttgaattggagacattggtgattctgtcacgcttggacagaatgacattgcgtccgaaaacaagagtagcccagtcgatgttctcataaacttcagaatatcgctgggttgttcctgcaactcggataccaccgttcgtcacacctcttacaatacaactgatgacaagggactgtgctccgggagtatcggcgtcttcgtctgctacccatactcttttaatagcaccagagtcagggtcctgtacggtttcccaatgaccaccgggattttcctgtggattggttccaacctgtcgaagaacggttgctcgcatattgaatctagatgaaagaagacaactcatattaccaccatgttactcagcttataaggattcagaagctggtctgcctttagatttccagtgccgtcatatgctgcctgagtgaactgaattctccagtctgctgccttcattgactcaaggtacctgtcacggtatgagctgtcctggcatgcataatcggagagaagcattcgggctgcctgcactactggataaggaactgattcgtatccccagtctcctgtgattgtgtaagtgttacagaattcgaagtcacgcttcttcaccgtgccgggcgcgtagataacattgtcaaatgtgtccagcacatcttcaggcggagcattcttgatttcccagtaggctccaggagcgcccgctaggaaccatccctcacctctgatgatgaagatagatggctcgtaattgaactgataatcagaaacactcgtgaagtcaagaagcggatttggaagggctagctctgttccgtcatttccattcacctgctttgtgccctggaactttccaaagctctggcctgtgtatgcctcgataattcttcttacaaccgcctctgcgtcatagcggtcctgctctggaacatcatccaggaaggctgctagttctgacagaggaatgattggagtgacaacatcaacccacgtagttcttgagaatcctgtcttcttccatacaacgctcagcgtgccatcataagggacaagagaccaatcaatcgttgtcgtgtggacattgccatttgtagtaactggctggtctgcggaaatcactgcgtcaccgcgcttgatatctgcggttagtgttcctgttactggatggtccagagtaacagtgctcgtagtgtctcggtagatttccatgtctgcattatagattgtttcaggttaaatagcaaagaggccccgaagggcctctgtcagctatagaattccttgacttctctcggagaagcaattcggaatccctcatagttctcaacaatgaaatcagcgtcacgttctctgacaagtgcgtaagggttgtccctggtgaatctagcaccacgaatctcgaatgtagggttggccctagtcattcgaataaggactctgtcctctacactggcttcggaaggcttattgactaccggctctggtgtctcaacttcaatctctgcacctagtgacttctggtaatactcccatgtcacaccgttctcaacaagcttagcaatgactgctgccttgttttccttggggtcaatgtcgactgcgaagtcatccgcaatctgcctcagctcatcgacctttagcttttcaaaactcattcatatcctccatacgaaaattgtaccataaaacgctaagaggggaccgaagtcccctctcgctattaaattgtgtgtgactgattaggctgcaatcttgacgttcttgacgacaacgaatgcgtctgcgttctcaatctgagttcctacacggcagaacattgtgtactccgtggtgtccttcttaggcttgaactctcggaagacctgaatctcacgctttacaccccatagcatattgcttgggaaggtaagccatacgtcagcatggttaccagatgcgcctgagtagtctcctgtcagggtctcctcgaatagaggaacttcctgaactgggataccgaatgcattaccggttgtgaaaccagcaggaccatcagtacgaaccgcactgttgataccggcagcggcaattgcctctggggtaacatagtcagcagacgtgttctgtagactgaataggtagtcctggattacattggaacccgtgaagaacttcagaccatttcggcgctgcatgtacttacgtggcattgccttgagggccttgttgaagacgctacggtctacaccattacctccgtggtcgattacgtggcctcctgcgagtgcacgcttgcgccatccatcgaatgcctttaggagtgggtctccagtcagggccgtattaccgttaatggctacgtcctcaaggtcgttaccggcctgagtcgccattagacgtgcaatgtggtcctcaagaccatcaccctcgatgttgtcttcaagggactccgttgagatttcccagtctagacgaagcttcttggtggtgagagaaatcttggagaaggttgctccggcgtttacaccgtcgtcaactgcctctgtggcgactctcattagacgctcaccaattccgagcttgtcaatgtcaactgtgtcagctctcatacggatggttcgaacctggctaccgagtacagtggcttcccacatatagtcgatgaagcggttggactgttctgcgtttagcagaccaccgccaccagatgctacctcagtggtacgaatgaccttttcaattagctcatcgctcatgttgtttgttcacctctttccttttcttagattacttgatgtcggaaacactgaggaagtgtccgccccactttgagcccttagccttgctaacggtctcttcctttgacgtgccaacgtcgccggactttctgatagcagtctccttctcaacaccgtcaagtctcttagtgacttcctcgtgctggtcggatagactgtcaaacttcttgctaagctcgccgtgcttttcgactagctcagaaaactttgaatcgaatgccttgttgatttcatcaacctttgactcgatagtcttgatttcctcagcagtagcttcacgagtctgctctaggcccttcttgattgtttcctggacctgctcaagcatcttctcgaagtttggctcctctgcgccaccctcgtctacttctgctgctgtttctccggactcaacagttccatcagcctcaacttctggagcattctcagttgttgattcttcaactggagtctcggattcggcacgaccctgctccgcaacgtcgtcctttcgctcatctgccatttgttctgcacctccttcggtgtcctcggacttagtaaattctgctacaagagcctttacagcttcctgtcgattgctgtcggtctcaaaccaaccaatgttctgcattgcctttccgcaagcggaacagctagcactttcgtcggttgtagtcttagcgatttcatctgctggacaccagaagacattctctacagaggtttctactaccatgcccttcattaccttctcgccaccggccgccttttcaatagagaaaatgttggctagctgattggctggactgtcaacgagggacaattcaataagttcgtagtccttgatgaatcgtacgtttgtctgggcatccttgttccactcagtgtcagagtctacgatgctaccgccgatactaaaaccgcttagagtaccgtcaagtaccttttcccatgtatcctgagcaccctttgagacatacgctgtagcgaaaatgcctcggtagaacttctgagtggtggagtcaaagaactcttcttccctgaagtctactagctttcctactgcaataggctgatgcatttcacggatgtttccacggaaacgagcaaaagccttctgtgaagcctctgctagaacaatgtcaccatgagagtcgaagttgtcaagagtggcaaatccacttacctgacgcttctctacatcatactttgcaatcggcattgtcagacgaacagaatgttcatcggaatgccaggatgccttctcaatcttcatggtcttatcttagttacttctttattatcatgcaaactagagtcaacaacctttctattgactcgtaggttgatagctacaaaggcgcaatatatggcaaccatgaaggcattgagccatccaactgactgccagcttcccagcaggtagaaaccactgaccacgaaccagtagtagaagcctgccagggcacctctgtgcaatgcgttatatgcttgggtccatacgccatacagcatcattgttccaatgactatggctgccatgccccatgccagttccggcgctagattgttcatcatttcataaattgggcttctgatgaatgtctgccatggcagagccagccacaatccccacatgaaagtataaactcccataatggagattgcggctgtgttgattggctttcgcatgccacgggctatctcgtccgtcttttcccaaattcggtgtgtcacggtgtagctctaccttctcccttcggctgacgcgcttctcctgaagtgtcagcagaattggctgaacgttcagcgtctcgtgctctggtctgaccagtctgagcctttgcatcggctgcttgctgtggcttgagttcaacaatcttgtcgccacctggaagacctggcttaccctgacgtccacggatttcgttaggagtcgtccactgattgcgaattgctcgctcatcaatcttgctctgagtatcctcgtcagtgagtgaaagctcattcagatgaatctggaatacgtcagtgacttccttcataatcttgttcagctttgtttcgaaaatgctctgttctggtcggcatacctgctccttgaacgtcttgtcagcatctcgtgcaactgctaggctggcaccctcagcaagacctaccttggtgatagggactcggtgagccatcagaatgtcattcaggtttcccttacggtaattgttgaatgaactgtcctgagtaccggcctcaactggcttcatttcaaagctggtcttacggtcctgctcgtcaggtggaagaggaacataaagcgttctgtgattcttgcccttcagggaagtctggaagaactcagtgattcgtcgctctgcggctggactgagctttcctcccttgatgacaatgacatacctaggaacagccttgttctcgaaatagtccaggttgaatcttgtggcgaactcattacctgcgacagcagccttagctgccacgatgtcagagattccataatatccatttcctggtgtgtacttcttgacgtggatgacctcattaggtcttgtgtctcctcccactgggtccggcgtcttgtcgtctccgaagttacggaagaatacagccttgtttgagataatctggacaaagccgtctctgcgctgtcggatacgcatggtggtactagggatatgacctagataaccaatttcgccatttgtcttccggccgatttcaagatagccattgccggttacctcgtagtcagtccataccttgataagtgtttccaagaagtcgtcttcctcattgcagctctgaatccactccatgagtcgttccttttccactgcaatcttcttgcgcgccttctttaccttgtcttctccctcaatctcatcaagctttcgcttggctgctggagtctccacaaggtcatagcctagaccaacaatgttggacacctttgcctttacggctgcatagtgaggtgaagaaacctcgtacagcttggccaggtaatcgagattgtatggaggaagcattacctggaaggcgttgtaacctgtgacctcctggctttcttccttctttgaggcagcgccatctgtaccacggaagaacttattgatttgcttcgtggtgcgaatcttgaaggaacgggaaaggccatcgaggttcttgttcagctcatctgctgatttctggaatgggtcagggtcgtgactgatatcacgactgtattctgagccaagtgaaacttcaatgacgtgttcgtcttctggctctgccgctacaactcgattacttgtcatttctctccttgtatgcctgctcgcttacgagggcagggatatcgtattcgtctggaatcagaccgaaagcctgacgctgcttctggtattcatactcttcatcggtaatctttctgttaccggccaggaatagcggctctccaaccatgattccgtagtgacgaacggcctcagtcaattcatttatgcgcttgaggtcacccttcattgcagaaatgcacaaccagttgttgtcgtcatctcctacaaggcgaccatcgggcatctgccacacataaacaccataaccggcctcgtctactactcgcattctttgcttattcatactgatatcataaggacatcgctattaaaaagcaaaatccaccagtacatcaatcaattaagactgatgcctggtggatttctgacatacatcgcagctctttccatcaattcaggttcgtctcttagaagaccgattgccctgttgcataggccacacaaaattccaaagctcccattgggagccaatttgctatcctgcgggtgtaattccccagacatgggcatacaaattggttgcaggcacggcttctgctatttcgacagcagtagtatctgtcaggacaaggccaggcagtccgaggtaggctgcatatagctgctgcaatcctgtcaatgtcaatgcattggtgataactgatagctgacttacaagagtgttagtaacaccgatgttcagtgccgggttcgctgctgtggtgaagattcctgcaatgtgataccagctattggaattgaatacagtcgctccactggtcactgatactccattgactgtcagagtgctcagacctgagaaggtaatggtattgccagacctggctacactgatgccagttcctgaaaggatatttcctgccaccggctttacccacatttcgaaaccagtgatgttcaggtcaccggcctcttcttcgccattgtatgaattgtcagcagtcagggcgatggcaccagacaatagctgaatcccattgaagtctgcatactcgatgggttcgaaatactcctcgcttgccaggccatttcccgtcagggatgcgaacctgtcagctcttgtgccgacgaacgtcttatcgatgtatactacaatatttagagagcctacatagcttgtgtcatccacaattcccccgtcaaagctgacacggatatcaatgacatctgcattgtcgagtgattctgtagggcctaggtttggattcttccatgtcgttccgtcatcgagtgagtattcaactgtaaagtctccttctccgaaccaggtgagccgcatgtctgccagcgttgattctgggacttctcccaatgatatgctggtctgccaaacgccaggcagagaagtgttcgtatatacaggaacaataagaccatcctctaccacctctgattctgattgtgtgtatgaaggaacaatgctgtcatttgacactgctacatctgtgagaacacctgacttccagtcttcctcaagagtccactccttcgccgccgcgatatttctcttttcatctgagaaattccagaaggtggcattgtgcattcctgcaatcgcttcacttgtgtctacagaaactccatagctgtagtgcttggaaattacatcaccagaaatggatgttgaatagattgcaggagcatcaagagcaatggtgcttgacgtcgctgactgacctccgataaggtctgtcgtcaggaatgaataagcatctgcctgctggtcctcagtcaaatctgtttctgccaccagcttgccgtcaatgtacaatgaattctttgcatttgtatgaactgctacgacatggaatgctttaccagtctcatattcatgagaggcttcacacgtaccggccgtcagatactttgttctgaaatagatatgtgtaggaccaatgacaagaccgtcgtatgcgcttgaatgggacatgatggcgacttcgcctgtgacactgatgggcttcacccatgcctctagagcgaactgacgcagctcgtagccattgttaaaaacagggtcgtccatatccaggtggtttgtattactcaatacgagagcgttgcctgagcctgctacaagggcagggtgacgaacgattgttcccacgaggtcagcggtcctcatgcttccggctgaatccgggaaggctggacctgtctcatccagcttccaatagctgaatggattatctgcgagaacttgtagttgataggacatgttcttattttatcatgcaacacgcaaaaggccgagtccgaagacccggccaatgcggtaattataatgattccatcctaagccagcgcgcacgaagacgcacagacaacccggactcatttcctaggctgtaagctcagtatatcacttggcgttcaaagcgtcaatcttcttgttaagctcgtcaaccttctttgtaagagcctctaccttttcgtagacgcctcgcagaattgacattggagcccaagaaggattgtcggttgtctcgtgaccctttggcggtgttgctgcatctaggtcccagacagacttgtaagtagcatcctttgctgccacctttccacctcctgttgttggaggcttgtccgtcgttggtggagttgcgttaggaccagccttcagagttgcggcaatatcctttcgaactgttgccatattcataatcttgccagaagcgtaacctgggtcccacttgtcgtttgaccattctccgtgaccgatgcaggaaagctcagtccacttgtggtgctcaagaatagcagcggaaagcttgagagcagtcttgtactgtgcatctgtcatctcgtgagagccagagtacatgatttctacgccatagaaatggtcgttaccgtccacaccgttagcgttaccctttgtaggatgaagctgccctgagtagtcttcgttgattacgtggttcagaacctctgggtcacctccacctgcgtggtttgcgcgtccccaaccaactagataaactacaccatctgttgccagaccaaactgacaaagtggtcctggtagattgctaagtccgttgtacagagttccacctgcatattctgctgcgctaccggctgaaacgtcagcaccagtgtgatgccagataaaaccgtttacaggtccgaaaggcttaccagttgcagagtctctgttgtgtgattcccaggactttacttccttgtacttaattccccacctcttgagctgagctacaatctgtgcggaagtcattggtgttgccatgaaataatttcacctcctcggttagtagtataaatccgataggtataaaaagcaagaagcccggcaatcatgccgggcctcgctttaaccaatatctactacttcacaggctcctgctgaacaggcgagttcctgagaaccagttgtattgtcgccttcctcgtatgttggaagaaggtcccactcaatcttttcaggcatcttctccagccatgtcttatattcggcctcagtaatcgtctgatatggggcctgcttgtaagtgtgctctgagaatggcaggaatgaaattccggacacctcatcgaagtgcttgtaaacccaagctcctacttccagccattcgtcttccttgactgaaacagtgatgcttggcttgtgctcacaccagtgtcgctgatatgcaagccagatttccaggtgctcgatggccgtaaggtcatttctagtaagcgcaccttctgccgcccgcttggggaagctgaatacggtggtgtcatttggcttcattacgtctggctcgtttggaactccactgtccttcaggaactgagtcagagggtccttgttgtctcctcgtaccgttctgatgtaaaagtggtcatgctctgtgtgcattccagaaggaacgccgacaagctgactgacagttccagatggcttgacacaagtgatagcagcagagcgaggaatgccgacttcgtcagcaattcttgcattgacagcaactgctctgttcttcagagattcaagaacatctgcaagctccttcagaccttcctggcctgacatcagtctgttaccgaactgacctgtcaaagaaacaccgagtagtctttcttcctctgtgttcttcttccagaccttacgcaggtacttgaagtttgtcagagttgactgccatgttcccagaattgtcgctgcgctgacctttcgcatcagagtttcctctgtgtcttccggtcgcacaacaacttcagtcaggttacagaactgattaggtcgaaggataatctcactgcacggattggttccgaagtcgaaattaccgtctcgccttccattcttagctgcctggctctttgctgcctgtcgattgaagattcctctttcgccagacttgctgtcgtaaagattcttccattcagccatgaatgctgtcatgtcaggcttgcttgtgtaagctactgagttgttggcaagggcacggtgaccagtgttttcccaccagctaccggactttgctgtagccatgcgaaggtcagagaggtcagaaagagaaataagcgctgaacgccttactccaccgacaactacaacttctgcaatcttacagacaaggtcatgagcctcaagggaggtcaactgcctaccggccgcctttgtgaagacgtcaatggtgaactggaaaagctgttccaatggtcctggaccagaggcccggccaccaaaagtctttagtctagcgcctgctggacgtaccttagaggtatcccagcgtggtactcgtccaccccatagaagacttagcagttcacgataagagcgtgcccagccttccttgctgtctcctaccttgattactgtctcagtcgcctcgaactcttctgcaataacaggaagctttcgaacgtacttctcttcgacagagaatccgactccagtaccattcatcagaatgtaaagcacttcatcgaatgctcttgggtcgtcaatggtgacataggaacagttgtaagaggctacgttgtctctgtccagtgccgcgcctgctgtcatcattgcgcgcatgctaggcattgctgcctgctcaagaataaattcccgaaccagtgaataatctccatcgggaacatcgtaatcgtgattcttcttcagactgttgcgcatgtattccagatagcggtcaacagtttccacatatgtttcgcgtcggccaaggtcatcacgataccttgcgtaacggctagtgtggataaaattcctgtaagggtctgtcaagaatcccttatcatcaagtagcaaaacaaaatcctttcaagccctgctgtcgaggcagggctaatcatcgcgtagttcctcaatggtacagcaaacgcccacctaaatcaagtgggcgaatgccttctctgtgagtttttcccagttgtattcagcgtgcaactgaggtgcctgagcgtagaattggtcgctcaatgcatcgaaattctcgtatgcataacggtacttatccacaaggtcatcgaagctcggttcgagcatcttgcctgggtgcgtctcgggccatggtgacggtgtgagctttgaagaaatgccgagtggtcccagatagctcttgtattccgcccatttctcagtgcagattgtgggcattcctgtggccagtgcctggaacggaatgaggccgaaaccttctccccagctcggatagaccatcacgtgatactgcttgacaaagcctacaagatggtcttctggaagctcttgtgtgaccagcttcacattgttataatctgtgactggtccaaggattctgccgttctgacgtcgtctgacattgttgagtctgtgagctttgatagtcagctctacgtcttctctgtctccgaatgccgcccgaaatgcatcgactgccatctggccacccttgcgaggagcaggctctcccatgtgaaggaatcgaagcttctcgaacttgtatctcttcttaggagtccactgagggtcgataccatgtgggtatactcttacattcttcacaccggccgctgtgtaccagcgtctgatgatttcggatgtcgtccacgtctcattagcaagattcatctgctcaagccagcccggtggcaattgggttgactcccatggcgtgtagccgatatgatagttgaactgattgctccactcccagtaatctggctgagagaagaagatttcgattgggcaagtcttgtcagcgaaggggactctgtgtcctagcttctgtagactgcgaaccatgttgaatccggccacgccgtatccgacactcgtgttcaaattgcccggcaccgttgaaaaactgatgtccacagtcttcctttcgtcgttgacatgctctttgagctatgctacgattatatcagttggttgagctatgactcaagccaacagattttgacacagtctgggaactgtggtacggtttatataacgtactgctcgacccagtatgtgggagccaaatctttggtaatgccccttagcagggtcgagctgctaaggggcattttcatatgcccaaaaatccgactatcagtcgagggattcttcgggtcttgccttcctctgcggtcactgcttccagtttcgacctggtggggtgtgtaagacccgttatttcataacgggactatataaattaactactaacatctaaagatgttagtagatggagttactacgtaactccataaacataactactaattcttaacaaggaagaagtataagtatggatactaatctctattatcttagatactatctggatggtaagtttagagaaactaatgactatgtaagtcatcctgtagccttgatggagaagtctgagcttgagtctgatggtgccaagaacgttgaaatcatcaagtatgaaggtctgtcactcttcaactaacctgcatagttgacactgaacgaacgaccatgtagagtcttaactcatgaccgggtggcagttacccaactgctgctcactacttggaggttaatactaatgggtcaagatatgaggggcaacatagcctctgctggtctcatccttggagtcgcgacagcagtatttgctacagcagcttcagagactggcagctctcctgtagagcaggctgttaccgcttcaacatcgccagaagcacaaccaagcaagagtgaggctccgaaggctgtgaaccagcctagtctgacatcttcaccttctttgaaggttacaaccattagcatcagtcctgtggcatactcaaagtcaattgcgagtacaaaggattatgctaaggggaagctcaagaagaagtttcctcgttcctggaccaagcagtggaactgcctgaatcctctttggattaaggaatcatcttggaactacaaggcgaagaatccttattctgccgcctatggaattccccaggcgaaccctggtagcaagatgaagtctgctggaagggattggaggaccaatcctcgcactcaggtaaagtggggtctggacaagtacatctacagtcgctatggaactccttgtaccgcatggagtcatttcaagcgtcatggctggtactgagtcttgacaaacatgctatgcttcttgtatgaaatctgatgaggagcgttgcaaagaatatggcgttccatatgaccccaccattaccaggccggtcgtttatcagcagtcaggatggaagtgtcacatctgtggcaaaagggtgagacgaactctcaaatacccacatccaaagtctgcttctctcgaccatatcgttcctctttcatggaggaaggattcacctggccatgtgtggggaaatgtggctctcgcccacttgagatgcaaccagagtaagggtgccagatatgctggctctacaaagcctgctccaaggcgtccaagccgtgttacgcctttgtggaaaatgcggctggcaatatatgcgggtactggtattgcattttatgtaggagcgacagcgccggtattgactgtgtgtgccgtcttgtgtatactgactgtaacaccaagaaaatcccgtcgtcgtcgtagagccaggtggaggcgttaagtccaccattgccccgttagttctaatggtaaaacacatctttcgtaatgatgagttgtcagttcgattctgacacggggctcatgacaaagctaggaagggtcgcactcgtagttgctacagtgctgtttacaatgtccttcattctagttccttctgtccttggcggactgacagcagccttctttggcttcgcttgtttttccgagttctggcagaatcgtaactgaatgagatacacgatattcagcatcgactacagtcgactcaattacattcaaaaaatgctcccacagctagaaggctgggagcctattcgtgttcctgctgtatatggagcagacccaactgagctgcattacgctcaattgagacatccttacaaaatcaactggcccgctcgcgtgggccatcttggtatctggtacagcgtactcaatgcacttgataaggcaccgattgtaacctttgaagatgatgcattgctcagtgacgattttcaattgaattttgaacaacgtatcgctgaacttccagaagactgggatttcttcagtctgtttcttccaagagacagcgacaagatgtttgatgagcgtcgagacggagtcagcaggagtctttgcaaggtttaccagagatatggtggcgtctcaatgttctattcagagcagggcgcggaaaagattaggtccctactgaagagggatggttttactggtcagtacgatgatactctgtacgcgtatgcaaaggccggtgagctaaatggatattgctcaaagccttcatggactgaccttgtatatatcacaggtcttgaaaagagcatcgtacaggagactgattacgcatgattgaagaaaagaagattatggttatcgttcctacaagagggcgaccagacaatgccatgagattgcttgaagcaattgagactacggcagaagtcgatgtggttttctgtgtagactatgatgaccctaagctagaagactattacgctacgagattgccacttcataccggccgtagaaggcgattggtaggaacgctaaactctgtagcagagaattacgtggattactacgacattatcggattcatcggtgatgatgtaattcctcacacacatcgctgggatgtggaaatcaccaatcatttccagcgcaacatgattgcttacgccaatgacggctggcagggagaaggtcttcctacggctgtcttcatggattcagcaattatcagaaagcttgggtacatggtgagcccaactcttattcatctatttgcagacaactactggaaggctctgggacaggcactgggaaccctgacataccttgaccacgtgaacatggagcacatgcatccatttgcaggcaaggcgtcagacgacaatacctaccaggaagccaattccggagaagtctggcagcatgacgagctttcattcaagtattacgttcagaaccagctagctatcgatgtggagcatttgaatgcctaacatctatactggaggaacgttcgacctgatgcatgaaggtcatatcgaattgctgagaagctgcaagcgtcttgccggtgagggcaaggttgttgtatccttgaacactgatgaattcatttctcgattcaagagcggtccaccagtacagtctttcagagagcgtaagctgatgttggagtcctgtcgctatgtggaccttgtaattcctaatatcggtgaagaggattcaaagctcagcatcatcgaggcttgcagaatcttgcacttgatggatgaagacctgattgaagtgattgcgattggctcagattgggctggccgagattactatggtcagatgcagttcaccaaggagtggcttgacgagaacgacctcattctgatatacatcgatagacgaacaggtatgtcaaccacgaaaatcaaggagaagcttcgtgagggctgaagatgaactgatggcattgatagaggcggccggaatcaatagaccgcttgccattgctctcatcacagagataaagcaagatgcagtagaagcagacaacgcattcgagaacgaagtgagcaatcttcgtaacgctatcgaggatgtcacacagaatgtagagtcagctctgcgcacattgcgcgatgcatgatagactgattgcatgtggcgcgattttcatcagtacatgagattggtaaaagaagaaaaagccccggcgctggaatgtccagactgtgggtataacttaatagttagattgaagcctggtgcatctcccgacctaagactatggtgccctgtatgtgatacatatatcaaacctggactagagatgcatcataaagtgaaaaatgcggttgatagggcaggcattgacgaacggtccgctctttgatatgatgaacatcaacaaaataactgaatagtaggcggtcagaaatggccgcctacatttttgtctaaggagaaggaagcaaatgccagatttcaagaaggttcttgactggcgaggttccatttcattcggtgttgttcttattgttgccctggccttgagttggtggtctctgtactcccttgctgttacgttctatggagtaccgcagatacttgctattggtgtcagcgcggcattcgacggagcagcactgtttgtagctgaccttgccagcaagtatgcacgaacagaagactcaggtcttgcaacaaagctagcgacgtatctcttcgtgggagcgtctgtatacctcaacgtagagcatgccatgttgctgtcctatggggtccctggaatggttctttttggagcaccaccagttattgccggaatccttttcgagctttatctgcgcttcgtacaccgctcagagatgagggcaaacgggctcgttccgaagcgtatgccagtgtttggaaagattagttggcttatcttccctggcaagactttcggtggcttcaaggatgtggtattcttcaggctgaacgaggttgtcacgggtgtcactggagagcctgtccagaggacaaggagtcgtgacaagaggacaaagtcacgtgacacacaggacaagcctcgtgacatgtcacaggacatgtccccagatgtgacattcagcgtgacaacaccggacattccggtgacagataatgtcccagaggacatgacagtgtcacctttgtccgggacagatgacataacgaaggacaagtcagtgtccgccctggtcagagccctatgggcaacaggaatcacggacagaacggaattgcacaagaagatttgtgacatcaaggggacaacggttcctgtcaacacggtgaacaaggcggtctcacgtcttgacaatgtccccggcgcgtgacagactaagtgacatgaaggacaacctagaagacctcgtggacaagtggcatgacagtgaaagtaaagtgtcactccacgagtttctaggaatgacacgattggagtacgcagtctgggtagagtttgacatcaagcccggtgatgcgattcctctcgaaacattcctgagactcaggtcagacgcataccggtgggccaatgatgccgaccgctacctggaagagaacaagcggtacaaagaagcagtctctgaacttcgggcggtagccgaagaagactggataaccgatatacttgacaggaataacgtatgagcatacccaagaacatagatgaagctgaaagtcgtcttcttctgctcaagcgtacgttgcttcaaacaagtgataggctgaagcagaacagactgaagagagaaatattcgctctggaagaatggattgtagctcatgacagaggcaagcgacgttagaaaggctctgaataacctgtacgagcagcgtcggaacgctgctcgtggtatggaactggcaaaaagggccggtaacagaattgcagagcagagattcaagcgtgaagtcacccgacttgacggagaaatcaagaacttggagaactgatgtacaacgttcctcgtgtaggtgtcagcattcttgtcatcatggagaagtcactcattcttcttggacgtcgtaagggcgctcatggtgaaggtgaatgggctttgcccggtggcaagcaggaattcggagagtcaatcttcgatacctgtgaccgtgaactgatggaagaatgcggcccggcactgaaggtgaacaacgaacgtgttctctgtgtaggtgaccttactgcatatgaaggtaagcacttccttgacgtcaccattgcttgcacgtatgtgcgtggcgatgccgtggtgatggaacctgacaagtgcatggaatggcagtggttcaacatgggtcatctccctacgccactgttcgcaagcgtcaaggacattgtcaatgcccatctgtacggcttcaattactggattcggtaacgaagagaatgactaatcaggaactcattgacctgaagaagttgcttatcgggctggtgactgctatcctggatacacgagacaaggggatgagctggggcgagaagcagtttctcaagaggtccctcatcctgaaaatcaataagctataggagtagagttgaagattgacgtattggaccatggctatgtacgactggtagaccacatggggtccgacctctctgtcaactaaatattatgaagagatataagtatatacaccgtgcagcccttacaattcaagtgcaagaggcaacaacggttatccaggaggactgggaagagatagtttctatctctatagaccctaacgacgcaaactacgttcacatcttctcacgtgttgagcttacagatgatgaacatgaaggatatgtgcgagacgcagaggttcatttccagggctttaagtgatactatgggcattcaatttgacatttgaatgcccatatgtcattatatgagtatggaatgcaaaagctgtactctcgataagccagaggaggacttcgccccaagacgggagaagtatcgtactggtaaaagacgcactacatgtcgtctatgcgttgctgaacagcagcgtgagcgctatgaaagacataagcgtaattcatggttcaagctcaaagctagtagagcgaggagccgttctcaacatctcagagtgccatgtgacctcgatgcagagtatttggaatcaatctggacgggattttgcccggtactagggattcctttggataagcttgctgatagaacaagtgaagaggctgccgaacttgataggtttattccagaaaagggatacgtcaagggcaacgtcacattcatcagtcgaaaagtaaatagacttaagaacaacgcctcaattgaggagctagaaagactcatagattggatgaagaagtttgaacgacagaattgatgtactcgaccagggttacgtacgccttgttgacaaaatgggttccgacatttccatcgtgaatgctgcacgtgtttcctacgccaaggaaagtcaggaattcagcgaccgtgacgaaagtctgttgaattacctaattcgcaataatgaatacagcccttttcgtcatgctacgttgacttttgaagtatatgcgccactatttgtggcccggcagtggtttaagtacactgtagccagtacacaccttgatgaccaaatcggctggaacgaatcaagccgtagatatgtgacagaagaaccagtcttccatgttccatcagagtggaggctagcccctgacaacaagaagcaaggctcaggaggcccggcagcaattgagatttcacaagacttccaatggagtctagaagcatatattgcaaagggtatgaacctctacaacgacgccttgaatgcaggactagcgccggaacaagcaaggctattccttcctgcctatggtatgtacgtcagatggagatggacaacaagcctggcagcagtaatgcatttcctgaacgaacgtcttgagcacaaggcacaattggaaattcagcaatatgcgcaggcagtaagagacctcgtcgttcctcacttccccaaggcgttgaagcaatatgcgagcgagtgaacacgaacatgtttacaagcatgtatatacatcaatggccggtagagaagaagtctatcaatgccaatgtggttcaggatacgtaaagaatctagagacaggacaaacatacaagtgatagcaagagaacttattgaaatactgtcaaagcatcctgaatacgaagtcctcataggaacaagcgatataatcggcccggtgcgagatgtgataaccgattgtttcgacacagaacaaggtatagtacatctgatagaggtagacgatggctgaagaatcagacgggtccgaggagctagctagcgcattcgatgaagacacatcacaagtagtacagactatcatgttgatgcgtatatacgatatgctgactgtcatagcaagagcattgagcccggcagaagcagacgtcatatacaaaggtcatagcataggcaaggtattcggaccagcacctagcttcgatatggcagaagacgaatacggaaatgacgatgtgcagtaaagtagacagtgatataggctatcaccaattcagattctttcaacaggattacgaaatcttgtcaaagaactatctgggattgcctaattgcctgcattgcggtcatgagcctgtagaaatgctgtcatacgacgaatggtgcgatacgcatttggtgcatatcagattccaatcgtgcggccatttggtgggcatcaggactgactatgagtcagataaaatctacaccgaatgttagtaatttcgaatcttgtatgatacatgcttcgcatagttaaaatcgttatcaaatagtgcgcacataacccttgacaatcttgtccggggaaggttccgcagaaggtcccaggccagatgaaaacggtttgcaaaagccctgtgtgccgttctaagcgcatgaaaagcccgccccggcaaaccacccggggcgggtctgtttcgtctctcagacggtcgcagggaccgtccagcgggcacgtcctcggttttccatcggggcggcagcggtcacgcccgggtctgcaagcaacttgccatcccacagctcgcgattgtcgtaaaccggaatcagtacatcggtaagggtttccttacccatatcagcgttgcactgcctacacagcgggaccatgttgcaagggcagtacatgccaccctcagcatccgccaccacgtgccccaggttgaacgtgtccatggcacggggggtgcctcccacataggcacgctcaccgcacccaacgcacgtggcccaggtggcaccgtcgctgtgtcccgtaagggaagccaggtaaagcaccgtggccagaatctgacgccgtgcgcgagcgttggtggccttctcttcacggtaggtgcaggtgcgtccgttggtgtgagtgttcattgggtgcctcccggttggtgtgtcgttgtacctacattctcccctgcccggcacgggccaaaccaccaaaccgaggaatttctaggtaacagttctgtaactgtccggccccgacaatcgggcatatagggcaaaacggacattgggtacatagggtacataccctaccatacctacccatactggtacatatagggcatatggtacagatggggcatataggggcatagtgggacaaagctcttttaaggggcctttaagtacatcccgggacatatcccccgcgcgtgtacgatagggcccaaaattcccggagatgtagagcttgcatctaccgtatccatgcactagactggtactacaccgagagagggagtacacaccatggaccttcgtgaccttcttgagtacgccgcagatgcgggtgttctgacgagcatcacccttcccgtgagcgacgtacgggatgcggaccgtgtggacggcttcgaccacgacgcctacccgtacgcgtccgaactggcctaatcacagagaagggtgcgggatatgcggaaaaacaagaatgcaaagcgtttgacccactcctgcacccttcttgtggagcgtgagggttttcgttcggaaacgggcgttttcacaaccctcattctcggctcgatgattgagaaagcccaaaaggccgggatgtcgattatcggaactcggcattcgaaatactgcctttgtggcgacgggaaaaacgagattattaaattctgaaaaaagtgaaataaattcacccctgttttctaggaattagcttcggcaacctaagaaaacaggggtgaatttttgtccgaattttttgcccgccccggacaatctctccgcatcctgacagacatgttgcatcttcgtttacgggctgctagactggtctcaccgacaaagggagagtgtcatggccaagggtctgcgtatgggtcagcgtcgtcggaacaagcactgggtgaacgtctacgagctggaccgtgacttcggtggacacgaggaaggcggctggtggtacaactacgccatctgtatcgaatcgtggccctgccgctcccgtgagcaggctgagaagctggtcaagtgggccaagactcagcctcgctacaacggctccaaccgtgccatgagcagcgtgaatcaccgcctgggtgacaccgtcgaaattctcatcgaaaaccgtgagggtgccgactggagcgactaccggccttgggagtgatgggagggcccttcggggcctgcccgccccggacagactaaaaatcttgaaaacagggcttgcgactaaaaatctggcctgttagactatgactaagaaatccgagagaaggagtgccaaatgacgaatctggcgaagcccgagcgcgtttcccttggcagccttgcctacgtcggttcattcgactgggaccaggaacagtggcagttcaacgtcactcaggtgtggaagaagacgcgaggacggtactacgtggcaagcgactctggttgttcctgcccgattccgttcgaagacatcaactacaccgacgacaagggtgtatacggaccttacaacaagacggaactcaaggcttactttgagcgtcagctcaaggctgagcgtgggatgcgtcctcagagtgagttgaggcaggaaatcagttctcttctggccaagcttacctgacagaaggggccttcgggcccctgtcgggcccggacattttgaaaaaggcttgtgctggctctgttgagtgtgttagactcgtcttacaccgagggaaggaaacaccatgaaggtcacccgtaacgacgttctggcagccatccgtgaatacgtccgtgtgtggggtgccatgggtgttgagctggaagtcgagtatcaggagggacagccttccgctggtcaggctcaccgactcttcgtcaacaacaaccaggccgctcccggtgtgggtgaccgtggctacatcggatggaccaaggccgaagcctgcgagaccctgtggaccatcgttcgtaccttgtgtgacctgagcaatgctcagcagggagagtgacgggagggcccttcggggccctgcccggcccggacatcgggcaaatcggacattccggtacataatgccagcgcgtttaagggggcttgtaaaagtccccagcaacctgtagactttagctatcagcaagggggaaagacccctagcagagactgaggagttctcatggccgttaccctcgcgaagtccaccgacgccggtttcgtctcctccatcgagctgtccgacaccttcgagatgacggactccaagggacgcaaggcgcagtacagcatccgtctggagacctggcaggtcccgggtgctgcgaagggcaacacggaggtccgcatcgtcatccgtgacatcgagaccggtcagtggcacggtgcgacgaacttcaagcagaacatcatgctcgacgtgacggccctgttgaacggcacgcactccaacaagcgcaagaagtaatctcaacccacggggcccttcggggccctggcgggcccggacaaccggcaaaacggacatattgcagtcttgcatctgtcggaaccgtcctgtagacttagaacatcgaaagggaggaaaacctcccaacgagaggagctgtcatgggcgttcgactggcgaagaacatcgaaaagcttccggaggacgagccgaagacgggagtcatccccaagggctggctgtcggcaatctggacggtcgaagagagttcgcctgacgcgcacctggacgacgtcgagattctggttgactgcgagtaagtcagcctgctagactcagtacagcacaaggggacaggccaacggggtctcgctctacgagatttttcccttcctgccaaagtctctgataactgaataccccagcttacttctagcagtggagttcatatgctcgccggtatcgttcccctctctcagtctctgtccctccgtgtggcgctcatcgtccagggagtcgacgtggagttcggtgactcggcccgtggctaccagcgtttcgacatcttcgacgctgacatgacgcgcacgcgtgacatcctcggccggttcccccgtgtggtggtcggtcaggccaagcgcaaggttcagtccccggatgcttcccttctgaagcgtcctcagctcatctcctccaaggagagtgactaacagattggtcggaggccccttcgggggccttttggccgccccgggcaattttggcccggcactagacatagcccttgttggcagtgtagactagaaccatcgaacatgggaggtcatcatgcgtcaggtatctgttgccaagctgattgagtcggctgtcttcaacgatgctgcaacgcgtgacgagacgactatgcaaacgatggaatggaagttcgatgaactgttcgagggcaacccggagttcattgagtctgttcagaagcacggtattcagtctgccatcatctattcggtaagcgagaacactgtatatgctggacatcaccgtgtcctgtgtgcatggctcttgaatatcgaatacattgacttctttgatggtattgagggcgaagactctcaaggccgtgatgagtgggaaatggcagatgccggatggcctactctgttcccggaagaggaataatgatttacttcgtcatgcaactgcacttctacctgtacactcatcagaagctacaggaagacctctggctcattctctggtttatcggagtgggtctgcttatcgccctctgaccaccgggcccttcggggcccgcgtcgggcccggacaacgggcaaaacggacataatctagtgcttgcacagagccgtatccatgctgtagacttaggatatcgaaagggggaaacaccccctagagaaggagattcaccatgcagctcgtcctcgacaccatcgcccgttccttcgccaacaccccccttcgcgcctctgttcgtcagtcgctggtcgaagcggccatggagggtctggacctcgaagcggctcgccggttctacgacattgcgttcgagcagggtgagttcgccccttccgactccgagtgagtctagtctaaccatcagcccctcttcggaggggcttttggcggccccggacagattgagaatgtcactggactctgttagactgtacccatgaacgaaacgcgcagggccttttcagcagtcgagcgtggcgagcgtatccgttttgacaacggttactacatgactggccacatcactcgtaacatcggtacatacaacggcaagcgcatggctcacttcttcgatgaggcaatgcgtaaggagcgaattatctcctacgctgctcaccgttcagtcgtcatgattcctgagtgcctgtgttgtacctttggcaatcatgacatgccctgtgaatgtgatggcaaggaatgctgtcaccccgagaatcactagcggaagggccttcgggcctgtccgccccggacaacgggcaaaacggacatgttggacatacagcccatttaaggggcttgtttaagctgtccggaactggtagactcgacatacgtcaacaggaagggccaccatgtctgctctcaagcgttcgcacgaccgcaagactgccaaccgtgccaacaaggcaggcgaccagtcagcattgaagaatgcgttttcccttcccagtggaacggactattcctgccctggcgcaacatcggtgtgtgacactgtgtgctatgccggtaagctggaaaggcagtatcccgcctaccgggcccttggcctgcacaactgggaattgctcaaggatgcgtcgtatggcgtaatgcttcacttgctccatgagatgattgaagagttcgaagcggaatgcgagcgattcaacgttcctaagctgtttcgctggcacgctgatggcgacatcttctgcgccgactacgccattgcaatcaatcaggtggtatacaaccaccccgacatccaattctggatttacactcggtcattcgagtacgtggaatgcatcgctggtctgcctaacctgtctgtctatctgagtgtggacagtgaaaacagggaaagggccatggaaacgtacaatgaatttcctggcatggtcaagctggcattccttgccgagactcacgaaatcggcaaagatgtgttggtcgaagaaaccggcaggcgtggcgttatctgccctgagaatgcaaagaccattcctctcatcactgaaaagtctggcgcatgtgtgacatgtggcctttgcattttcggtcgtgaccatgttcgctttgcatctaaggtgcctaagcgtcgaaagaagaccgcctaaagccactggcccccgtttgggggccttttggccgggtcgggcaaccgacatttagggcgaatcggacatataggctgtagacacaactcctgtccgtcctgtagacttagaacatcaccgagggaaggaacccaccatgaccatgacccgtcgcgcctacgccgtttcgcttggccttgccaaggatgcccgtggtcgcatgtccgcaaaggcgtatgaggcgattgcctccgctgagaagtccggcatggtgttcgcagactccgaggctcccgcgcgtgccaaggtcgtcaaggcggcaccgaaggcaggtcagttcgatgccaaggtcgtccgcgcgtgggccgcttccaagggtctgagcgtcaacgcccgtggtcgtctgtcggctgaggtgctggccgcttacaaggctgacaacccggaagtcaagcccgcagagccgggtgtgcacgtcaaggtgaccggtaaggacgttcgtccgcacgctgccccgacccgtggcagtcgcacagagtacgtggcttggttcggcaacaagcgcatcgtcctcggtgagcgtgaggtgtgcaagtgcggttacagcctgtctcactgccactgcggtacgccgttcgtgctcggaatggacgtcgaggttcacgcacgataggatagagacatgtcagtcatcatcgaaatcgacacgcccgatgatggcgctcatgacacagcctggcccttcgcagatgcaaccgctgcgttcattgcgaaggtgctgggcttgtctgtgagcgtttcagacggctacggcaccacacacgactatggggaggacagtgaccaaccgtaatgtcttccattggacgcaagtcattgcgcctgacatgtatgagggcgcacggctcgtaacacctaagcgttcatcgtatgagcgtctggcaagcaaaatcattgatgagcttgaccgtcactcattcgaccccaacgcgctcgcctacctcatgactacctaccccgaaccggtacagggtccgctgtttgctcttgtcattgcgtttcttaacgcatgggcaggcaagaccgctattcgttcggaggaggagggccaaatggtggacgaagcaaggttcgtcatcgaaaaaatcatcgaagcgaagggaaatggttcgctggactgacctacagggccccttgacattcgggggccctggcggccccgggcagtttggcagacgtgttgtatccgtctgctgagtctgctaagcttagaacatcgagagggagaaaccctctcaggactggaggccatcatgtcgctgttcacccttgacgactcgcgtctcgtccgcatcttccacgcgaacggtgtgaagatgagcctgaagaaggcgttcgagctggcccgtatcatcgagggcatccacctggagcgtctgaacgaggtggagaacacgtcgttcgaccagggcaagaagaccgccatgttggagctggacaggagggtcgaggacggatacgcgcagggatacgccaagggtctgagtgacgccaagtccgagttggacgaaaacgtcgtcagggccatcgtggcggccaccacctacgccaacgaccgtttcgacagcatcaacctgggtcgtaagctggcctgcatcaagcacatccgacgtgagttcccgttggtgggtctccgtcaggcgaaggagattgtcgagcagtgcttgggtgagggaattggtgtgagcgtactctgacaaagggggccttcgggcccctgtcggggccgggcgatttgaaatagggcttgtacgcaccccctgcgtactgctacacttagaacatcgaaggggggcaaggagtccccaagattggagctgtcatgcacctcggagacatcctcgacatcctgaacgacgacgccggtgacgtcaccctggcacaggccatggaaatggctcgcaacatcatgaggcttcacagctccgctgtcagcgaggccaacagcaacggctacgacgagggctacaagtccggtcacgaggtgggcaagtccctggtgaccatcccggacccggagcactggcagcgtcagcagtgggagcgctgctacgagctggccaagcgccacgcgcgtgagaacgtgtccgcctacgtggccagcatcggtgaggaccggaagattcaggtcatcaagcgggtgcgcgaagagcacggtctgggtctgcgtgacgcgaaggacatcgtggacgcgttccttcaggagctgcgcgaccggcgcgaggaggaggaccggctgaaccaggaggagctttaccgcgcccagcaggaagccaactactccgacgagccgcctttctagtctgctaggctaggcattgattgcccctcttcggaggggctttcgtgtgcccgcccccgccaacgggcaaatcggacatatgcgacatgaagcccggcagagtgggtagacacatccccttgcagactgctagactagagatatcgagagggcgggaagtccgtccttgacccatcatgcctggaggcaaaaatgcacggtcttgagattggttccaagggtcaggtcgcgttcgcttcccgtcaggagcccgcgtggcaccagctcggcacggtgttcgagggcaccctcaccacgcgtgagatgctggacctcgctcacctcagcggctggaatgtccgtctgactgagctgttccgtcagggtcgcacgtcgaagaacctgtttgaggttctgcggaccaacccgttcgacggtcagaccgatgctctcggtgttgtggccgagcggtacaaggtcgttcagaacgaggaactgttcgctttcggtgacggcatcctcgctggcggtggcacgtgggagactgctggttctatcaaggaaggtacgcaggtcttcggttccctgcgcatcggtcgggaaatcgtcatcggtgacgaggacgtgaccaacatgtaccttctggtcaacacctctcacgacggcagcactgccgttcaggcgtccgtcactccggtccgtgtggtgtgccagaacacgctgaactttgccctccgtggcaacgtcaagcagcagttcaagatgcgccacacgcagaccatcgagggccgcatggcgcaggcccgtgaggctctgaagctcacgttcgcctacgcggacgagttccagcgtgagatggacgagctgttcaaggtcagtgtgaccaaggacaagtttgacgagattgtcgggtacctgtaccccaagcccgagaaggatgtcaagggcagcatggtcaagtgggagtccaagcgtgacacgctcatgggcatcttcactgacacggctgacggtcccaagactactcagtctctggccggtacggctgcgggtgcgctcaacgcactgacggagcgtatcgactggtaccgcatgccgcgcagtggcaacgtcgacaacctgttcatcgcggccagtggcttcgatgcggtggtcaacgccgagaagaaccgcatccgtaagggtgtgcgcaaggttctggccctcgctgtctaaagggtaaaagaccagagtcccccaggcccggaagggtttgggggatttttggcgggcccggacaaaagcccggcagggtagacagacgtaccttgcttcctgtagactctaactatgaagccggtaagggaaagcatcgaactgtccatgctcgacatcgttctgtctggcagcttctacttccgagtggagggaatcactcctaacagtttcatcggcaaggtctggcacgaagagcacaagtcatggggaagggcagtcagtatccccgttccctctcccacgcacacggcctacctcttggaagactgggagatggagcgcctgtttccttccgagatgtgatgttgacaaggtcgtcagcgcttgctagactagaacagtagtacaaagtgtgtccccaggggccctatcagtcgagtctggcgagggcttaaaaagaggaaggtcggcctctccctggggacctttcaattctccgaggcacgaagtgctgtccgccccgggcagtttcgttagcctaactaacatcatgactctcaacatcaagaggcatttgtccgagatgtccgaatttttcccctttacgatggggcctgaaattcccggaacattcgcctttaagattggggccagaattccggaagtctacaagttttttgaaatatttttggacataaaattaagccacatccgtcagggtgtggcttttctctatatacacagacaataaacaatcatggttcatagattgattctggcccggcagtcataagtgcccttgctagctatatacaatacactactacctactatatggatacaaggaagtctctatgaactaccggccaaatccatggcatcatagtcagtcgtagcagtctattggttgtgtatatagggagaatcgacactcgtagcatatatacattggcatgcataactatgcatggcagatttcagtgacttcggggcatgattgatagattttcgtggctaaatgactccctgttcggggcccctttacgaatgactctaaaattccggagattcggggcattttcagggcgaaatgtccgatttcggggcaaaatatcgtgatttacccttgttttggatgattttggggcataattatgaggcgattcggggctccaatgcatgtcagaccttgttctctatgcatacatatacgatgggccgcataaatacccggactcttacacactatgtttgtacaactatattgcgcacaactgaattatgtgcgtccacaatagttattgagcacagacaacatcaacatgtgcataacttaatgcatatgccaacacttatccacatacctatacagtcatcagcattcttacgatacatcatcatgtttacgatactgacagacagcctcatgtttacgatagtacattcaagcacactgccagtctatgtctattgattcaagcagatgcatctagtatggctctctatgccttgtagtgctttgtgtatatagtagtaggtgtatttacctacggtgttaaaggatattgtatataggggttacagacctcttcactgccgggccttttcgctgcgctggcagtcggccatgccgactaaaaattcatgactaaaaaatcatgaaaacaacggaggactaaaaaattcagacttcagtttgaccaaaacaaaaaggaccagccatcatctggccagtcctcttcatcatcccactcgtcagtccagtgaatctcgccgaaccacatctcgaagccaggattcattcttagttgtacatccatcattgtccttgcaatctccgaagcgccctgcccatgctagccagtctgtgttctcaagggtacagcataggaaacattccttctgtactcttactgacaagactgcatctatatggtctgcttcgtgatacttacgtgctttaaggtatttctttctctgccgggccaaaatataccaggctagtttctctagcattcattcccctttgaatgctgactcatcgaggtcttcaatggtctcgtcgccagggatttcaaattcgaatcgactgcttacgtttgcctcattgaagctgtctctgacgaactcagctttgaattcattaccgtagcggactacaaccttgcctacctttgtatcagtaatggcccagttgtagccctccacacttcggccccgtttaagaggctccttgacaatttcgtatctcataatgctcttatgctaaggcatacattacgagattgtcaatcgcgaacctctggaccaccgttcaaacgctctaggtcttgtctgactcgactgttgcattcttctgaaccgcaaaaggctgcgtgaaagtcaatatccacacggctagttggtcgctgacagatgaagcatacgtgagggtcatcagaatgaaatccagaagggtcataccagtaggttccataactatcccacttgggatactctgagtgtcggatatcaattgccatttcttctcctgactgtgcagccaatcctctcattgcactaccggccgcattggcaggcacatgcaatgagttaaaagctctgatggctcgctggaagttttcaacaacttcatcccagtagtctgcatccctgatggattcgacgtgagactcaggagattcccattcattctcctgcgagttttgcacgattaccacaatcctctagaatcttgaggatgtctgcaagagtacgcttagggtcgttgttccagtagatgaagttatacttctgcgcatcaccattcttgcgccggacctcgtcgtgcaggtattgagcagcaatctgcaagtcatcgttgtgagcatgacccttgagattgatgtacaccatagcaccacgaagacacatccggccctcagtgtctctcaggactccttgtgtccagcccatcttccaaaggctctgtcgcattgcgaaaagaatctctgagggcgtgagctgccccttctttcttttaattctaccgacccttacagagggcactgagcccatgccagggtcgcctttgctcttgttcttcgccaggtcctttacgtaaccgtcgacaagacccttgaatctgctagacacctcagtgtcaggcaggtcacgcatcctgcgaatgtcgctgataactgtcatcacttctccattactgaggcacgatagaatgcgtgcagtgggatgatatcaggctctgacttgtttatgtagtggatatacagacagtcattcttgattccatgaatacttacatggacatactcattgtacttggtgagttccgggtctgtatacacaacgagagtatagtctctcaatgcagctccttgatttgcagaacattattcatgttcacttcgattcgcttgtcatagaaaccaaagagaactctacccctgtcgtcttctgcataacgagtagtgtaacgaatctcttccttgtgaccaccaacataggtaatctcaatgcgctttaccatatgctttggtattccttccacatgtagtagcctcttagttccatgagaatagagccaaggacattcaaaccagctccatcgcaccggccccagaaacggtcattatgctgattagactcaacaagcagagcaacaccagtttcaataagcttcaggcgcaggtcgtcatgctggtcgaactttgccttgactacctctcgcatacagcgtaccttgatggactcccacttgtcaacgtctatgctcagacgcttgacaagcttcttagactgactagggttcgcctgaatcatgtcttcaaggaattcgtactgtgttcgctggtcccccaatgctgccttgtacttacaaccttggaatgcagcctctgtactaccgaatgtcagattacctagcttgactggtacctcgtagaagttggacagaaatttgtactcaccttcgaacttcactatcgggtttaccaacaatgttctgtcgcctctcactcgtcacgtagacccacctacacattacaataattagaagggcccatacgagccctcctatgataaaaaacatttcacatccatccaaagaaccaagccacacggtgtggcaggcagcagaagaacaatctcttcaccatcgtatcaaaggctatgctgtcgtcacacgtggaaccacattgttcacacgtcactatcctcatcttcttccggttcaggccaataaaagccgggaatgtcgtacttcttgatgaagacataagacagttctgcaaggccaatacctataagacctagcaagagtcctatggcatacatcactatcaagtacatggtcatatggtacgcttctgtacactcaaagtcaacctacggcaaaggccgcctatttctaggcggccagttccttttcaagggcaatgactgttctacccttcaactcacgtaccggctttccttctacaaactcatagacggtaggcacagacatgatgtcgtaagcgaacttaatctctgggactctgtcaatgtccacatcaatcactggatagtctagcttgtctccaagcttcaacacgtgaggatgtagtctctgacaagggatgcaccatgatggtgctccaaagacaacgactgcccttctctcaatgtaggactcaaagtctttcaagtcctctacttttacgtagatgttacttccttccccaatcctgtactgccttgatgctaggcgcaagacgctcgacagcttccgcaaccttctcagggtcaccgctgacgtagatgttcacgttgatgacgataggagatgcatcggtctccttctcagcactgagagtcttgacgtcctcagcgtgaatgaacttcgtgtcatcactcagcttctcgccacggaatgaacccttgacgtactgagtgtagaaccggcccttgctcagtgcattggtaaatgcacggtagtcatcggcattgaagccagtgtacttgatgacgcttccagcccagaaatggacgacaagttcctcggtgccactgttccagtaagctgcctgaagtgcgcttgagttggtgccaagagcgttggtgtaaacgaattccatggtttccttctacatgtaggtgtaaattactttgcggagcctaaagtacagactccgtagtgggttgttcatcttctctttcttgtgcgaactgcgcatgatgtgcgtacacatccaggaagccagcctgtttaatcatgatgagtcgtcctgtctcaatccaggtaccatcaggctcatcttccaaacgcttggctacctcttgcagtacctcaaggatatcagctacagacggagttttcaacgtgccgtcgaatctccacttgtaacccttgagtctaaaaagctgggacagctccttagctattgcttcgaagtccatagtcccagcccttcaagatacatattgactgacgctgtcgcctgctcgacaattccaaacgcatcaatcgtgtcgttgtccacaggaacctttgacttaacaatggtttccttcaggattgcagacagcaaggcaactgagatattgagatttctgattgcctcgtcaggtgttgcaatgattgcactgtcaggcgtcagtacaaacgcttctctttcttccatttactttccggcgcggtacctctgcactaccagactgtaaagctcagcaagattgtcgtaagcctctttcagtcggagtgggtaaaggtccttatatctcttcgcatccttgatgtctttgagcaatgctagctcagtcagacgttcttggtgtgtgaagtcaatgcggggcctttgtggctcgcccaccgttcctcctattccatagcatgtctttgaatgtgcggagagcatcgttcgctccctgccagtacaagtgatgattctttgcgtaatcgcatcgaaccgctttttccaacttctccagcgtttccagttcgtacatgtcgttacagaactgcctgtagtccatttgctagctcctgtagagtaggtggcttgccgttaagacgctccagcatctctacgccgggcctcccctttcgtacaaccatcttagcatcttcagcggtccaatgtccatagacgatgccagcaacgaaccgagcagggaggaatcgtccgcccatccttgcctcgtatctagcataccgtctatgctcggcaaccgcaaggcactgcaatgcagccagaagagtatctgttctgacagttccttcccactcatcaggtgaatgagcagtaaaactttcagggtcctctccaaggacaacacgtgcatagagctgcatcaaatctctggcagcttgaggattcaagtctgtcttgtcctggaagtcatcaaagatttgctgatgtgtgaactgcttagactcccaacttgtggtgtcatcagtgatttcgactacgaagtcacctcctggtgtctctgtgtcattctcgtgcctacctggcatacgcagagtaacaccttcatatggactggtggaaagaatatgctttattcctccagtcacgtatggaatacgattattcaattcttcgaatctcattcttctccttacaatggcaaaaagccaggacaaagtcccggcttagagcttcttcagatgcttggggtcccacactgtctcatgtgtttgacccttggggtagtgatagacaggctttacacgaatacttccgtgctcgtcccatcgaatcaccctaacttgggtgccttcccagtcagcgatatgcttaggaaggttgtgaagaaccatgagcattccataattctcaccggcctcaagctccttctgatattccgcattttccttctccatcctcttgtcacgtttacgagacaggaacaaggcgatgggaactgcgattggccaaaaccaaatcagaaagaatacagtcactgttagctttgactgctctacggcaaatgtgtccatgttgtcttgattgaaaccaagacccataatccacgcaagaccaccgaagaggctcatgatgagccagccaatgccatacacaatcagtagtgctgttgtcatgtgttcatcctagcatacgaaggagtcggtagggaagggttgcgtaccctagtgctgatatatatacagcttcagcaagtgctatgaaattcaaggtgtccttctcgcatgtagaagattcgaccaggaaccttgtccggcacttcagaagccagcacgacaatgcccttgagactgtgactcatgaccattgccttagttcctacataagcctgcatgtcatcaggagcactgactacagtaacgaagtcactcctattgaccttccggccctttttgtcggtgaaactgtaattagccatgctcccctggcaggagtcgaacctgcgtacaccaaattcaaagtctggctacccaaccacgcagagtacaggggaatgggcccgaaggccactattagttaacggacattacatcgatgggccagtaaaaccactcaagaccctcgccatcgggcctctccgtatcaggctggacacgaagatagccagcaggctggtactcgtagcttggcaggatagttcccgtcagtccttcccagtcagcaggattttcgtcatcgtcccatggctctgtgtcgagcccgagtacaaccttgtctcctgttttaaacattaactcaccttttcgagattctgtgtgcgccacatgaacggattgtacttaaatccgtcaggccgttcagtgacgggaaggagccagttgtgcagatacccatcttcaaactgttcttcatcttccggccaaggctccaccattgcaaggaagccaatccactcgctgcggtcgctcgcagaagcgacgatgcgaacaacgtctcccacgaggaacagcttgtctgtgttcatgtcctcagtctagccaatctcatcggtgaagtcaagcagtgtccatccctgctgctccagtgcttgcttgtactgcttaccatgatgtccacagaacagcagagcttgcaaagccttctctgcaattacaaacgcttcagcgttgcatctgtcacacctgtcctcggtcttacggagaggacgctccatcactgcttccatcatcatgctccttacgatagcacaacgccctagcttccgctagggcctgtgttgttattctgctgtagcgctctcatggcagtctcaagttcactcttgactttgataaagttgtccatcaattcgtaatactttccacgccacttgtctaggtcggattcgacattgttcagctcttgtttgagtccctgaatttcagttctcagttcatttctgagctgagcggctgtgtcgtcccgaaccttgctacggctgagccagtgttcgacaaactttagtccaacaccccccagcactgtaccaatcaaggctatccaagccgtagtaatctcgggcatttatttcagcaaccttatcctaaagtataggaatgccacgatgagcgccaaggtaagactgaatacccaagtcagaggagtaaagccaatcgccatccatctgagaatagtcatgaatgagtaagagaggaacatcaagaagagtcccgctgaactaacattctctcgaacagagccccacaacgaagtagctgcactcatggtatagaagccacctatggacatcctgacgattgtagaatcaatcgcctgcccgattggtgtagtagcattcgcaacataccatgggcccactgcgtacaatcctgagaccagcaaaccgacagcgataatcaactctacaaccacgagaggattcttgaaaatcttgactagtgctgttgatatcctggacatatgcaaattgtaatcacagaagagttaaaagtcaatcaataaggtcttcgacagcgtcagctacttcttgcgccagccatagttctgtatctgtgagtacagagaatctttgggcccgatatctgtctatgtatagtcgcagaaatttagccctttcaacagcagatgctatttctcgttcatctgctgtggatttgaggaccaatttaatggtagcccaaatctctctggtcaaatcgactatgcattgtgctgtggatgactcatcttcaaaatacaatgttcctcccctcaaggggatgtcttgcggccggtagggattcgaaccccatagttccctagatgagacctagtatacccggcctgggggcttaattcaaatccccctgagcaatgtcctctccatcatatagcctctcagagagccaatggttcacatcctgcccacttcgtgccgccaaaacaatgagccatcgaggctccttaccaatttccttgcaatcgttgcatagattgaaggtcatcgtcttgagtagttttgactgcacagaggtgagcataaacctctgacccccacagctatcacaaactagttctctcactcttcctcgtatcctatttcgccaatcacttcaatatcatcgtgctcgaagtaatccgtgtgccacttaccctcgtcatcccaatactttacaaagcagtggaacgccaagacagcctggattattccgaatgcagtgccttcatcagtttctactctgacgacctgactctcccggtacatcatggacctccaatatacaatttacaccaagggaccggatggactgaatcagactgccgatttgttctgtaatcagcagcttctcttgttcggtgaggttgtcaaaggtgcttctgtatgctcgtagagcgaagttaggcacgtcgtgctgcgttgcagggtattgaacaaggtcacaggtaaaccctgtgccgggctcaaaacggaattgcctgaacagcctgcacgtgtcatcaacttgctgtagagcccatacgttctcggcactcatgacagcctctccttgattctcttccatacatctggagtcttatgagcgttacgatagttatctactctggcagcgttccaataaacaccaccccagactccaaatccattgttatcctttgccgccattgcgcactgcttcatcactggacagctcaggcacatctcatctacaaactttgcagaaatcttatcggtctcataagtctcgaagaagaaagaagtgggaagaccacgacatagcgccaggtcttcccactctacttcctctggacttatgcccctttgctcaagaatgcttgacatttttcctccttgcgtctcttgccacatttctacggctggcacgacacgctttacacatacgcctacctttgtactcgtatgtgttatcaggagtgaattcgtgaccctgcacgcatttagtaatcgttgcagcctgagccttacgattctttactgcattgccacgcctagtgttctctcccattgtgacctgttcaaggtgatgcggattgcaacactctcttaccttacacagatggtcaataactagggactcgttgagtgtcccgcctgccatttcaaaagtgtatctgtgggtctttacccacttaccgtttactcgaaacaaaccgtatccaccggcatcctttactgagttccaaatccaacactcagaggagtcaatggtaatcttttcgagaaatctatcaggcagaccgtacattgcggttgggaaccctccactttccatcggccccaaccgtcacgcgagtctggaaaccccagttgtcgttacggtaagcaccgtcacgctttgaccaaccggcctggttcttgctctcgcggaaaaagattaggttccattcttcccagcgaacgtcgtttccaagcgccttctgctcggtgacgaacgtgtcaacattgttgtaatctagaacaacagacataatagctttctcttttctttttgctgacaaagaacagggagaccgtagcctccctgttcaattattaagtctatgcttacttgctcttgcgagcggacgtctgctctgcactcttagaagttgcatcgtcagaggtgactacgccaccagttagctcgtccttttctactgagcgacggaaagcagcatcctcaaacttccaaccaccttcacgtgctcggcgagccgctagggtttcgacgtcggaacggctctggtaattctctagagcggcctcgtagtctacaaatgctgaatccttaacacccgcttccttagcacgcttcgcatcctcatctgccagtgggtcaagatgtggggctacaacgtcctgagaagtctttggcttgtattcatattctgccatttttactttcacctccttctcttcaagtctgcgcattctcgaagaattcttgttcagaataacacagacttgattatgaagcggtttactctggaaggattaccttcccagtcatagtctgcactgaatcgttctcagtgtcaaggaccacactgctgtagaagtcaccattcatgtccaaaccatatacactcgtgtaaccggtctggtgcatgaaagagtagttctccatccatgtgtgatagtgaccatggaaccacagctttggctgaacgactcggccaattcgattcatcagctctcgatgcatctgagagtccatgtcgttcttcagtcggaaccggaacggcgcacaggtaggagcgtcatgagtcagaaggtagtcagacttacgtccagcattctccagaccctttacgacattctcaggaaccttctcctgagcccaccagctcttaccgagcttacgagtgctcttgtcgatgcttacggctccaccaacagcctggaaccactttccatccacagcccagcgcttcacgcggcctgtgtagcgaatgtgagaacgaataacggtcagacctgcgtaggtctttgggttattcttctcgtaccagtccagccggtcccaattctcatgattgccagcgacgaagcagaccttcacaccatacttccggcactcgtcattgagagcatcaaggtagttgaatccttctacctcatggtcccaaagaccgaagtcaccgacctgcatgatgagctttgagtcatgttccacagccctgcgaaccatcttcctggcccatgctgtatcaccatgccagtctccacagaccagaatctttgtcatggcgttcctttcgttcgtcgttgtactaactctaacatcctgtagagcatcgtgtcaagagatgacgaaaggcccccgaaggggccccacatcacttgttctttggaatgaatcgaccattggcatcacgcttctgattcaggtgattcagtcgagtgtcaacagtacccttgacctcaagcagagtatcaagcagaccatcggcctcactgtcagacataccactcagacgctcacgcatgaacttgtcaatccgagactcttcctcctcgacagacagttcaaatccgtagtcatcatcctcgtcctcatcgtcagcgtagtcgtaatccagaggaccagcgacaggcgcatcaatgacttcaagaacagtgtaacggcaggtacgcagcttctggtggtcacagtcggtcggaacagaaacaacatcccgaggattaacctcaaccttcagcacagctccacgagcaaagttgctcgcgtagccccacgtaccaacgtgaaggcccgtgtgacaacccactgaagggtcatgctggaccttatcacgaggcatttctacaatggctcccagagggttagggatagcgccagtgtactcaacgccatcagagatggcagtaccatgagaaatactctcgtaagtttcagaatagcttgcgccgtcagcagagacctgccgattgacacgaacacccttgtatccaacgaagtttccattgggaaggatggtgaattcattcgtgttcagccagtcgtaaagctggtcacggctgtgcggattctcgtttgtctgcaccttctcgaagaaggcgacaagaggaccgaagtcatcctgtccatccttcaggaatcgaacaacctggttcgtcagagcgttgtccacaggctgattatcccagtagacctttccacccttgacagtcactcggtcagaaagacggtcgaaacgagtctgcgcggtgtaggcagggtcaaacaggtcaatgacattgacgtcatcagcctgaaccccagccttaatggcatcccagttcagatgctggtcagtggcagaaaccatacggccatcgtagaagaccgtaatgacctctccaccagcattccgaatcaggctatactggagattagacattgttttcctttgcgtagacagtgtttgcgtagatgtagaagtgttcgagcatctggccaaatcggtcggtaaccgcagccatcagaggatactttaccttcaggctcttgaccttgtcaaccgcagggcgagtgatgttcagtcgagagcagacgttggctacctcgttgaagtggtcaattgcgtctgacttcctgatgctctgtactaccttagtgtaccgcttcagttccgggtcgtcaagacgctcagggtcgaacatcatgagaggaatccggtcgtagtagtcgatactcatgctcagcttctcgtcatcagagagaacactgaaagccttggcaaccatgttggcgattgccttgtcaacctcaattgcattggggttttcacgaaggaacttcgcccaacggttcttaggcagacgaattccgacagcatcaccatgcagcctgacaatctgaccaatcatgtcttcgtacttctcttctccagaagtccagagaatcagagtctttgacttgtcaatgtcagcaatggtcaactgacgaccatatcgctccccaggctgtgaaacagtataagtctcagaggcaacgccacgagactcacgaggcttctttgtagcaaagacctcttcccatgaaccaacctgagtagaagtaaaccacttctgctccaccggctcctttgtgacaatgaactgacggccagcaaagttcttctggtctgcccacagccgcatcttggcacggtgagaagtagaaatctcatccttctcatagccatgaatgaagatgcaggacttcagctcattgaagttaagagcgctgtacttggcaatgctaccacgaccatagtcaggccggtaccgctggtgagtcacgtcaagattctcaggaatcgtgtcacccttataggtaaaggtgtaacggtttccccagccagtcattgtcttccactcaagccactctgcacgagcctctgcatgcgtagcctttgagtcaatggcttcctgagccttttcaaagagcctggtcttaacaagctcctgaacgacctcgatagtctttaccgtattgtcggtgtaatgcaggtcttcacgagaaggcgtgaagttcacgctgccgatgtcaacctttgcgacaatactgaagttacggccacgatagccataacggtcctggtaaagagcatgctcattgctgacacggtaaccgacattgcccatgacaatgtaatctacctgtccagaccgaatagccttgattccgtcaccaaggtcagttccctcaatgtattccggctcggccccatcaatcagaacagtgccaggctcccagaatcggaagaaatccttagccttccactggaaatcactcgtctggtggacaggaacggaaatctcaacaccgttctcctcatccgtctgctgagtgtgaacaacctgcataacaccagagccatcctcggtacgagagatagcaacgtgaaccaggtatccatccttgacagaacgaacggtgaactgctgagtgtaagtcagagcagacttacatccgagcccaagcatgccaacctgagcattcgtggaacgctttgtagatgcaccgtacttggaatagatgttgcgaatatcctcgacagacagtccaactccgtagtctcgaaccttgaaaaagggagacaggccgttgggaagggaaacctcaatcggcctctgcactcctgcctcgacgtgagagtcataagcattggtgctgtactcacggataaccgcaagaattgggtcggagtagaggtcagtcagaattgacataacatgcgccatgcttgcagcatcaacgctcattgcgacagtttcaccagtgaggttaccctcaagcttggcgaaggttgcagtaggctccatgattcatctccttgtagtgggtgagcttactttgttgagtctagcagacggaagtggtgtacacaacccttgccacaccggccgcgttcagagccctctcacagcccctacaaggctttgaaaaacactcctggccactcttgttccgggcaatataaacagtcgctccagcggcctcacggcagcgtgcaagggccatacgctcggcatgcacagagcagaactgctcaatgtgctcatcactgacgttgttcgggtcgttcttgagcatgttccagccctgagacataaccctaccacccttgacgactaccgctccatgtctctgcttcatgttactgttaagactcagtctcattgccagagacagaaaagaccggtccttgttagaaagaccggttaccttctcctttaaacattgatttcctccacgattgtgtaacaggcaatttcagtgtgaagaattcgatgaatcttttcaccgtttaccggttccagcctgatgtggttctctgcaataaattctggcagagagtcagggtcattagatgaccaattgccgatgttggtgacccaattctttcgttcatcaccgtgttcattgacccaggtgtagtttaccttcaccatgacatcgtatttcttcaacttagccattacttaatcggctcccattcaccagcgtagtaccggtctaggtcttccaccttaccgtcttggtcaaggatacggaattgtccgttatggattgcacctatgaccgccgtgcggccggacgtatgtttgatttgcatctccacacgcatgtcgctgaatgtcatttagtatttcctccactgcgtggtccatgtatctcagggccagtacgttacctacgataccaagtgcgatgagcagtgtcaaggccctagcgactcgcatttaccaccctgatgatttctgcaacggttcgaatgctcctgacgaacggctcatccattaccagctcaccgtcactcggtatttgccagcaggaatgcggtcttcattggccagaagattcagtacgaattctagaccaggggcagcatactcctcgcgaggcatgccttccttctttatcttctcaatcagagcataggcttcatcacggtcgtgagcttcatcaatccacatagcctcacgttcgacctcatttgaatcaatgcctacaccaaaccatccaccagtgacatcaacgtctacataggtattctgagcagggaagcccattgcagctccaccttcccacttgatgccccagacttcctttacgtaattctggatgtcttggtaccaccactcaaaatagcctaccatcttaggccctacgagttccacgcttctcctttggtagattgcggtccttcataatctttgaaacgtcagtcatttcgtgctgaccacgcctcgtctttccctttgcatgcaatagcagagcaagcagcagagcctcacctgtgtggatgttgcctgacttgtctctcatgtacgctcggtccatcatgcgatttcccatgggaatccacttctttttcacttggcctcccttcataaagtcctagttcttctgtgattcttaccaaaaggcaaagaccacacacgccattctccatgtagtctgaagttgacatatgcggtccgcattctgagcaattgatgtaatacacgcggaaggaggaggattcgaaccccgagccttttacaacccagttggttagcaaccaacctgcgagacctctcgctcaccttccatagcctcagttgaggcccatacgaatgctttcttccattcccttgatgaatatctgagtctcagacgctcgcttatctggaacgaaaatgcctcgtttctctacttccttgccgccctttgacagaatgagttgaagagtctttccattcatgccagcgaacattgggtcgtcaatttctttgacttcccactcatagccttccggcgcttctgatagattatacatgtggccctgatgggactcgaacccacattgacattccttttgagggaaccgcatatgccattctgctacagggccatgggacgagacctaaatctcgtcagcgcaaatgctgtcacattcaacgcagtgccacatcaaagtgtcatcgtcgtgaatatcctggttgtatgggcatacagaccagaagatatcaacgccgactgcatacgtcttgccagtgattgcacaacacgcacacttacgatacagctttccttcgattttcattttgcctccttgtgttgggtacttttcctttcacttggagggcatcatgattagtcgttgtctacataacgaaggtcattgatgttattcacacatgcaccttctgcgaaaatcttatctgagttgatacaggccgtaacttccttggtgtcaaggtagttagcgtaactcacaccaccagcgatggatacgaacagagcaatgaataccgttgcgataatcattgccaccttaacttcagtgctactcatttcattccttatcgttggaagtgcccactggaagaatcgaacttccacgccttgcgtgtcgagcaagtgctctaccattgagctaagcaggcatagcccgaaggctttaaactgtgaggcgcttcttcacaccagcgtaaaacttattccactggtcgacgttcttgacctctccacgctgctccttgtatgcctccatgtaggagacagcagccttgcgctgttcatcatcagccttacgagccagcaaaatcagctcagcgacatcgagagcaatagttcctactgacttcagagcttcagaaatctttgacagctcgctcatttttctcctagaaaccgattccgccgcttgtccaaacgacgaatactacgatgaaaaatagaatcacagggattgtgattccaagtacaatgggccagagcattctttgcctttcatttgcttacgtacgccgactgggattcgaacccagactatcagccgtctcaggactgcgactcctgccattggtctaccggcgcaaatgcctgcttacgcaggccgaacatcttcaggcttacagtatacatcactgccgttaccaaggtcaagcagaagaaagccatggtcggttacaaactttaccgtgacaatcttgccatcatactggccacgaaggatttctacctcgtccttttctctgtatacatgaaccatcagtcaaactcgaatccggactgtcctccaagctccttcatgattcgcgtctccagctttcgtccttccttgctgtcaacgtaacccttgggcttcttttcggagatttgtccattgtagtagtaatagatttgagtcgctggagtcccattggcagtcataggaatcttctggaagaatgacttcttcttgaatataacctcgaagtagacataaccatcaggattcgtatcaacgtagtgagacttgtacctgtcagcaatctcttgacgaacgtaatacgtcgtattgagcttgcatcggttgaaggcccagcgaagtgcctcgttttgcagcttacgtccggcgcgagaagtcgcactgagtggattccccacgccggacagagcgtccatgattcccatttaatcagtcctcgtcaaacagctcggtaccgttcttgacacagacccactcgttgtcgtcgaccttgtgcttgttggacttcttctttccgtggccgttaccaagtccacttcccaggttccagcccttggaagtcttcttgttctgcttgtgaccaccacttgacttcggagccttgtacaccttcggagcagccttaggggcagccggacgaggcttggggccaccgatgtcaaacgccatcggagccattcccaggttctcgaagtcgtcctctcgcatcgtcttgccaccagcagccttgcagcgcttggcaaaatcagcaggcggcttgtcagaacagccggtcagtccgaagaccagaccaaccgcaacagcaccaccgaggacagttgagctaactcgcttgttcatttcgacccttcgtcgttgttgttggtactactctatcggttggcttggctacttgtcaagaggctcgtagtcggcagtctcatcaagagggaagtttccaaacatgtggagtcttgtgtacctctcaaggtacacctctagccacacttctgcgagccgatggctctggaaatctcccttgatgactgttcggtcactgtcccatccattgctctttagaccatacacgatgtatggagcctttgagcccatgggaagctgcgttccttctacttcgaagccgctgaactgttcggggttcttgtacatagctacagcctactctctcattccgggctcgtcaacccctggctccatgacatgaccgttctcgcactcaatgtcatcggcaggcacatgacctccacactcaaaacaagcatagcacagaggcatgcccatctgagcaaactcagggtcacgacagatgtaacagttgtcacgatacagaccttcaacaggcacactgaagatgtctggatgttctagcaaccagtatgcagggtcgctctttttcattggctgtgacatttattcctcctatggcaagagggccccgaagggccctctttttacaaatccttcaaaccgtctttcaacgccttcatctttacctcagcaagatgaatgtatccgtcggccacccgagcaagggatgctcgatggtctgaactctgtgagtttacacgcgcatggtccaggactgattcagcctgctccagcagttccttgactgtcctcactggtttcctctcagaagtttcctggctgtacctggaagcagtctacaccaatgctgcgccacatttcaaccccgaggcaggagctgaatctcaatctcatctcctactgtacctcgtggaaacgcttcgtacaacttgtgccaccagcttgggatataactaagcaattcatttgaagtgaggttagacttgattgtgtttgccctcattgtacacagcacaatgttgtccctggtgtatcccttccatggaagaaccttatcaactgaagctgagtttggcaagagccccttgccccagccgatttccattgggatgtccatataaaaacattttccgtcttgcttgacccacaagtcttccaagtcttggacagttaggtcaaaatcaatcccaagttcttttgcacgacccttaatcttgcttaccttgtcaatccaaggattgtaactcttccgtttttctttcctcgcctcagaatgacatggcttacatctgtttagatagtaaccctcacggtctttccgcttattgaaaggaaattcttcaatctctttttcctccccacattctaggcaggtcttaaatacaacattacccatgagagtattctctcacaggtaatgttgtatgtcaaacggggcaagttgtcatcagaagtcgccaggagcgacctgaaaacaatccacacccattgccctccacatatctacgacttgctgcctatcgtcgtagacagcaactacatcgtagttgtgccggatgtgcttgtcaaacagctccagtttgacgatgttgtccttacgcatgtctccctcagcgcgcatgtgaatttcatcaaatggcacaccgtagaagttcagccactccattgtctcatttcggcagacagcgtcacggccgctcatgacgatgattccgtaccctgccttggcgtgagcctgagcagttgcaatcacctgagcacgagggtcgtcatcaccgacacgaggccagtcaaagggagacctgtcagtcatagcggcaagggtaccgtctacatcaaagaggtaaaccttcttcttacccaccggccgaacgtagtgctcgacagactcagtgtttccccaagcatcagagatgtgctgacgcgtgtactgaccagaagacttgagagacttgtacatcttctgaatgacatgctcgggaacgtcacgtccgcccagtgaagcacgaacagcgtttcgagcgtatgcagttgccaggtcgacatcgaattgtttgacagcaaccggaattcctcgcttcgcagcaatctttacgaaagctgagatgtactggaaacgaatgttggtgtcatcgacaacaacgctgtatccagcagccagaagaatatcaacctgagcatgctccagttctgtaaccatgccctcatccacgcccacttccttgccgaagtgctgcatgcgaatgtcatcacggtttacacgggcccgacgctcaccctccctgagccactgctgagcaaaagtgctcttaccacatccgggaatgccccggagaatgagaagcgtagccatttacttatccaatcttcttgtacagttccacgcaagcgtcatcgaatgcgtcctgagcagcaagccaacgcttgccggtttcttcatctacagtgaccttatcacaaccgctcatctcctcaccataggttgccaggtaaggaccctcacgatagaaccatacgtccattacttacctctcttgtacgcccttcggcgcttccagtcttcgatgagtcctactctaggcggtccgtcgatgtttgtcaaccagtccatcgtaccaaacagaaggccgtagactcctagaacgaagcctacggcaatcagtacgatagcaccaacgacgatacccacaccaatgaggataccttcaatcattgctatctctttcctagattacttgggtgccaagggcaagtgagttcgtggtttggatacggctgaccgcaatccatacacttgaacgcttcctcacccatcctcgtcttcggcaagcatcccatagactcggtcttggcaggactgacagagaccagagattttggcctcacgtcgagacacatcatctttgaagtcttcctctctgataggttgtccacaacctataccttgagtacatatacctgtggcgacagcctttttagctgccgcccgatattgacgcatcatcgcttagccgatgaggaaaccgactacgaagaacagaccagcgagaatcgcaggatgctcagcgagccagccccagacagcggaaccgatgaccttgaggtttgaaacagcctggtcggcctgagtgtcagacatttgttttatttctccttgtttaggagccttctccgaaggctgccatgtttccgattagcgtatcagttggtcgtgagtagtgcaagtgcatccgttccaggttctcgatgtcagcaccgaaacttggaaggttctttgtttgtagttggtgaagctcgttagcgtgctgcttcgcctcgttcagtgagtctatcagctttttcaagtaatgggctcgcttccagcaaagcaaagcagcatcgctacgccgctttgggtccttacgccactcttcgttagcttgtcgcagggtaaccatcctccatcttggccactggcttcaactgcttccagactagctcatcgatgctgttcccgtcaagcaacgtgaacatgtgaggtcggtagtggtctaccttgttggcctccatagcgaagtctcgccgtgtgaagccctctggcattgaagaaagaaggctgtggaagattccacgtaccttaatctcaatgtctgcgaagtcgctcaggagctgtccaccgacatccttgacgaactggtggaactcgtcaggcagagcctcacaaatctcctccagcgtgttgccctccttgagctggctccatacagagcgctctgagagcattgaaatcagtcggtgtagctcaacatagtcagcctgcttgagcttgaccatcttggagccagaacgaattaccagaccctcagcgttcgtcctgtagtctgcctgaacagcctcatgaatgttcttgtaagagaacacctcagttaccggcccgttccaatccagcaaagcctgagcctcacgaggaccgtaaacgtaaccgtgctccttattgaccgctcccagaaggatgaggtcatccatgtctccgtagtccaggacgattcggttgtcagggtagacaatctcgaaaatgaaagtccagtcatgaggcacggcagtgttcttgtacttctccagccacagcttcgtagcgtgaattgcctggtctgatgcaaagcttccacgagtagcgaagctgtactcatgaccgtaaggcggcatgtcagggttgtaaggacgaatggagtacagaatacccattgagccgtccttcttgtccgtcacctcgaccgggtcatcccagtcaatgtgcgcagcgtgagagtctcccatgttgaagaacttctcgaaaggccgagcaatgacattgtcgtcctggtcgatgataaggccacggcacgcaagcgtgcaagcgttccaaagaccacggaactgagtctgttctgaatagttcaggattcgcaggggaaggaccggatgagtctgcacccgaacaaaaccctcgttgaccatgcggtcaagcaagtcctgagagaaaatcttggtgaacttcatcggtcctccttgttagttgcccttgattcttgagaccagcctatcagacttgaatccgttgttcaagcggttgatgtaggcagaagtaaaatcggccggaacaagacgcctgccgattcgctgctcaacgtgttctacacagagcataccttcgtgcccaagccctgtcaaggaccagacacgcatatcgagaaagaagtgctcgccaatcttgcctgtgtctataccgcaatcaaggcagagccacttcttacgactgttcttgttaccggccaccactcaccttcacacaatagatttttagcatgtgcgctagttctgggttgtctttctttgcctgttccagatagggcaacaacgttcgatgctctggccagaaaggctgattggtgtacttgcctgtgtagcagtttctcaaagcttctgcgtggtcacaggcaattgcgtacatcttagtctgcatgtgtcacttcccgtaaagcatctcccaattgccggaaacgaagtcgaagtccttggcgtcgagctttccgtaaagctccttgccgcacacaatgcacttgtggtagaagagtccgctgtacttgttgtacaggaccatggtgtgcttacgcccaaccttacccttgcaccacttcttggtgttcttctttccgcctccgccaccacgaggtgcagagctttcggtgtagttacgagcactgcgcttgatgttcttgccaagcttccagtccttgtaaccacgttccttaccagtagccataataattcactccttcaaagttaggtggttacctaaccagggtcgtaaatcattacgctccttccactcttcgaattgatagtcataaagtgccaagggcaggaatcgaacctgcaaccttccttctctgtcattctagcagcgctactagaaggtgtccagccagcgtgcacactgacaacacactatagagaggctgtgcttaccaaagacacttcctcggcttgtgctgacgtagatggattcgaaccaacaacagtcctggccaaggacatgtgctttgccaattaagctatacgtcatagaccctttcgggccgggcttttacttaccgaacttgttcttggcgaaggtctcgaagtccttcttcgtgccaacgacgtcttcccacttgccacgaccaccgtgaaggcggaagttggtcttgcggttggctgccttgctggtctcaacgattcccttccaggaaccattgacgtacaggttgaaagtaccgtccttgtcggtcttgcgaagctcgaaagccttaaccttggcgctcatttatcctccataaagagagtacacgttatcgaatgtgagattgtcttggtgttttgagaagaaccgttggctcatgatgtcttcaccaggcacaacggttggcttgggggcgattcgctgaacttgcttcatgagtctatcaccgaacccctgacgcctgcaagcccttcgtgtatagtagtgggcaaccatcttgccttctaccttttcgtacacaagggaccaggctatgagacgttcatcttcgtctagaatcaggatagcagaggctttgcttctttcgccacgcctagcgaacataagttcttcttgcatatagcctccccatcgaaggttgaggctatagcattttctgtactcctgtggagtcagctcaccaaccggctttacaaccgtcttggtacgcatcttactcctttggttcaaaccccttgaagacaccggggttgttgttccagggcttctgcccctctgtgataagaagaatagcagtcacttcaccgaaggtcaagccatcgctgtccatggcccgtccaagggccatctcaagtgtccactcgtctgcttcgtacgtcctcatcttgtcactccaggtccgtatctgtcaaccatgcctaccaaccaatcacgaccgattggattcatgctatgcacgtagaccgtctcaggccagaagtcgtgctcacacatccacaataccacagcacgagaggtgtcgtcaccacctaggtcatggtcaagtgagatagcttctggagtgattcccttacggcgacaggcccgaagcagaatgattgcaatattgctatgcttagttgcaaaccaactgttatcgggccttgggcgctcgtcgtcgagccaaatcttaataggattcactgacggtaccatcctcgaatcttgccaagggaacctgaaactcatccaaagggtcgtaacgaactgccgtgatgtatgactggccattgctgtcaaaactgtgctcataccggtctcgaatcatggtatagtcacgctgctccggccagttcatttcgcaccaagcgacatcaccaacgatacgcttgacggtgaagtcaacctgtgcattctgatacttccaacgcgaaccaatttcaggcattactttccccaatgctcaggctttacaacgacttcacgtccttcaagcatggcttccttgatgtggtcgagcagaacgtcagtcgtgttggcaccgaacttcggctttccttcgacgtacacagcccaaccatagaactctcgaccgttgatgtcaagtcctggcttttgcattattcctccttgtggaagtggggggattcgaacccccgtcctgacgcttcaaacagtagtctatacacagccatgtgtcaagcaggtactacgtgcgttttccccgccgggccttactgtcagtggattcttctgttccaaggccatccactagcccggctcagcagccgtcaggctgcaagagcgaatgcaacgttagagttggcatttataggtttgagactttttagcgacaccttctcaatgtcgggctgcaaactaaagctattcgcaccagtcgaaaccggtcactcccatagacgagtcacctttcggcgtactcatccagatactcatatgctagcttgttccaccgggcgtcgtcaagtgctctatgagcattggcttcttgctttggcagacctggcttgccctcgttgtgccagcgctgcttgatgtccatagtatacatcggcatgccatctggcaagtcaatcatacgtccgaacaactgacttacaacaacgtggtcgtaggctccatagtaggcccacagctcaggcttgtcaaactcgtactctggagtgtagtcgactacgaagtctctccatagtccagcaatcacctcgttgggcagcacccgatgatagtcaggatgctgagtgtcccatgcaaggctcccatcgtcgctctgctttaccggcaaatgagcgatgacgttgtccctcagccactcattctcgtttgctctctggagcacgtagaggttgtttgtgatgagatacagctcctcaccatcctcacgtaccgctccaaggctgataaatcttacaggctcgtctggtcctgtctcaaggaactcggtatcgtagaatgttttcaaggtttaaatgctccaatgttaaatagaaatccatcaggctcgaaggctagataaatctttagtccgtttgactcgtagccttctaccacgatgtcattctctgttccattctcaatcttataagggtcttcctcaaactgaataccggcctctttgagaatcttcagtattctttctctatgagtaaggtcgtatgtcaaaccgccttctccttcccaccggtcgatttcgtgcatgttaaatcacctacaaaatgaattaagacccaagcctattgacttgggtccagcactcccgagaggattcgaacctccatgtagtcctttaacctttcaactggttcgtagccagagggtatacgagagtatgaagaatcttagcgagtcttgctcttgcgtgacttccaccaaagaagtgcaagagggatgtatggcttagccttcagatacagggccttgagccttagcttaattgtattcataacgagcctcgtgtgggaatcgaacccacgaccttttccgtaccaaggaaatgttctaccactgtcactaacgaggcataaaggccgttcacctgcaaccagctactagaccatcctcgatttccttcaatgcttcagttgcattctccacaccgtcagtcttcgattgagccacgtgtcggtttcgaaccgctaaaatattccaagtattaagggccaccaacaaaccatcctgcacgcttatacgttacacgacgatggcagttagcacatcttacttcgcacttagcaatctcggcttgaatcatttccagactattgactctaatcatttttgagacgttgtttagcttgtctcctcttacatggtcaaactctagaacgattgggtcagcctctccacagtcaacacatggatgctgtagaaggtagtcccatacccattgcctgtttctctcaattactgccttagcagcagccttcaagtaactagcgcgattgtcatttgttctgtaatacgaattgtcgtagtctctcttacatggcttgcatctaggatacttgccatccttgctacgcttccaattaccaaactcatcgagaggcttggattccttacaaccagtgcatgtcttcatacctgtattgtaaagtgtatcgaattacaatgcaaattacaagagcctcgtgtcagaatcgaactgacggcactactttacaagagtagtgctttaccattaagcgaacgaggcatagccatcatagcacactacgctttacaagagcgtcgctctacaccaaatgagctaacgtggcaagtggttccgttgtccagcgggccaacggtttcctaaaaccctcagagacactggagagaatcgaactctcaacacatggctttgcaggccagcggcagaccaacggcaacagcgtcattaactacattacatgatttaaagctcactgtcaagcttaccagccagattgaagataagcttccctgtcatgatgtggtccagcttaccaccggtttgactatagacagtgtactcacagtttgttctcagtacgaaatgttcagtcaagacaactagacacgcttcaccttcgtagtaggtcatttcgaatggcacagaatcaatctctccaccaaccgtataaggaccttctccaaatgagtatctaatatctatctgcatcatttcagcttcctggaatcatcgtggtatgttgtggacaatacgctgcaacagaagcaccggcaaagtatgctgagtcagaaagtgtcaaaccggtatccattgctgtccatgagactactgactggaaagtatcttggttttcagcatacacacagacattatgtccatggtcaacaagagcttcgtctgtctcgcttttaatgccagatatttctgctctcactgtcctaacaaacgctttgtctgtagttgtaagttctcttcccttgtcttcgtgttctgttccacaaccaacaaggacgagtagcatagcgatactggctgctattctcatcctattcatgtggcacctgtgggtatcgcgcccacctctaaagcttttcaggctatcgctaatctatctcagctaaagtgccatggtaagtctgcttggggtgattggcaaggactttaaccctgcatgttcgggctaacagcccgactcattacgcttatgattacaaccaccacagagtatcctttgcccctcggccgggcttcttgttcagtctatgcgtccatagccaagaagtcaagctgtaggcttgcaggtaggatacaacccgcagggtgactagagggtaccgccccctcttctcaggattcacagtcctgaacattacttttatgctatagccaccatggcggtgttattctggcctttcaccaggaggcgaaccgcagaacctcattgcgtggatgtagtaggagtcgaacctacttgatggataagacgggttacagccgcctccccgaaaccgttggggcattacatccagatgggccatgccctttgactcattgaattcctccgtcgagttcatcgcagaatcagcaggcttaaccgcatggcgcgtgcggctcgacagtcgcggatgtcagcccagaaggggaattacttcccgctcagctaaacgtttttctaggggacccctagctagttaaggtacgttattaccccttgcgtgcatgccgtcagattcgaactgactcacccgaaggaacggtgttacagaccgtcgcgcctctccatctgcgccgcacatgcataaccccgaagggcttcgccttagtctatcacagtacgattgggcgatgcaaccggaccggttcgaggaatattccccacaatgccgaagtaactcttgagaacatcaattgcatgaaccatgctcccaaggtcatcgccatagatggtgtattcttcggttccatcaggaccggtttccaggatatctcggtagtagcttggctgcacaccatcgagagtctcgatagcttgcataacatcttccattgtgtaatccatgctactcctaatcaactacgtgatagatatcccattggtgagtatacttgactcttcgaaactctacctcaatcctgccaggcagtttgtttctctttagagccttctcaaaatcattgaccatcttgtcacgctctgattcagagtgtgccgtgttgagatactttacactcttaccgtgcatttaatctccttgaaagtcagtgaccgcgcctcttcgcagtcttgccagtctgtggaaccttccccttcatcaggtcaccaccacgctgacgcttctcgtccagggtaggacgcttgacggcaccaggggtagacataggaccaggcatttttactcctcatagaattgtacttttgttgttacgtgggggcagaccctgattcgaacaggaattcgggattatgagccccgcgtgataccgtttcactaatctgcaagtggttgaagatggattcgaaccatcgagtcgaatgacgggcagtgcacagtgcccctgtttggccaacaacagttcctcaaccttgtacgtagcaatagctggattcgaaccagcccctcgaccttatgagagtcgttggcacaccgagcgccctactgccatataagtgctttggggtgagttactggcttctcagcgttagctccacctattcaccttcggcactactccacattatcagcctcagacggagatttttgtctttgctgtgtgtgggtacgttcactctaccacacatccccgcttccctgaacgccaccagcgctgttacctgccagtttgagcgggtttccctgatgactactactctacatcatccgagcgactgtgtcaactcttgaactcgaccggaccaacgtccatcttgtgagaaccgtcagagtaaagaaccggacgagcctcgccaccccgagagtagtcctcaacgtgcttgatgccgacctgaccctctgtggtgaaatcaacaagagcaatggcaacccagtagctaccatggttgttgttatacagaggagccgtcacagtctgtcccttgccgtccgtaacaacagccttggctccgtaagacttgaaagaaccagcaccattgctgacagcagagtaagcaacgatgagaacataggcgatgtcgttggtattctcgatggtcacagtctccttgccaggaacaagagagtcaccgtgatgacgaataccatcagcaaccaggttgttccagtagacagcaccaccagcatccttgcggaacttgttagccttgctcttggtcacatacagagcataaagctcaaggtctgcacctcgggcacgacggctagaacttccaccatcccactcaagagtagcagtgaccttcgcccccttttccagactgaaagcagaacttccgcccttggtcagattgagcttaccggtagagaccgcaggcgcagcctgagtcgcagccgcatcgttcgtcttcttgctaccgaaaagacccattgtgtaactccctgttactagttgattgaatgaaagtggggaagtaaggctagtccacacgcatcccacagtcttgttgagtatcattaagtgggcatctaagttaccaccgcttgaactactctcatactccaaagccttgtccgactgcttacttcccacgtcttatcttatcacacagcttacagtctcttttaccgttgctgcgattaaacacgttatctggagtcaactcatggtttctgttgcaatgtgtctttcgagcattctttgcaccagcaccattgccacgcattacgttttcaacgttggtgactgcttccagatgcttaacattaacacacagtccgttcctgcacaagtggtctatgaccaactcatcaggaatcttaccatgtactttctcgtacgcatctctatgggcaaggaccatcttcttaccaggcaagataccataagtattgccgtgggttgtgaaggtgtttccggtccataagatgcatggaccttgaaccactatgctccttgtccgtgtgcctgtctttagtctgacatgccagtgtggctatgtcaagtgccccttgagggattcgaacccccaaccaacagcttcgaagactgctgctctatccattgagccaaagaggcatggtactactttacttcaaggtccctctcattgatgtgaaaggttccttcattggtcttaactgtacaggttttcaggctagtcatcaatacctcaccagtcttacctttgttaggaccgttgattacttttactttggttttgtttgcgaacttaggcattacttccttcgcttcaggtcattggttcctagtcgagtctgaccaccagagtcaaggataactatcacccaccggccgtcttgtgactcaactgtaccaggctcagtgttgttgtttcggatgaccttgtcacccttcttccatatcttgttacccatgtggtccaggtgggatttgaacccacgtcctcaaagattaaaagtcaattgcactaccagactgtgcgactggaccataaaggagaagctagacagcaggttcgcataatgaccccgccttttcttctcagttacaccacattgtttaccactgggagtcgaaccccaatgtgattaagcacccttgtaatgatgctttgtactccgagccggactcgaaccggcgacacgcagattaagagtctgctgttctaaccaactgaactaccggagcaaagcgacagaggtttgatttgtagccgaagccatagcagccctgccgagtcatctgcatagtttataaaaagctagctcgggatgactagtccctcactgcctacccaccatgctagcacatgtagtctgtgggttgcaaggactgctacgggctattaccttgctgtgtcaatcagcctgctagagctgacccgcgtgtttgctaacctctctggtagaaacactgaaagagtggagacgatgggattcgaacccacagtcgcttgcatgccatgcaagtgcattaccgttatgctacatccccatgtgttgactacactaccctgtgccgggcgtcttgtcaacggtcgtcgttcttgcgacgtggtctcactctaccatatccagtcacctgtccacgaaaccttagaatcttctccttggttccgagccgtctgaggctgattatctcttcatcgttcagaatctcatagaactttgaaggttcgtcttcgacagcttcccagatatgacctacaacaattctgttcactgagcatcatcatccgtacggtcacgaatgattccaaggaacagttcgtgataatacctcactgagatattatcagtaccgagccttctgacccagaccctgtcatcatcgatgtcatcgatttcaaacactcgtctgccgccctcaacaggctcccagatttttccgcatcggattctatcattcatgtatccccacccagactcgaactgggattaccactttaggagagtggcgctctatcctttaagctatagggatgaggcccgaaggccactctcactcgttctctgcgtcatactctacagcaagcttggcaacaagtccaggggtgagaaccttgattcgcttgttgcccttcatcacgatgcgctggatgcctccgtcggttgacagagtaagctcattgtcactcttgttcatagaaacctggttaccaatgacttgtccctcttcgatgtggtcacccacactgagattcttaacagccttggtttctgcatgcttctttccaagctgccagcccagcttagcaccctttgcagtacccatcagagctgacatggcaagcttaccgcttccctttgccgcccgaattgcaccagccttggcaagctccttagacttcgccttgatgtccacgctttgtccttatcagtttgaatcgattgacttgaacttcggtaccattagatagtaccaggacgaatgacttaccgtcaagtgccctgcgtgatgcagctacttgaccaagccctgtcacatcaccgggctttattgaaaaagcaaacttcattgtatgcctcctgtttatgtttcaacaggatagcatacctacgcaaaatggtccaaccgaagtcggaccattgcgagagcagattgaagctatctcactgcgagctgacctagcaggattcgaacctgcaacctgatgggtaacaaccatctgctctaccaattgaactataggccattgtgatggagagggcatttccaacccactattgtcaataggatatccatcttaccctttggaagggatgcagcactccccctctgagcggggtctcttgctttaagcggtcccaacagccgctcttttactgtagcacgaccaccctcaacggcttcctcttaggtgggtatggccgctaagccattcttcatctccgctcccaagatttaaaaagccgtaaaaccaagggtacacgtgagaatctacgtgctgtttgtaaaactatgtggtcacctggtgtgcgtaggcaatgtgaaagccaggccaaccaggaaacttgatgtaataaaacaattcatcaccaattcggcgctgctcgaaacactcgcccctgatacctggctgaggtcccttgtgattcttgttgtcccacgcagcttccttcaaagttaccttgaaccgcttgtctgaacggtaacagccgaactcttcgtcccactccatgcgccatcctttgatagaaatcaaactgatgtggaccggacttgcaccggcactacccctacccttgtagtcaaattccgtaggggactttccaggttgctggctgcctcttgtagttgggctaccacatcaaattggtggaacgttgccggtttatccgggattagtaccatactctcatgcacatagtatctagaatacgtacactaccatttggatttatccgtgccctgtatacgagctttggcgttccagctctcacggtaggagtcgaacctacgaccggcagattaacagtctgctacaccgagccacacaggtgtatcgtgagaatagccctttcgggcgtttttcaaaagccgttgagcttgttctgataccaggttatcacatctgaaagaacttctcgaatcctgtcgaacagatacatcgtactcctatggtagcaggtcacttaccgcatcggcaagtccttcgaggatgtcagcaatccagttaccattgctgccacctcgtgacttgctcttgtctgttggtactactctagcacttacacagcgtctgcacaaggccgggttcttgtcgaagtcactctggggcttgtctgtattacagccccagcacttcatcttgtcgttcatcgaactggacataagtcaggtctcctggggttaccttctgaggtagtctaccatcactgaacagatgtatgccagctcgcttgtaggcttcatctgctagttgagagcatatcatgtgctttgaggaagtgacgtagttctctacccacttccacttgataccaaatctctctagtgccaaggctgcgtagtcaaggaagctgtaaggcactccttctagctgccgggcttctcttacgatagcggctcgctgctctggtgtcaacttgatgtctacatagactgccagagggctgccgtacttggtagctccagcatacttgtccaggctatcaataatagctccaccaggcatcgcctctaccagctcgccattgtccaaaacaacaaacacatggctgtatctgctcgcgtctcttataataaattgccctaggccaatcaggacaccaacgaggccacctattcgggtgagtccgatgtctccaggctgtggcgtatacttcttagtcatacagacattgtagcataaaaaaccctgcccacgagggacagggtggattttaagtgattcgggtcggatttgaaccgacgtcctcaaaggttaagagccaattgcagtaccagactctgcgaccgaatcatgtgtcagggttacactctcccatactcctaagtacctgacatgggcatacgtttattatac
